# Spatiotemporal expression patterns of anxiety disorder-associated genes

**DOI:** 10.1038/s41398-023-02693-y

**Published:** 2023-12-13

**Authors:** Kalyani B. Karunakaran, Ken-ichi Amemori

**Affiliations:** Institute for the Advanced Study of Human Biology, Kyoto University, Yoshida Konoe-cho, Sakyo-ku, Kyoto, 606-8501 Japan

**Keywords:** Clinical genetics, Molecular neuroscience, Psychiatric disorders

## Abstract

Anxiety disorders (ADs) are the most common form of mental disorder that affects millions of individuals worldwide. Although physiological studies have revealed the neural circuits related to AD symptoms, how AD-associated genes are spatiotemporally expressed in the human brain still remains unclear. In this study, we integrated genome-wide association studies of four human AD subtypes—generalized anxiety disorder, social anxiety disorder, panic disorder, and obsessive-compulsive disorder—with spatial gene expression patterns. Our investigation uncovered a novel division among AD-associated genes, marked by significant and distinct expression enrichments in the cerebral nuclei, limbic, and midbrain regions. Each gene cluster was associated with specific anxiety-related behaviors, signaling pathways, region-specific gene networks, and cell types. Notably, we observed a significant negative correlation in the temporal expression patterns of these gene clusters during various developmental stages. Moreover, the specific brain regions enriched in each gene group aligned with neural circuits previously associated with negative decision-making and anxious temperament. These results suggest that the two distinct gene clusters may underlie separate neural systems involved in anxiety. As a result, our findings bridge the gap between genes and neural circuitry, shedding light on the mechanisms underlying AD-associated behaviors.

## Introduction

Anxiety disorders (ADs) are the ninth most prevalent health-related cause of disability [1, 2], affecting 3.8% (285 million) of the global population [3]. ADs exhibit moderate heritability (32–67%) and substantial familial aggregation, with the first-degree relatives of AD-affected probands highly likely to develop a range of AD subtypes [4]. This suggests that there is a genetic basis for the clinical diagnosis of the different AD subtypes. AD subtypes have 48–68% comorbidity [5], suggesting that they share genetic risk factors. The genetic underpinnings of ADs have been previously examined using targeted gene sequencing and genome-wide association studies (GWAS), which identified mutations in (or in the vicinity of) several genes and captured multiple aspects of anxiety critical in understanding its etiology [6]. RGS2, PKP1, TMEM132D, and BDKRB2 were linked to diagnosis of panic disorder (guided by the Diagnostic and Statistical Manual of Mental Disorders or DSM), THBS2 to generalized anxiety disorder and GLRB to agoraphobia. SLC6A4, COMT, HTR1B, and HTR2A were linked to responses to drug/psychotherapy, PDE4B, NTRK2 and NPSR1 to DSM-guided binary diagnosis of anxiety and/or composite anxiety measures, CRHR1 to neuroticism (AD-associated personality trait) and NPY to neural activation patterns in response to anxiety-inducing stimuli [7, 8]. Although these genes have been linked to AD, if and how they contribute to AD remains unclear.

Physiological studies employing functional magnetic resonance imaging (fMRI) and positron emission tomography (PET) implicated a specific frontal-limbic-midbrain circuit in AD symptoms [9, 10]. Similarly, microstimulation studies in rhesus macaques suggested specific neural circuits causally involved in AD symptoms [11, 12]. Although these physiological studies have been able to associate specific neural circuits with AD symptoms, whether the spatial distribution of AD-associated genes converge on these same circuits remains unclear from previous analyses of AD genetic data [13–15]. The spatial distribution of gene expression in the brain can be correlated with functional connectivity between its various regions [16–21], and examining the regional expression patterns of disease-associated genes can help identify the brain structures and the molecular mechanisms underlying neuropsychiatric disorders [22, 23]. We thus hypothesized that the regional specificities of AD-associated genes could correspond to the functional organization of the AD neurocircuitry implicated by physiological experiments.

To address this, we integrated more than 200 AD-associated genes from GWAS across 4 subtypes of ADs and mapped this information onto microarray-based, spatiotemporal transcriptomic data extracted from over 200 brain structures of normal human brains available from the Allen Brain Atlas [24]. Taking our integrated approach, we show that AD-associated genes can be split into two clusters showing distinct spatial expression profiles in cerebral nuclei, the limbic system and the midbrain. These clusters, likely underlying distinct neural systems of anxiety, were involved in specific behaviors, signaling pathways, region-specific gene networks and cell types, and exhibited negatively correlated temporal expression patterns. The spatial distribution patterns of these gene clusters corresponded to the AD-associated neurocircuitry identified in physiological experiments, thereby bridging the genes-to-neural circuitry gap linked to AD-associated behavior.

## Results

### Determination of AD-associated genes across 4 AD subtypes

**Fig. 1:**
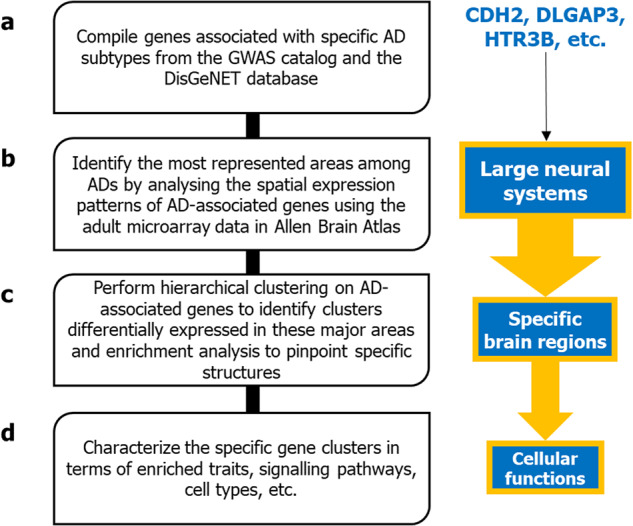
Methodology to understand the neurobiological implications of AD-associated genes. **a** Genes associated with four AD subtypes were collected from two repositories that contain data on disease-gene associations. Next, **b** Human Adult Microarray Data from Allen Brain Atlas was used to systematically examine the expression patterns of the AD-associated genes across 232 brain regions belonging to 13 major structures in the human brain. The major structures in which the AD-associated genes agglomerated were pinpointed and **c** hierarchical clustering was performed based on their expression profiles in these structures to isolate spatially distinct gene clusters. **d** The biological attributes of these specific gene clusters with characteristic expression profiles were identified.

To examine the spatial distribution of gene expression in the brain, we took a systematic approach to analyze the expression of AD-associated genes in the human brain (Fig. 1). Two-hundred forty genes harboring mutations associated with four AD subtypes, namely, generalized anxiety disorder (GAD), social anxiety disorder (SAD), panic disorder (PD) and obsessive-compulsive disorder (OCD), were collected from the GWAS catalog [25] and the DisGeNET database [26] (Supplementary Fig. 1 and Supplementary Table 1). The genes compiled from the GWAS catalog were found within 50 kb upstream or downstream of the SNPs associated with the AD subtypes. Genes harboring SNPs originally listed in the GWAS catalog, GWAS DB [27] and the BEFREE database [28] were compiled from DisGeNET, with gene-disease association (GDA) score ≥ 0.01 to ensure that at least one publication supported the GDA. Overall, we attempted to capture the full spectrum of AD genetic architecture to identify AD-specific regions (Supplementary Note 1).

### High expression of AD-associated genes in cerebral nuclei, midbrain, and limbic regions

To confirm that the 240 AD-associated genes were selectively expressed in the nervous system, we compared our dataset with nine other organ systems (see Methods) using two independent datasets from GTEx [29] (Supplementary Fig. 2a) and the Human Protein Atlas (Supplementary Fig. 2b) [30]. Our analysis revealed that the AD-associated genes were tissue-specific (Supplementary Note 2).

Although AD-specific transcriptomic datasets are available, they have limited overlap with one another and are from disparate sources, such as post-mortem human brain tissues, blood samples and pharmacogenomic animal models [15, 31–34]. Therefore, we examined the spatial expression patterns of the 240 AD-associated genes using the adult brain microarray data available from the Allen Brain Atlas (see Methods) [24], as seen in previous studies of neuropsychiatric and neurological disorders [35–39]. Normalized microarray data of 29,130 genes from 3702 dissected brain samples of six healthy donors were analyzed. We categorized these samples into three tiers according to the hierarchical classification of brain structures in the Allen Brain Atlas neuroanatomical ontology [24]. Based on these tiers, 232 samples were collected from tier 3 structures, which spanned 13 tier 1 structures, i.e., cerebellum, cerebral nuclei, diencephalon, frontal lobe, insular cortex, limbic lobe, medulla oblongata, midbrain, occipital lobe, parietal lobe, pons, temporal lobe and white matter. The intermediary tier 2 grouped tier 3 structures into anatomically distinct subdivisions within tier 1 structures (e.g., cerebral nuclei, amygdala and anterior amygdaloid area are tier 1, 2, and 3 structures, respectively). We sought to pinpoint the regions showing high expression of AD-associated genes. To accomplish this, we compiled a list of genes showing higher expression in each of the 232 regions relative to others (as per computations described by Rouillard et al. [40]), and checked their distribution among the AD-associated genes using hypergeometric test (*p* < 0.05 after Benjamini-Hochberg (BH) correction was considered significant). We then identified the major areas in which AD-associated genes clustered, by mapping the enriched tier 3 structures in each AD subtype to their parent tier 1 structures. This revealed that AD-associated genes were enriched in a larger number of tier 3 structures that mapped to the cerebral nuclei, midbrain and the limbic system, compared to other tier 1 structures (Table 1), making them the most represented areas among ADs. This remained true both when GAD, SAD, PD and OCD-associated genes were considered as independent gene sets (Table 1a) as well as when combined into a single gene set (Table 1b). Hence, our subsequent analysis focused on cerebral nuclei, midbrain and limbic regions, since AD-associated genes were selectively expressed predominantly in these areas.

**Table 1:** Major areas enriched among AD-associated genes.

Level 1 brain structure	Number of tier 3 brain structures showing significant association	
	a	b
Cerebellum	0/38	0/38
Cerebral nuclei	**14**/21	**7**/21
Diencephalon	6/35	2/35
Frontal lobe	1/28	1/28
Insular cortex	0/2	0/2
Limbic system	**8**/16	**4**/16
Medulla oblongata	3/14	1/14
Midbrain	**10**/17	**3**/17
Occipital lobe	1/13	1/13
Parietal lobe	2/14	1/14
Pons	7/14	3/14
Temporal lobe	0/17	0/17
White matter	0/2	0/2

The number of tier 3 structures in each of the thirteen tier 1 structures that show a statistically significant enrichment of (a) individual AD-associated gene sets (i.e., by considering OCD, PD, GAD, and SAD associated gene sets separately) and (b) a single AD-associated gene set (i.e., combining OCD, PD, GAD, and SAD associated gene sets) at *p* < 0.05, after correction for multiple hypotheses using the Benjamini–Hochberg method. The number of enriched tier 3 structures has been shown in each cell, in comparison with the total number of tier 3 structures mapped to the specific tier 1 structure.

We sought to examine the relationships between different AD subtypes using two approaches. First, we assessed the gene overlaps between AD subtypes using a hypergeometric test, and found significant overlaps between GAD, PD, and OCD (Supplementary Fig. 3a). However, differences in gene set sizes of the AD subtypes (ranging from 9 genes for SAD to 147 for OCD) could have affected the significance of these overlaps, and produced unexpected results such as the overlap between SAD and OCD, rather than with PD, contrary to clinical observations [41]. Therefore, in an alternative approach, we analyzed the correlation between the expression profiles of subtype-associated genes to identify the connections between the subtypes. The analysis revealed an expected positive, albeit moderate, correlation between PD and SAD (Supplementary Fig. 3b). It also revealed positive correlations of these subtypes with OCD, indicating that they possibly share a genetic basis despite the lack of a direct clinical correspondence (Supplementary Fig. 3b). Most importantly, GAD showed negative correlations with OCD and SAD, signifying the presence of distinct gene groups with unique expression patterns specific to individual AD subtypes.

### Bifurcation of AD-associated genes based on differential expression in cerebral nuclei, midbrain and limbic regions

**Fig. 2:**
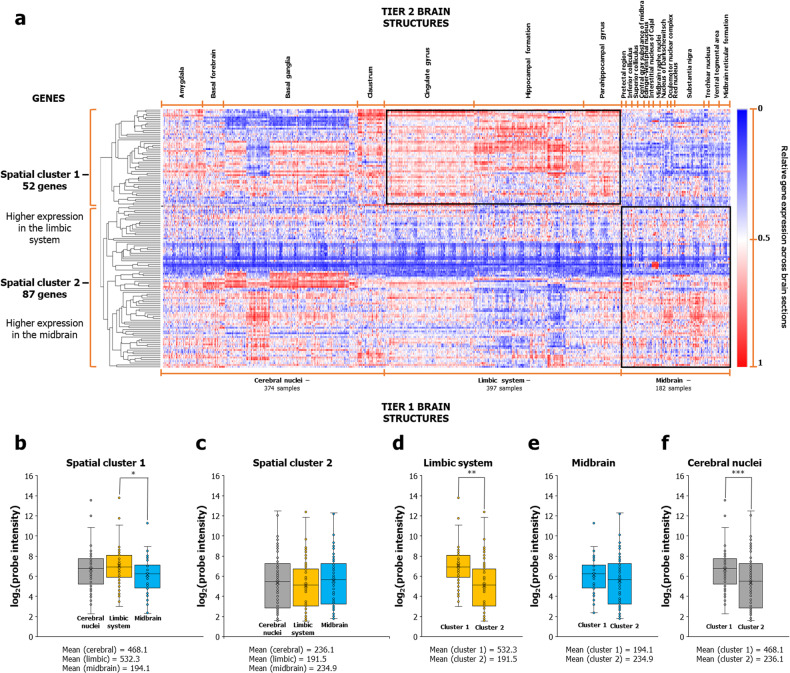
AD-associated genes bifurcated into two clusters based on differential expression patterns in the limbic and midbrain regions. **a** The figure shows the dichotomized expression of 139 AD-associated genes across 374 cerebral nuclei, 397 limbic system and 182 midbrain samples in the Human Adult Microarray Data (Allen Brain Atlas). Relative gene expression of each of the genes across the 953 brain sections was hierarchically clustered by computing pairwise distances between the data points (log_2_-transformed probe intensities) using Pearson correlation and identifying closely linked clusters using the average linkage method. The clustered heat map was generated using the Morpheus software. The box plots show the differences in the log_2_-transformed average expression values of genes in **b** Spatial cluster 1 and **c** Spatial cluster 2 across cerebral nuclei, limbic system and midbrain samples. In **d**–**f**, the box plots show the differences in **d** limbic system, **e** midbrain and **f** cerebral nuclei samples between spatial cluster 1 and spatial cluster 2. The statistical significance of the difference between the means of the box plots derived from the Mann–Whitney *U* test has also been shown. *, ** and *** indicate *p* < 0.5, <0.01 and <0.001, respectively. In each box plot, the central line indicates the median, the bottom and top edges of the box indicate the interquartile range and the whiskers represent the maximum and minimum data points. The means of untransformed probe intensities have been shown at the bottom in **b**–**f**.

Next, we sought to further clarify the spatial expression patterns of AD-associated genes. To address this, we hierarchically clustered the expression profiles of 139 AD-associated genes in cerebral nuclei, midbrain, and limbic regions. A matrix containing the expression values (log_2_-transformed probe intensities) of the genes in tier 3 structures was used for performing hierarchical clustering using Morpheus [42]. Pairwise distances and closely linked clusters in the data matrix were calculated using Pearson correlation and the average linkage method, respectively. The heat map (Fig. 2a) showed the relative differences in the log_2_-transformed probe intensities of AD-associated genes across various brain structures. Interestingly, two immediately identifiable gene clusters showing distinct spatial expression profiles emerged (Fig. 2a). The first cluster, which we named as ‘spatial cluster 1’, contained 52 genes highly expressed in limbic areas and specific cerebral nuclei (Fig. 2a). The second cluster labeled as ‘spatial cluster 2’ contained 87 genes showing lower expression in the limbic system and higher expression in the midbrain and specific cerebral nuclei (Fig. 2a). Quantification of expression levels between the two clusters in these regions showed that spatial cluster 1 exhibited higher mean expression in limbic samples than midbrain samples (Fig. 2b). Conversely, spatial cluster 2 showed higher mean expression in midbrain samples than limbic samples (Fig. 2b), albeit in a statistically non-significant manner, possibly due to the larger variance in expression (Fig. 2c). Comparison across the two clusters showed that spatial cluster 1 showed higher mean expression than spatial cluster 2 in limbic (Fig. 2d) and cerebral nuclei samples (Fig. 2f). In short, AD-associated genes bifurcated into limbic-associated spatial cluster 1 and midbrain-biased spatial cluster 2, suggesting that potentially distinct neural systems underlie anxiety. Note that we refer to spatial cluster 2 as “midbrain-biased”, since the expression trend is consistently in favor of the midbrain but not statistically significant.

**Fig. 3:**
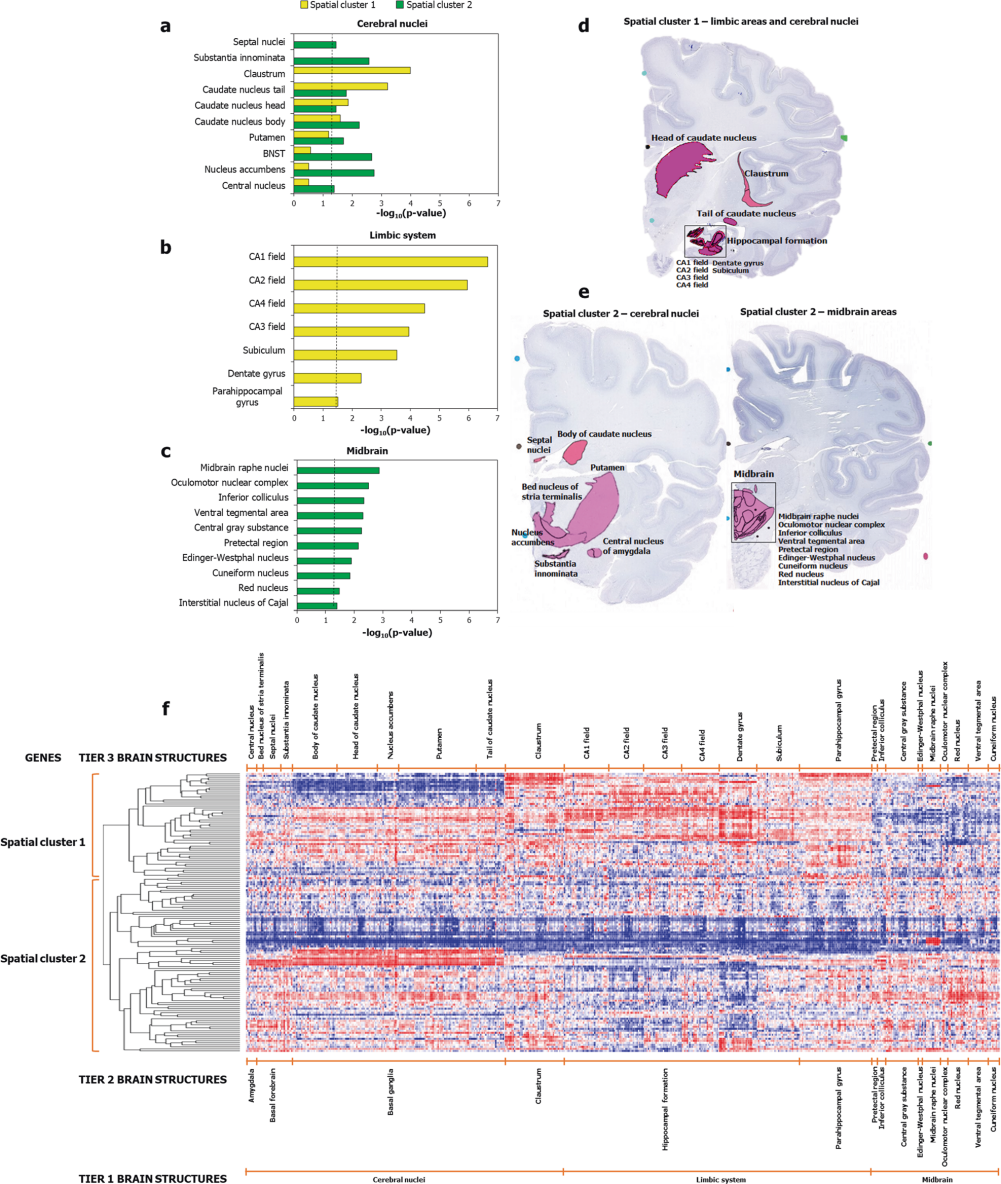
Spatial cluster 1 and spatial cluster 2 showed differential enrichment patterns in specific regions. The figure shows the enrichment of spatial cluster 1 and spatial cluster 2 for genes showing relatively higher expression in **a** cerebral nuclei samples, **b** limbic systems samples and **c** midbrain samples, compared to other regions. The dotted black line in **a**–**c** indicates the cut-off value for –log_10_(*p*-value) after correction for multiple hypotheses using the Benjamini-Hochberg method (*p* < 0.05, log_10_(*p*-value) > 1.30103). **d**, **e** show the specific areas in which spatial cluster 1 and cluster 2 genes are highly expressed, highlighted against the background of Nissl-stained images, namely, the specific **d** limbic areas and cerebral nuclei in which spatial cluster 1 genes are highly expressed, and **e** cerebral nuclei and the midbrain areas in which spatial cluster 2 genes are highly expressed. **f** Dichotomized expression of 139 AD-associated genes across the enriched regions shown in **a**–**c**.

Next, we sought to interpret the complex expression patterns of the two spatial clusters shown in Fig. 2a. To address this, we asked whether a large number of genes in a particular spatial cluster was significantly biased toward high expression in any of the 232 tier 3 structures and computed this probability using a hypergeometric test (*p*-value threshold set at <0.05 after BH correction). This helped us pinpoint the specific regions associated with spatial cluster 1 and spatial cluster 2 (Fig. 3). We found that specific cerebral nuclei were preferentially/exclusively enriched in either spatial cluster 1 or spatial cluster 2 (Fig. 3a). Furthermore, we observed the exclusive enrichment of spatial cluster 1 and spatial cluster 2 genes in limbic (Fig. 3b) and midbrain areas (Fig. 3c), respectively. Figure 3d, e shows the enriched areas highlighted on Nissl-stained images. These enrichments were derived using the hypergeometric model, which evaluates the frequency of genes with high expression in specific brain structures within the spatial clusters, compared to their expected distribution among brain-expressed genes. To validate these findings using an approach that directly considers the raw expression values of the AD genes in conjunction with their numerical overrepresentation in specific regions, we employed gene set enrichment analysis (GSEA) [43]. First, we ranked genes by their expression levels in cerebral nuclei, limbic, and midbrain regions and computed enrichment scores (ESs), which indicated the degree to which highly expressed gene sets in specific brain structures were overrepresented at either end of the ranked gene list (corresponding to GSEA 1 and GSEA 2). We then permuted the GSEA cluster labels, recomputed ESs to create a null distribution for ESs, and calculated nominal *p*-values relative to this null distribution. These *p*-values were corrected for false discovery rate (FDR) to produce *q*-values, which were used to assess the statistical significance of the regional specificities. GSEA 1 and GSEA 2 exhibited enrichment for subsets of the regions enriched in spatial cluster 1 and spatial cluster 2, respectively (Supplementary Fig. 4), except for globus pallidus. Nevertheless, the globus pallidus is closely associated with the cerebral nuclei identified in spatial cluster 1 by the original analysis (Fig. 3a). This refined region list obtained with GSEA confirmed the ability of hierarchical clustering to derive two different AD gene sets with distinct spatial expression patterns.

### Recapitulation of the spatial clusters using AD genes supported by transcriptomic evidence

The 240 genes considered in our study as AD-associated were selected due to their genomic proximity to AD-associated genetic variants. Pinpointing the specific genes affected by the variants is challenging without additional evidence confirming the impact of the variant on gene expression or the dysregulation of the gene in AD patients. To address this, first, we examined whether any of these 240 AD-related genes showed altered expression patterns associated with their respective variants. We utilized the FIVEx database [44], which provides the summary statistics for expression quantitative trait loci (eQTL) associations of specific SNPs and potential eGenes within 1 MB of the SNPs from various sources, including BrainSeq [45], GTEx [46], and ROSMAP [47]. We applied a threshold of *p* < 0.05 to identify statistically significant SNP-gene associations across the whole brain or specific brain structures. Additionally, we examined whether any of the 240 genes exhibited differential expression (DE) in blood samples or post-mortem brain structures of AD patients using the BaseSpace correlation engine [48]. Only two AD datasets were available, one with the expression profiles of (dorsolateral) prefrontal cortex samples of OCD patients versus healthy controls (GSE60190 [49]) and the other with the blood expression profiles of GAD patients (GAD score 7 ≥ 5) versus normal subjects (GAD score 7 < 2) (GSE61672 [31]). Genes with a fold change >1.2 or $< \frac{1}{1.2}\,$ were considered significantly overexpressed or underexpressed, respectively, at *p* < 0.05.

We found that the expression of 52 genes were significantly modulated by their corresponding SNPs (Supplementary Data File 1), whereas 29 genes were differentially expressed in OCD patients (Supplementary Data File 2). In addition, 24 genes had both supporting eQTL and differential expression evidence. Altogether, 43.75% (105 genes) of the 240 genes in the proximity of AD-associated variants had supporting transcriptomic evidence. The expression profiles of 92 of these 105 genes were available in Allen Brain Atlas. Hierarchical clustering revealed the bifurcation of these 92 genes into two clusters, eQTL-DE 1 and eQTL-DE 2 (Supplementary Fig. 5a). These clusters not only respectively overlapped with spatial cluster 1 and spatial cluster 2 (Supplementary Fig. 5b), but also recapitulated their regional specificities (Supplementary Fig. 5c–e). The regions derived from this analysis are subsets of the regions identified for spatial clusters 1 and 2 in the original analysis (Fig. 3), with the exception of the lateral nucleus. The striking overlap between the eQTL-DE clusters and the spatial clusters clearly indicates the presence of two distinct AD gene clusters with differential spatial expression profiles.

### Validation of spatial clusters from AD-associated genes using independent datasets and methods

Next, we examined whether we can recapitulate the two distinct groups of AD genes even if we use independent datasets distinct from the Allen Brain Atlas microarray data. We thus utilized the RNA-sequencing datasets from BrainSpan Atlas [50] and GTEx [29], containing Reads Per Kilobase Million (RPKM) and aggregate median Transcripts Per Million (TPM) values, respectively. Interestingly, the hierarchical clustering of these two datasets also yielded two gene clusters. The derived gene clusters significantly overlapped with the original spatial clusters 1 and 2. Specifically, hierarchical clustering of the expression profiles of 134 (out of the 240) AD genes from BrainSpan Atlas across 26 brain structures revealed BrainSpan 1 and BrainSpan 2 (Supplementary Fig. 6a), each respectively demonstrating significant overlaps with spatial cluster 1 and spatial cluster 2 (Supplementary Fig. 6b). Similarly, clustering 171 AD genes from GTEx across 13 brain structures revealed GTEx 1 and GTEx 2 (Supplementary Fig. 7a), each displaying statistically significant overlaps with spatial cluster 1 and spatial cluster 2 (Supplementary Fig. 7b). On the other hand, we could not derive any regional specificities for the BrainSpan and GTEx clusters because they lacked the spatial resolution of the microarray data and did not include samples from the specific structures we had originally identified.

**Fig. 4:**
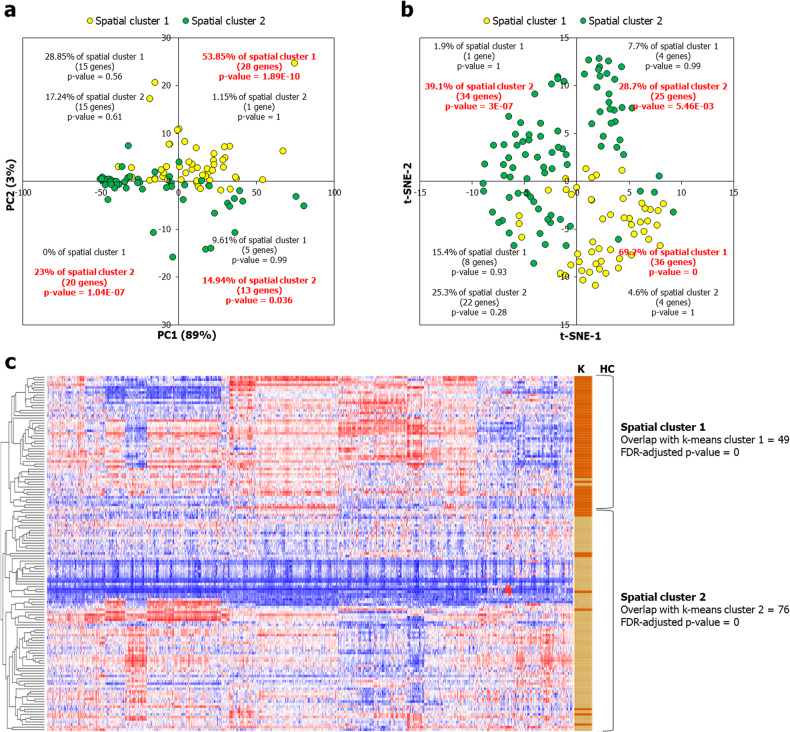
Independent clustering and dimensionality reduction methods recapitulated the spatial clusters. **a** PCA was performed with the probe intensities of the AD-associated genes in cerebral nuclei, limbic system and midbrain samples in the Human Adult Microarray Dataset (Allen Brain Atlas). The probe intensities were log_2_-transformed and a data matrix with brain regions (rows) and AD-associated genes (columns) was constructed. Unit variance scaling was applied across this matrix. Singular value decomposition with imputation was used to extract the PCs. Component scores (*n* = 139) corresponding to PC1 and PC2 explaining 89% and 3% of the total variance were plotted along *X* and *Y* axes, respectively. **b** t-SNE plot constructed based on the gene expression of 139 AD-associated genes in cerebral nuclei, limbic system and midbrain samples (metric = 1 – Pearson Correlation Coefficient, epsilon = 10 and perplexity = 30), color-coded based on their membership in spatial cluster 1 (yellow) and spatial cluster 2 (green) derived from hierarchical clustering shown in Fig. 1a. In **a** and **b**, the *p*-values of enrichment of the genes in each quadrant for spatial cluster 1 and spatial cluster 2 computed using hypergeometric test have been shown. The *p*-values have been corrected for multiple hypotheses using the Benjamini–Hochberg method. **c** On the vertical axis on the right side, the clusters of AD-associated genes derived from the k-means method (metric = 1 – Pearson Correlation Coefficient, number of clusters = 2 and maximum number of iterations = 1000) have been color-coded based on their membership in spatial cluster 1 (dark brown) and spatial cluster 2 (light brown) shown on the left side.

Next, we analyzed the spatial expression profiles of the AD-associated genes using alternative independent methods – namely, principal component analysis (PCA), t-distributed stochastic neighbor embedding (t-SNE) [51] and k-means clustering [52] – to determine the validity of the spatial clusters retrieved using hierarchical clustering (see Methods). PCA recapitulated the two spatial clusters along PC1 (which captured the maximum variance in expression) (Fig. 4a). Approximately 54% of the spatial cluster 1 genes were found in quadrant I (*p* = 1.89E-10) of the PC score plot (Fig. 4a). On the other hand, spatial cluster 2 genes were segregated into two quadrants; 23% of the spatial cluster 2 genes were found in quadrant III (*p* = 1.04E-07) and ~15% were found in quadrant IV (*p* = 0.036) (Fig. 4a). In order to pinpoint the specific samples that strongly contributed to the grouping patterns of the genes as seen in the score plot (Fig. 4a), we examined their loadings on PC1 and PC2 (Supplementary Note 3). This supported the association of limbic samples with spatial cluster 1 (Supplementary Fig. 8a), midbrain samples with spatial cluster 2 (Supplementary Fig. 8b) and specific cerebral nuclei samples with both spatial clusters 1 and 2 (Supplementary Fig. 8c). Additionally, the tier 3 regional specificities shown by the spatial clusters (Fig. 3) were recapitulated by the PCA quadrants I, III and IV (Supplementary Fig. 9 and Supplementary Note 4).

Using t-SNE, we found that ~70% of the spatial cluster 1 genes localized to quadrant IV. Spatial cluster 2 genes were segregated into two quadrants; 39% of the spatial cluster 2 genes were found in quadrant II and 29% were found in quadrant I (Fig. 4b). 94% of the spatial cluster 1 genes occurred in k-means cluster 1 and 87% of the spatial cluster 2 genes occurred in k-means cluster 2 (Fig. 4c). Hence, both t-SNE and k-means clustering recapitulated the two spatial clusters derived from hierarchical clustering.

### Behavioral traits, signaling pathways, cell types and gene networks associated with the spatial clusters

In order to understand the functional implications of these spatial clusters, we examined their enrichment among genes (a) associated with specific GWAS traits, (b) involved in specific synaptic signaling pathways using Gene Ontology (GO) biological process annotations and (c) that serve as markers for various brain cell types.

**Fig. 5:**
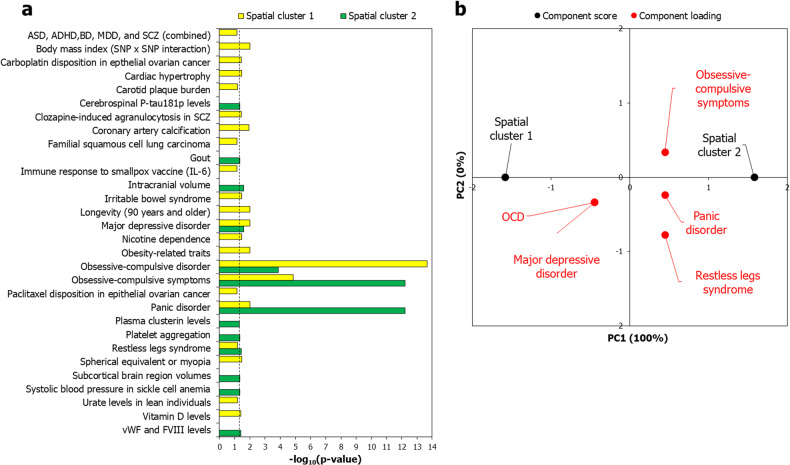
Separation of the spatial clusters were influenced by specific GWAS traits. **a** The figure shows the enrichment of spatial cluster 1 and spatial cluster 2 for genes associated with GWAS traits (in terms of –log_10_(*p*-values)). The dotted black line indicates the cut-off value for –log_10_(*p*-value), i.e., 1.30103, after correction for multiple hypotheses using the Benjamini-Hochberg method. *P*-values from the enrichment analysis were transformed to –log_10_(p-values), which were then assembled into a data matrix containing the traits as rows and the spatial clusters as columns. Unit variance scaling was applied across this matrix. Single value decomposition (SVD) with imputation was used to extract the principal components (PCs). **b** Component scores (black dots) of spatial cluster 1 and spatial cluster 2 corresponding to PC1 and PC2 explaining 100% and 0% of the total variance were plotted along X and Y axes, respectively. Component loadings (red dots) of 5 dimensions, i.e., traits, contributing to PC1 and PC2 were plotted along *X* and *Y* axes, respectively.

The spatial clusters were significantly enriched (at BH-corrected *p* < 0.05) for 30 out of the 1737 traits found in the GWAS catalog (Fig. 5a). Next, we determined the loadings of the –log_10_(*p*-values) associated with these traits on the two clusters, which were separated using PCA (Fig. 5b). This showed that only 5 out of the 30 traits were influential in bifurcating the two gene clusters (Fig. 5b). OCD and major depressive disorder loaded positively on spatial cluster 1, while obsessive compulsive (OC) ‘symptoms’, PD and restless legs syndrome loaded positively on spatial cluster 2 (Fig. 5b). These associations were further supported by the enrichment ratios shown by the AD subtype-specific genes in our study, though they were statistically not significant (Supplementary Fig. 10). See Supplementary Discussion for possible explanations regarding the loading of OC symptoms and OCD (as a categorical disorder) on distinct spatial clusters.

**Fig. 6:**
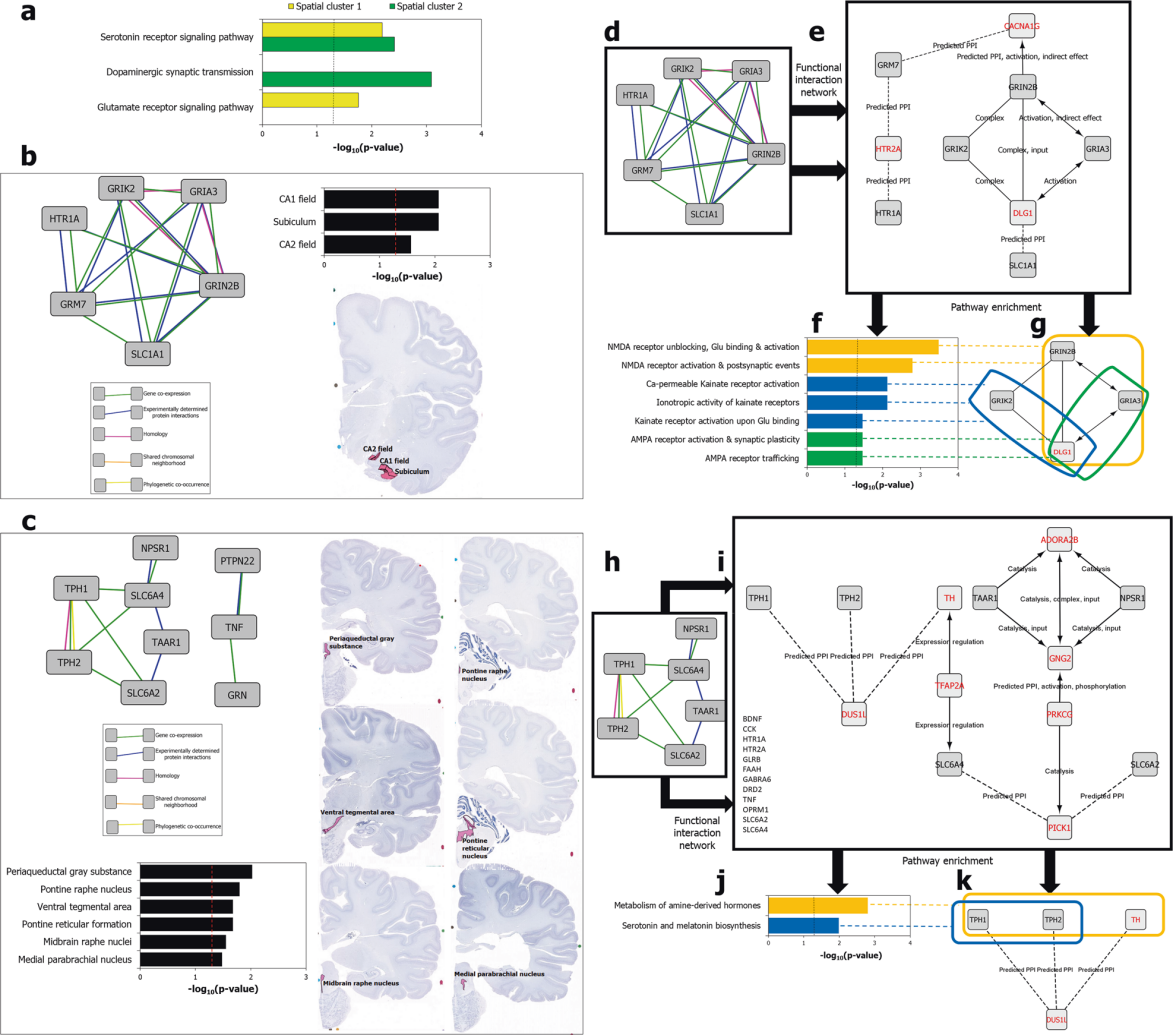
Spatial clusters showed enrichment in specific synaptic signaling pathways and contained region-specific gene networks and pathway interactions. **a** The figure shows the enrichment of spatial cluster 1 and spatial cluster 2 for genes involved in synaptic signaling pathways (in terms of –log_10_(*p*-values). The dotted black line indicates the cut-off value for –log_10_(*p*-value), i.e., 1.30103, after correction for multiple hypotheses using the Benjamini–Hochberg method. The gene networks enriched with genes present in spatial cluster 1 and spatial cluster 2 have been shown in **b** and **c**, respectively. Network nodes represent proteins and edges represent protein-protein associations ranging from joint contribution to shared functions to direct physical interactions retrieved from the STRING database. As shown in the legend, the colors of the edges denote the various types of associations. –log_10_(*p*-values) of enrichment of the gene networks for high expression in specific regions have been shown in **b** and **c**. The dotted red line indicates the cut-off value for –log_10_(*p*-value), i.e., 1.30103, after correction for multiple hypotheses using the Benjamini–Hochberg method. The enriched regions have been highlighted on Nissl-stained images extracted from the reference atlas provided by the Allen Brain Institute. Using ReactomeFiViz, we isolated the functional interactions that exist in the spatial cluster 1-enriched gene network shown in **d**. This functional interaction network can be seen in **e**, in which → indicates ‘activation’, -| indicates ‘inhibition’ and – indicates ‘part of the same complex/physical association’ and dashed line indicates predicted protein-protein interaction. In **f**, the enrichment of this functional interaction network for specific Reactome pathways has been shown in terms of –log_10_(*p*-values). The specific proteins and functional interactions in **e** that are responsible for the pathway enrichments shown in **f** have been enclosed in boxes with colored borders in **g** and connected to the relevant pathways in **f** using colored dashed lines. Functional interaction networks and pathways for the spatial cluster 2-enriched gene network have been illustrated in **h**–**k**.

On examining the GO biological processes [53] enriched in the spatial clusters, using a hypergeometric test (BH-corrected *p* < 0.05), we found that spatial cluster 1 was exclusively enriched for genes involved in the glutamate (Glu) receptor signaling pathway (Fig. 6a). Four Glu receptors were responsible for this enrichment (GRIA3, GRIN2B, GRIK2, and GRM7). Spatial cluster 2 was exclusively enriched for genes involved in dopaminergic (DA) synaptic transmission (Fig. 6a), specifically, two receptors (DRD2 and ADORA2A) and two transporter genes (SLC6A2 and SLC6A4). Although the serotonin (5-HT) receptor signaling pathway was enriched in both the spatial clusters, it showed a higher enrichment in spatial cluster 2 (Fig. 6a). Different subsets of 5-HT receptors contributed to this enrichment, namely, HTR1B, HTR3C, HTR3D and HTR3E for spatial cluster 2 and HTR1A, HTR2A and HTR3B for spatial cluster 1. The enrichment of the spatial clusters for specific synaptic signaling pathways was also validated using PCA (Supplementary Fig. 11 and Supplementary Note 5). Further analysis confirmed that the Glu and DA/5-HT dichotomy was indeed the factor that influenced the separation of the limbic-associated and midbrain-biased spatial clusters 1 and 2 (Supplementary Fig. 12 and Supplementary Note 5). Upon re-inspection of spatial cluster 2, we identified two sub-clusters that showed differential enrichment in 5-HT and DA systems and varied expression correlations in the midbrain raphe nuclei, basal forebrain and basal ganglia (Supplementary Fig. 13 and Supplementary Note 6).

Hence, we concluded that the spatial clusters not only displayed spatial variations but also had associations with distinct signaling pathways. This led us to suspect that the AD genes formed co-expression networks with specific functional affiliations within distinct brain regions. Through a co-expression network analysis, using the Cytoscape Expression Correlation plugin [54], of the AD genes based on their expression profiles in cerebral nuclei, the limbic system and the midbrain, we identified two networks, one comprising 92 genes (Supplementary Fig. 14a) and the other 16 genes (Supplementary Fig. 15a). These networks exhibited pairwise gene correlations of *r* > 0.5, where r is Pearson correlation coefficient. The 92-genes network displayed a statistically significant overlap (*p* = 2.21E-04) exclusively with spatial cluster 1, with ~47% (43 genes) belonging to spatial cluster 1. This network was expressed in some of the structures enriched in spatial cluster 1, such as the caudate nucleus head and tail (Supplementary Fig. 14b). Despite its broader involvement in various biological processes compared to spatial cluster 1, the 92-genes network showed a statistically significant signal for the Glu signaling pathway (Supplementary Fig. 14c). In contrast, all genes in the 16-genes network were associated with spatial cluster 2 (*p* = 1.02E-03). This network exhibited enrichment in midbrain structures, which were also enriched in spatial cluster 2, such as the cuneiform nucleus (Supplementary Fig. 15b). Additionally, in line with the enrichment of the 5-HT signaling pathway in spatial cluster 2, the 16-genes network showed enrichment solely in this pathway (Supplementary Fig. 15c). In summary, we showed that the regions enriched in the 92-genes network and the 16-genes network were subsets of the regions originally identified in spatial cluster 1 and spatial cluster 2, respectively. This confirmed that the genes within these spatial clusters indeed form co-expression networks in specific brain regions. Moreover, the enrichment of Glu and 5-HT pathways in spatial cluster 1 and spatial cluster 2 was also replicated by the two gene networks.

Interestingly, we also observed a large number of biophysical interactions among the proteins encoded by spatial clusters 1 and 2, indicating their potential functional cohesiveness (Supplementary Fig. 16 and Supplementary Note 7). The network of protein-protein interactions (PPIs) or the ‘protein interactome’ can be studied to determine higher-order relationships such as the pathway interactions that exist among disease-associated genes [55–57]. Therefore, we (i) generated the network of the proteins encoded by AD-associated genes using the STRING database [58], (ii) isolated densely connected regions (referred to as ‘sub-networks’ henceforth) in this network potentially involved in specific cellular functions using the Markov Clustering (MCL) algorithm and (iii) retrieved pathway interactions (using the Cytoscape plugin ReactomeFiViz [59]) from these sub-networks in which two proteins participate in the same reaction as components of a protein complex, or as an input, catalyst, activator or inhibitor (see Methods).

The interaction network contained the proteins encoded by 93 AD-associated genes interconnected via 380 functional associations, including those based on gene co-expression (224/380 associations), sequence homology (22/380 associations), shared chromosomal neighborhood (8/380 associations), phylogenetic co-occurrence (2/380) and experimentally determined PPIs (124/380 associations) (Supplementary Fig. 17). We obtained 23 sub-networks after applying MCL (inflation parameter = 3) to this network.

Spatial cluster 1 was significantly enriched for only 2 of these sub-networks, out of which only one was enriched for a specific function, computed based on GO biological processes, namely, Glu receptor signaling pathway (FDR-corrected *p* = 1.61E-05). This sub-network (Fig. 6b) contained 6 genes, which included 4 Glu receptors (GRIA3, GRIK2, GRIN2B, and GRM7) and one Glu transporter (SLC1A1). Additionally, this sub-network showed high expression in the hippocampal formation (Fig. 6b). Spatial cluster 2 was significantly enriched for only 2 sub-networks (Fig. 6c), one of which was enriched for cytokine secretion (*p* = 0.01; the genes responsible include PTPN22, TNF and GRN) and another for 5-HT and melatonin biosynthesis (*p* = 4.65E-03). The 5-HT synthesis sub-network (Fig. 6c) contained 6 genes, which included 2 enzymes responsible for 5-HT production (TPH1 and TPH2) and one 5-HT transporter (SLC6A4). This sub-network was highly expressed in the midbrain and pons regions (Fig. 6c).

We found that 6 proteins in the spatial cluster 1-associated sub-network (Fig. 6d) could be interlinked via 10 functional interactions, by adding 3 ‘linker’ proteins to facilitate network identification (Fig. 6e). Four of these proteins were involved in pathway interactions mediated by 3 ionotropic Glu receptors (NMDA, AMPA and kainite receptors) (Fig. 6f, g and Supplementary Note 8). In the spatial cluster 2-associated sub-network (Fig. 6h), 6 proteins could be interlinked via 14 functional interactions, by adding 7 linker proteins (Fig. 6i). Three of these proteins were involved in metabolic pathways of amine-derived hormones, including two that participate in 5-HT and melatonin biosynthesis (TPH1and TPH2) (Fig. 6j, k and Supplementary Note 8). We noted that the 6 genes in the sub-network were confined to the 5-HT-enriched sub-cluster 2a (Supplementary Fig. 13 and Supplementary Note 6). The transporter genes were co-expressed with the corresponding receptors or synthetic enzymes as expected, supporting the validity of the spatial cluster-enriched sub-networks (Supplementary Fig. 18 and Supplementary Note 9). Three genes interacting with anxiolytic drugs were found in the two sub-networks (Supplementary Fig. 19 and Supplementary Note 10).

We detected two clusters upon the hierarchical clustering of 148 AD-associated genes based on their cell-specificity scores – pre-computed as detailed by Birnbaum et al. [60] and available from the PsychENCODE (Human Brain Evolution) portal (http://www.evolution.psychencode.org/) — in 29 transcriptionally distinct cell types of the human dorsolateral prefrontal cortex (single-nucleus sequencing data) [61]. At the level of cell types, we noted that cluster 1 significantly overlapped with spatial cluster 1 and contained several genes showing high specificity scores in excitatory and inhibitory cells. In contrast, cluster 2 showed a significant overlap with spatial cluster 2 and high specificity scores in astrocytes and microglia (Supplementary Fig. 20). An independent dataset with fewer cell types corroborated these results (Supplementary Fig. 21 and Supplementary Note 11).

Together, this analysis confirmed that the two spatial clusters were associated with distinct behavioral traits, signaling pathways and cell types, and contained functionally cohesive, region-specific and clinically actionable gene networks and pathway interactions, providing a mechanistic basis for their further examination.

### Temporal expression patterns of AD-associated genes

**Fig. 7:**
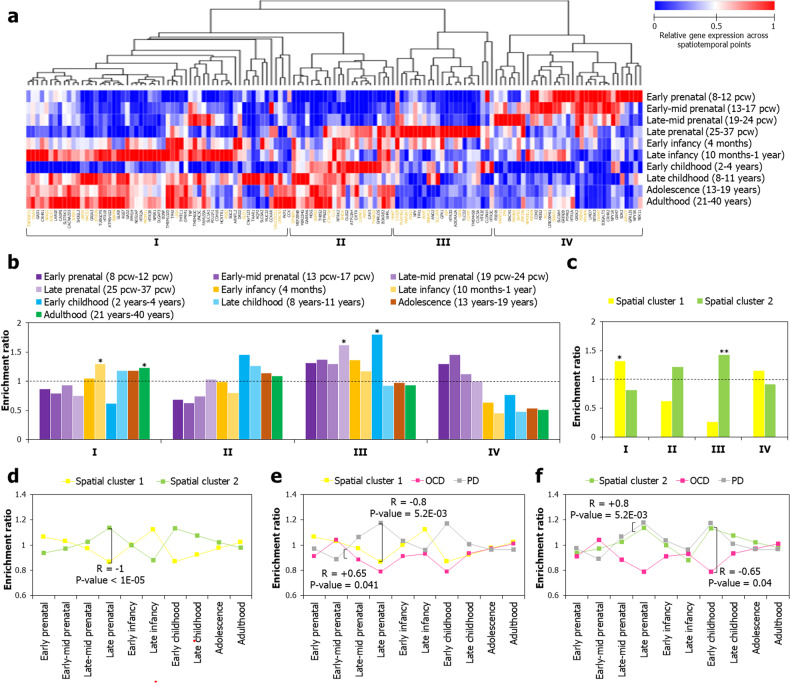
Spatial clusters showed negatively correlated temporal expression patterns. **a** Four temporally distinct clusters (labeled from I-IV on the horizontal axis at the bottom) were isolated from AD-associated genes by performing hierarchical clustering on expression data partitioned into ten developmental stages as shown on the vertical axis. Housekeeping genes have been shown in brown font. Clustering was performed on log_2_-transformed Reads Per Kilobase Million (RPKM) values using the hierarchical clustering method with average linkage. The dendrograms were derived from the clustering analysis based on computation of Pearson correlation coefficients between the data points. The clustered heat map was created using the Morpheus software. pcw: post-conceptional week. **b** The temporal identities of the clusters I-IV were characterized using their enrichment ratios among genes highly expressed in each of the ten developmental stages. **c** The association of the spatial clusters with the temporal clusters was ascertained by computing the enrichment ratios of the genes belonging to the temporal clusters I-IV in spatial cluster 1 and spatial cluster 2. In **b** and **c**, *, **, and *** indicate *p* < 0.5, < 0.01 and < 0.001 after multiple test adjustment using the Benjamini–Hochberg method. The dotted black line indicates the cut-off value for enrichment ratio, i.e., 1. The correlation of the enrichment ratios of **d** spatial cluster 1 with spatial cluster 2, **e** spatial cluster 1 with OCD- and PD-associated genes and **f** spatial cluster 2 with OCD- and PD-associated genes was compared among genes highly expressed in the ten different developmental stages (shown on the horizontal axis). Pearson correlation coefficient (r) was used to examine the similarities and differences in the temporal identities of these groups of genes.

ADs are often characterized as neurodevelopmental disorders since they collectively have earlier onset ages (compared to other neuropsychiatric disorders) [62]. As the age onset varies between the different subtypes, this suggests that temporal regulation of developmental programs may determine the expression of anxiety symptoms at different ages [63]. Furthermore, shared genetic risk factors between AD subtypes and the high co-morbidity of AD subtypes across the lifespan indicate that these developmental programs may be genetically determined [63]. Thus, we hypothesized that the expression patterns of AD-associated genes in specific developmental stages could provide clues on the developmental trajectory of AD symptoms. We examined the expression patterns of 139 AD-associated genes throughout the course of brain development using the developmental transcriptome data available in the BrainSpan Atlas [50]. The data was partitioned into ten developmental stages (Fig. 7a): early, early-mid, late-mid and late prenatal stages, early and late infancy, early and late childhood, and adolescence and adulthood stages (see Methods).

Hierarchical clustering revealed four temporal clusters (I-IV) of AD-associated genes showing distinct temporal expression patterns (Fig. 7a); their regional specificities can be seen in Supplementary Fig. 22. Housekeeping genes (detected in all the tissues at transcripts per million ≥1, as per Human Protein Atlas) constituted 26% of temporal cluster 1 (15 out of a total of 58 genes in the cluster), 35% of II (8/23), 48% of III (11/23) and 39% of IV (13/33). However, their distribution in the temporal clusters was as expected by random chance from hypergeometric distribution (i.e., BH-corrected *p* > 0.05). Temporal cluster I contained 58 genes and was enriched for high expression during late infancy (10 months-1 year; *p* = 0.032) and adulthood (21 years-40 years; *p* = 0.032) phases, whereas temporal cluster III contained 23 genes and was enriched for high expression during the late prenatal stage (25–37 pcw; *p* = 0.03) and early childhood (2–4 years; *p* = 0.03) (Fig. 7b). Temporal cluster 1 showed enrichment for spatial cluster 1 (*p*-value = 0.029) and temporal cluster 2 for spatial cluster 2 (*p* = 6.6E-03) (Fig. 7c). Therefore, genes in temporal clusters I and III intersected with specific developmental stages and spatial clusters (Supplementary Fig. 23) and we identified the top-20 pathways that were enriched among these genes (Supplementary Fig. 24).

We noted that the enrichment ratios of the two spatial clusters among genes highly expressed during specific stages were highly negatively correlated (R = −1; *p* < 1E-05) (Fig. 7d). Further, to determine whether specific genes were prominently expressed in a particular brain region during specific developmental stages, we obtained temporal expression profiles from the BrainSpan Atlas for 24 genes belonging to spatial cluster 1 (Supplementary Fig. 25a). These genes exhibited high expression in hippocampal regions, including CA1-CA4, the dentate gyrus, and the subiculum (based on microarray data). Hippocampal regions were selected due to their consistent association with spatial cluster 1. Across the ten developmental stages, we observed a strong positive correlation between the average expression values of the subset of spatial cluster 1 genes highly expressed in the hippocampus and the average expression values of all spatial cluster 1 genes in all the BrainSpan regions (Supplementary Fig. 25b). From this, we confirmed that the hippocampal spatial cluster 1 genes recapitulated the overarching temporal pattern of spatial cluster 1, characterized by peak expression in late infancy, a rise from late childhood to adulthood, and notable drops during late prenatal and early childhood stages.

To elucidate the temporal patterns of AD subtypes, we examined the enrichment ratios of the genes associated with the four AD subtypes in various developmental stages. Notably, only OCD- and PD-associated genes—which were instrumental in segregating the two spatial clusters (Fig. 5b)—showed significant patterns. The enrichment ratios of OCD-associated genes in specific stages showed a moderate positive correlation with that of spatial cluster 1 (R = +0.65, *p* = 0.041) (Fig. 7e), and the ratios of PD-associated genes showed a strong positive correlation with that of spatial cluster 2 (R = +0.8, *p* = 5.23E-03) (Fig. 7f). Conversely, PD-associated showed a strong negative correlation with spatial cluster 1 (R = −0.8, *p* = 5.23E-03) (Fig. 7e) and OCD-associated genes showed a moderate negative correlation with spatial cluster 2 (R = −0.65, *p* = 0.041) (Fig. 7f). Overall, we concluded that the two spatial clusters have two distinct and negatively correlated temporal identities with twin peaks at specific developmental stages, which were shared with specific AD subtypes.

### Trifurcation of the AD gene set upon inclusion of PTSD-associated genes

Finally, we examined whether the two spatial clusters we obtained with AD-associated genes were also relevant for disorders not classically defined as ADs, but possibly sharing a common background with ADs and having a genetic basis.

In DSM-5, post-traumatic stress disorder (PTSD) was not included in the category of ADs, and was instead classified under the category of trauma- and stressor-related disorders, due to its behavioral phenotypes that were incongruent with the broader AD symptoms [64]. However, recognizing the centrality of AD traits such as fear and avoidance to PTSD development and treatment [65], we identified 141 PTSD-associated genes from the GWAS catalog and DisGeNET (Supplementary Table 2) and re-performed the spatial clustering analyses. Twenty-four of these genes overlapped with our original AD gene set. The expression profiles of 71 of the remaining 117 unique PTSD-associated genes were available for the analysis. Cerebral nuclei, the midbrain, and the limbic system remained the predominant tier 1 structures, whether considering PTSD-associated genes alongside other AD-associated genes or as an independent gene set (Supplementary Table 3). Hierarchical clustering of the spatial profiles of all the AD genes, including those associated with PTSD, identified three spatial clusters A–C (Supplementary Fig. 26a). Spatial cluster A exhibited higher expression in limbic system samples compared to midbrain samples (Supplementary Fig. 26b, f). In contrast, spatial clusters B and C showed higher midbrain (than limbic) expression, albeit in a non-significant manner (Supplementary Fig. 26c, d), with C showing higher midbrain expression compared to B (Supplementary Fig. 26g). The three clusters showed enrichment for specific tier 3 cerebral nuclei (Supplementary Fig. 27a). Spatial cluster A showed exclusive enrichment for limbic structures (Supplementary Fig. 27b), while spatial clusters B and C showed enrichment for different subsets of midbrain structures (Supplementary Fig. 27c).

Notably, spatial cluster A exclusively overlapped with the limbic-associated spatial cluster 1 identified in the original AD gene set, while spatial clusters B and C overlapped with the original midbrain-biased spatial cluster 2, suggesting its division into two clusters with the inclusion of PTSD genes (Supplementary Fig. 28a). Unlike our analyses using the original AD gene set, this division helped us obtain statistically significant enrichments for disorder-specific genes in the clusters, specifically, SAD- and PTSD-associated genes in spatial cluster B (Supplementary Fig. 28b). This spatial cluster also showed enrichment for the central nucleus of the amygdala, a region implicated in the etiology of both disorders (Supplementary Fig. 27a) [66–69].

In summary, by adding genes associated with PTSD, which has a well-established genetic basis supported by GWA studies, to the initial AD gene set, we were able to strikingly separate the original spatial cluster 2 into two clusters (spatial cluster B and spatial cluster C). The regions enriched in these two clusters were distinct subsets of the midbrain structures enriched in spatial cluster 2. Moreover, spatial cluster B displayed clear enrichment signals for PTSD and SAD genes.

## Discussion

Despite the discovery of neural circuits functionally associated with ADs through physiological experiments, whether AD-associated genes show corresponding spatial distribution patterns remains unexplored. Since previous attempts have used genetic data from GWAS of only a single AD subtype [13] and rodent models [14, 15], which have neither the genes nor brain regions to recapitulate human AD [70, 71], it has not been possible to identify the regional specificities associated with ADs within the human brain. Here, we systematically examined the spatial expression patterns of genes identified to be associated with four AD subtypes through GWAS and determined that the cerebral nuclei, midbrain and limbic system regions showed enrichment for AD-associated genes, irrespective of AD subtypes (Tables 1a, b). To the best of our knowledge, our study is the first to demonstrate the preferential bias in the expression patterns of AD-associated genes to the specific structures in these regions previously linked to the anxiety state in physiological studies (Fig. 3a–c). Hence, our study establishes a clear relationship between AD-associated genes identified in GWA and targeted sequencing studies and regional specificities discovered through PET and microstimulation studies.

Furthermore, we uncovered a previously unreported bifurcation among AD-associated genes based on their differential expression in the cerebral nuclei, limbic and midbrain regions, and identified the neural circuits, signaling pathways, cell types and temporal patterns underlying these gene clusters. The AD-associated genes split into the limbic-associated spatial cluster 1 and the midbrain-biased spatial cluster 2, each affiliated with specific cerebral nuclei (Fig. 2a), which was confirmed through three additional methods (Fig. 4) and two independent datasets (Supplementary Figs. 6 and 7). Previous studies have implicated these specific structures enriched in the spatial clusters (Fig. 3) in the regulation of negative decision-making and anxious temperament in rhesus monkeys. Interestingly, these behaviors correspond to state and trait anxiety states in humans.

In macaque studies of trait anxiety using PET, metabolism in the hippocampus, the central nucleus of the amygdala, the bed nucleus of the stria terminalis (or BNST), and periaqueductal gray (or central gray substance) were found to predict individual differences in anxious temperament [9, 10]. Genes in the spatial clusters were enriched for these same regions, exhibiting a clear correspondence with the physiological experiments. The recapitulation of these regions, even when the AD gene set is constrained to include only those supported by evidence from eQTL and differential gene expression studies (Supplementary Fig. 5), strongly indicates the existence of two different sets of AD genes with distinct spatial expression profiles. Specifically, we found that the expression of AD-associated genes was enriched in areas located close to the BNST (septal nuclei), the hippocampal system (parahippocampal gyrus), and periaqueductal gray (raphe nuclei, inferior colliculus, cuneiform nucleus and interstitial nucleus of Cajal). In the studies of state anxiety, microstimulation of multiple brain regions such as the caudal orbital frontal cortex, insula, and subgenual anterior cingulate cortex, and the striatum (caudate nucleus, putamen, and nucleus accumbens) [11, 12] induced pessimistic bias in conflict decision-making. These findings suggest the causal involvement of these regions in state anxiety. These areas send projections to the ventral tegmental area, suggesting that regulating the dopaminergic system could be the key feature of the neural circuits involved in state anxiety [72, 73]. Our analysis showed that the expression of AD-associated genes was enriched in these and the adjacent regions. Altogether, the AD-associated genes converged on specific neural systems linked to AD subtypes (Supplementary Discussion), and regulating multiple aspects of state and trait anxiety, namely, sensitivity to threat stimuli (extended amygdala) [74], threat-related behavioral inhibition (septohippocampal system) [75], reinforcement contingencies [76] and motivational conflicts [77] (the striatum) and stress-induced defensive responses (periaqueductal gray) [78]. Our findings also suggest that the neural systems underlying the spatial clusters were distinct. Hence, these gene clusters, when perturbed, may elicit different behavioral phenotypes and affect distinct signaling pathways, gene networks, and cell types, which we examined through enrichment analyses.

Inspection of PC loadings of the two clusters revealed that at the level of behavioral phenotypes, the separation of spatial cluster 1 and spatial cluster 2 was respectively influenced by OCD and PD genes identified in GWA studies (Fig. 5b). Additionally, the two spatial clusters showed distinct, albeit statistically non-significant, patterns of enrichment ratios for AD subtype-specific genes (Supplementary Fig. 10). However, when we added PTSD genes to our original AD gene set, we could clearly detect statistically significant enrichment signals for PTSD and SAD genes in spatial cluster B, a subset of spatial cluster 2 (Supplementary Fig. 28b). Altogether, these findings suggest that the two spatial clusters were associated with different symptom profiles represented by the AD subtypes (Supplementary Discussion).

We gained some insights into the relationships between the different AD subtypes using co-expression analysis of AD subtype-specific genes in cerebral nuclei, limbic, and midbrain regions (Supplementary Fig. 3b). We found that all AD subtypes displayed moderate correlations, with variations in the direction of correlation. First, PD and SAD genes exhibited moderate positive correlation, possibly reflecting their clinical comorbidity, as seen in situational panic attacks in SAD patients [79]. However, SAD genes were also moderately correlated with OCD genes, possibly underlying shared traits such as interpersonal sensitivity and obsessive doubts [80], despite the lack of a direct clinical correspondence. Second, GAD genes showed moderate negative correlation with OCD genes and SAD genes and moderate positive correlation with PD genes (Supplementary Fig. 3b). These patterns aligned with the enrichment ratios of AD subtype-specific genes in the two spatial clusters (Supplementary Fig. 10a). GAD had a higher enrichment ratio in the midbrain-biased spatial cluster 2, similar to PD, possibly due to their clinical comorbidity, as evidenced by catastrophic cognition-induced panic attacks in GAD [81]. In contrast, OCD genes had a higher enrichment ratio in spatial cluster 1, distinct from the pattern of GAD. These results suggest that the clinical differences between GAD and OCD, rooted in their respective associations with obsessional thoughts and perseverative worry [82], may be linked to anti-correlated regional expression patterns. SAD was mainly enriched in spatial cluster 2, similar to GAD, but lacked any association with spatial cluster 1, establishing a distinct enrichment pattern from GAD. In summary, GAD, OCD, and SAD symptoms likely involve distinct gene groups with unique expression patterns.

Additionally, the two spatial clusters were associated with distinct signaling pathways, suggesting a dichotomy in the neurophysiology of the AD symptoms regulated by them. Spatial cluster 1 was involved in Glu receptor signaling pathway, and spatial cluster 2 in 5-HT and DAergic signaling (Fig. 6a). Supporting the regional (Fig. 3b) and AD subtype specificities (Supplementary Fig. 10a) of spatial cluster 1, Glu signaling is predominantly seen in hippocampal regions and the surrounding medial temporal cortex [83], and Gluergic modulation has been implicated in OCD pathogenesis [84]. On the other hand, 5-HT production is confined to the midbrain raphe nuclei [85] and the ventral tegmental area is a hub for DA production [86], both midbrain areas associated with spatial cluster 2 (Fig. 3c). 5-HT and DA play important roles in AD subtypes affiliated with spatial cluster 2. In PD patients, 5-HT agonists induced anxiogenic responses, whereas selective 5-HT reuptake inhibitors showed therapeutic benefits [87].

Our findings indicate that the spatial clusters contain functionally compact, regionally specific, and druggable gene modules belonging to neuronal signaling pathways. They could operate at the mechanistic level to regulate AD symptoms. We noted that the high interconnectivity of the proteins encoded by the spatial cluster genes (Supplementary Fig. 16) could be correlated with functional cohesiveness, as shown by the network medicine paradigm [88]. This led us to examine a network in which AD-associated genes were interlinked (Supplementary Fig. 17) based on functional associations and physical interactions (of the proteins encoded by AD genes). Notably, we could independently isolate sub-networks enriched with the spatial clusters from this AD gene network. These sub-networks showed the regional and pathway specificities of the spatial clusters (Fig. 6b–k) and contained genes interacting with anxiolytic drugs (Supplementary Fig. 19); additional findings indicate that the druggable AD gene space remains unexplored (see Supplementary Discussion).

Our results suggest that specific cell types were affiliated with the spatial clusters and could be selectively vulnerable to AD-associated gene perturbations. However, further investigations with more neuronal cell types are required to ascertain these findings. Nevertheless, spatial cluster 1 overlapped with a gene cluster enriched for excitatory cell markers (Supplementary Fig. 20), which was expected based on the association of Glu signaling with spatial cluster 1, and spatial cluster 2 with astrocytes and microglia, cell types that secrete inflammatory cytokines (Supplementary Fig. 20). The latter corroborated the enrichment of cytokine secretion, a process influencing the severity of anxiety symptoms [89], in spatial cluster 2-enriched sub-network (Fig. 6c), warranting further investigations on the role of inflammatory processes in AD etiology.

Our genetic data integrated across the AD subtypes did not corroborate the onset ages reported in other studies (14.5–15.5 years) [90]. Instead, the enrichment ratios of the four temporally distinct clusters of AD-associated genes (Fig. 7a) either peaked progressively across several stages (temporal clusters II and IV) or peaked in two specific stages (temporal clusters I and III) (Fig. 7b). These patterns corresponded respectively to waterfall and twin-peak modes, previously reported for genes differentially expressed during neocortical development [91]. Interestingly, the spatial clusters showed enrichment for temporal clusters with twin-peaks (Fig. 7c), indicating that AD mechanisms were likely to be regulated in two critical life stages, perhaps as proposed in the dual hit hypothesis for schizophrenia [92]. AD-associated mutations may perturb the temporal pattern of genes whose expression peak in specific developmental stages (e.g., late infancy in spatial cluster 1 and late prenatal stage in spatial cluster 2). This, in turn, could affect the developmental fates of the signaling systems and neural circuitry regulated by these genes in later stages (e.g., adulthood in spatial cluster 1 and early childhood in spatial cluster 2). However, a more complex framework is needed to explain AD pathogenesis, since regions critical to ADs such as the amygdala and the hippocampus retain their neuroplasticity and could be perturbed even in adulthood [93, 94].

The two spatial clusters, perhaps, regulate the development of the associated AD phenotypes in distinct time windows, as indicated by the negative correlation of their enrichment ratios in different life stages (Fig. 7d). The genetic architectures of OCD and PD could determine their onset and progression, likely regulated by spatial cluster 1 (Fig. 7e) and spatial cluster 2 genes (Fig. 7f) respectively (with which they show positive correlation). Therefore, critical junctures in development could be exploited for therapeutic interventions.

Altogether, we comprehensively characterized the functional underpinnings of the spatial clusters. However, our study has several limitations (Supplementary Discussion), which should be addressed in future works. In sum, we identified two spatially, functionally and temporally distinct clusters of AD-associated genes underlying distinct neural systems operating in the cerebral nuclei, limbic and midbrain regions. These gene clusters will likely guide future studies on the etiological aspects of ADs.

## Methods

### Compilation of AD-associated genes

Two-hundred forty genes, including 26 genes associated with GAD, 9 with SAD, 92 with PD and 147 with OCD, were collected from the GWAS catalog [25] and the DisGeNET database (Supplementary Fig. 1 and Supplementary Table 1) [26]. The genes compiled from the GWAS catalog were found within 50 kb upstream or downstream of the AD subtype-associated SNPs. The genes deposited in the GWAS catalog, GWAS DB [27] and the BEFREE database [28] were compiled from the DisGeNET database (GDA was set at ≥0.01 to ensure that at least one publication has linked the gene in question with the disease). Note that association of a gene with a disease does not imply causality in most cases, and may only indicate an association with disease susceptibility or an endophenotype. A hypergeometric test was used to calculate the statistical significance of the pairwise overlaps between the gene sets associated with specific AD subtypes.

### Organ systems enrichment analysis

The enrichment of the AD-associated genes for genes expressed in specific tissues was computed using RNA-sequencing data from GTEx [29] and Human Protein Atlas [30]. From GTEx, the genes showing high/moderate expression (TPM ≥ 9) in the RNA-sequencing data of 52 postnatal human tissues were included, provided that they were not housekeeping genes, i.e., genes detected in all the tissues with TPM ≥ 1. From the Human Protein Atlas, genes showing tissue-enriched/tissue-enhanced/group-enriched expression in 35 tissues were considered. Tissue-enriched genes showed at least 5-folds higher mRNA levels in a particular tissue compared to all the other tissues. Group-enriched genes showed at least 5-folds higher mRNA levels in a group of 2–7 tissues and tissue-enhanced genes showed at least 5-folds higher mRNA levels in a particular tissue compared to average levels in all tissues. The enrichment of the AD-associated genes in various tissues were examined using a hypergeometric test, and the BH method for multiple hypotheses correction was applied to the obtained *p*-values. The threshold for statistical significance was set at *p* < 0.05.

### Brain region enrichment analysis

We examined the spatial expression patterns of the 240 AD-associated genes using the adult microarray data available in the Allen Brain Atlas [24]. Normalized microarray data was available for 29,130 genes from the brains of six healthy donors (IDs 9861, 10021, 12876, 14380, 15496 and 15697), each dissected into 363–946 samples—yielding a total of 3702 samples—from 13 areas in the brain, namely, cerebellum, cerebral nuclei, diencephalon, frontal lobe, insular cortex, limbic lobe, medulla oblongata, midbrain, occipital lobe, parietal lobe, pons, temporal lobe and white matter. Note that (a) the probes matching multiple genes were excluded, (b) if the same gene has been detected by multiple probes, the expression levels were averaged across the probes, and (c) 232 brain regions (414 when the samples from the left and right hemispheres are treated separately) were clustered into 13 brain areas (and the ventricles) based on the structured vocabulary used in Allen Brain Atlas to classify them. For the purpose of our study, we labeled these 13 brain areas as ‘tier 1 structures’ and the 232 sampled areas as ‘tier 3 structures’, and added an intermediary ‘tier 2’ to cluster the tier 3 structures into major subdivisions within the tier 1 structures. We sought to pinpoint the tier 3 structures that showed high expression of the AD-associated genes, and then map these structures to their parent tier 1 structures. We compiled a list of genes highly expressed in each of the 232 tier 3 structures relative to others, as described by Rouillard et al. [40], in the form of a gene matrix transposed (GMT) file. This GMT file was uploaded as a custom database to WebGestalt [95] for performing an over-representation analysis (ORA) with the AD-associated genes. Statistical significance of the overlaps between the list of AD-associated genes and the genes highly expressed in a specific tier 3 structure was computed based on hypergeometric distribution. In this method, *p*-value is computed from the probability of k successes in n draws (without replacement) from a finite population of size N containing exactly K objects with an interesting feature.

$$P\left(X=k\right)=\frac{{{K}\choose{k}}{{N-K}\choose{n-k}}}{{{N}\choose{n}}}$$

Population size N = Number of genes expressed in the human brain

Number of successes in the population K = Number of genes highly expressed in a specific tier 3 structure

Sample size n = Number of AD-associated genes

Number of successes in the sample k = K ∩ n

The *p*-values derived from ORA were corrected for multiple hypothesis using the BH method. In this method, the hypergeometric *p*-values are sorted from small to large, and multiplied by the total number of tests and then divided by its rank order. *P* < 0.05 after BH correction was considered to be statistically significant.

### Hierarchical clustering of spatial expression profiles and cell-specificity scores

To identify clusters of AD-associated genes having distinct spatial expression profiles in the cerebral nuclei, limbic and midbrain regions, we examined the adult microarray data in Allen Brain Atlas [24]. Log_2_ transformation was performed on all the probe intensity values to reduce the influence of extreme values. Expression data for 139 out of the 240 AD-associated genes were available in the dataset, i.e., for 17 out of the 26 GAD-associated genes, 7 out of the 9 SAD-associated genes, 60 out of the 92 PD-associated genes and 79 out of the 147 OCD-associated genes. Hierarchical clustering was performed on a data matrix containing the log_2_(probe intensity) values of 139 AD-associated genes in 374 cerebral nuclei, 397 limbic system and 182 midbrain samples using the Morpheus software [42]. Pairwise distances in the data matrix were calculated using Pearson correlation and closely linked clusters were identified using the average linkage method. The enrichment for tier 3 structures in the clusters of AD-associated genes derived from this analysis was examined using a hypergeometric test. The obtained *p*-values were corrected for multiple hypotheses using the BH method and the *p*-value threshold after correction was set at <0.05. The Mann–Whitney *U* test was used to compare the average probe intensities of the two spatial clusters across cerebral nuclei, limbic and midbrain regions. We additionally sought to examine the grouping of AD-associated genes based on their affiliation with various brain cell types. Hierarchical clustering was performed using the cell type specificity scores of 139 AD-associated genes in 29 distinct cell types of the human dorsolateral prefrontal cortex available in PsychENCODE [61]. Pearson correlation was used as the distance metric and the average linkage method was used to identify gene clusters.

### PCA, t-SNE and k-means clustering of spatial expression profiles

Following the established approach to reduce the influence of extreme values on the PCs [96], the probe intensity values were log_2_-transformed and assembled into a data matrix containing 139 AD-associated genes (rows), and 374 cerebral nuclei, 397 limbic system and 182 midbrain samples (columns), which was used as input for PCA, performed using the web-based tool Clustvis [97]. The data matrix was pre-processed to include only those rows and columns that contained less than 70% missing values. The log_2_(probe intensity) values in the matrix were centered using the unit variance scaling method, in which the values are divided by standard deviation so that each row or column has a variance of one; this ensures that they assume equal importance while finding the components. The method called singular value decomposition (SVD) with imputation was used to extract principal components. In this method, missing values are predicted and iteratively filled using neighboring values during SVD computation, until the estimates of missing values converge. The number of PCs computed was equal to the number of column dimensions in the data matrix, i.e., the number of brain samples in our case. PCA essentially transformed our original variables (log_2_probe intensity) into uncorrelated variables or PCs. These PCs were ranked in the descending order of the percentage of total variance explained by them. The positions of each observation (denoting a specific gene) in the PC plot are called component scores and are calculated as linear combinations of the original variables (expression of the gene in specific cerebral nuclei/midbrain/limbic samples) and the corresponding weights a_ij_ (also known as loading values). For example, the score for the rth sample on the kth principal component is calculated as

$${Y}_{{rk}}={a}_{1k}{x}_{r1}+{a}_{2k}{x}_{r2}+\ldots {+\,a}_{{pk}}{x}_{{rp}}$$

The importance of each sample is reflected by the magnitude of their corresponding loading values on the principal components (PC1 and PC2), and these values were used to identify the samples that were most influential in separating the AD gene clusters. The t-SNE plot was constructed based on the expression of 139 AD-associated genes in cerebral nuclei, limbic system and midbrain samples using the following parameters: distance metric = 1—Pearson Correlation Coefficient, epsilon = 10 and perplexity = 30. K-means clustering was performed on the same dataset using the following parameters: distance metric = 1—Pearson Correlation Coefficient, number of clusters = 2 and maximum number of iterations = 1000.

### Enrichment of GWAS traits, GO biological process and cell type markers in spatial clusters

The enrichment of the spatial clusters for the genes associated with different GWAS traits and specific biological processes (Gene Ontology [98]) was computed using WebGestalt [95]. WebGestalt computes the distribution of genes belonging to a particular functional category in the input list and compares it with the background distribution of genes belonging to this functional category among all the genes that belong to any functional category in the database selected by the user. Statistical significance of functional category enrichment is computed using a hypergeometric test and corrected using the BH method for multiple test adjustment. Annotations with BH-corrected *p* < 0.05 were considered significant. The –log_10_(*p*-values) obtained from the enrichment analysis with GWAS traits were used as inputs for PCA (as seen in previous studies [99, 100], which was performed to identify the traits that were influential in separating the two spatial clusters. To examine the enrichment of the spatial clusters in specific cell types, we compiled the lists of marker genes that are specifically expressed in neuronal and non-neuronal cell populations of the prefrontal cortex from a study by Lake et al. [101], namely, 79 genes in astrocytes, 157 genes in excitatory cells, 303 genes in inhibitory cells, 44 genes in microglial cells, 103 genes in oligodendrocytes and 52 genes in oligodendrocyte precursor cells (OPCs). Only those genes with log_2_(fold change) ≥1 in a given cell type compared to all the other cell types were considered to be cell-specific.

### Network analysis of AD genes

STRING is a repository of known and computationally predicted ‘functional associations’ defined as productive functional relationships between two proteins that contribute to the same biological process [58]. These associations are (a) compiled from primary repositories such as DIP, BioGRID, HPRD, IntAct, MINT and PDB that contain experimentally validated interactions, (b) inferred from pathway information available in expert-curated databases (KEGG) and statistical/semantic connections of proteins available in biomedical literature (MEDLINE), and (c) predicted based on co-expression patterns, genomic information and interactions known in multiple organisms. We first used the STRING database to compare the number of edges (denoting functional associations) interconnecting the genes in each of the spatial clusters with the number of edges in a randomly selected gene set of the same size and degree distribution. Then, we used STRING to construct a functional network of AD-associated genes containing 93 AD genes and 380 functional associations among them. The majority of the functional associations were based on gene co-expression (59%) and experimentally determined PPIs (33%), and the less prevalent ones were based on sequence homology (6%), shared chromosomal neighborhood (2%) and phylogenetic co-occurrence (0.5%). STRING uses the unsupervised algorithm MCL to generate a weighted correlation network based on evidence scores of the functional associations between the genes. The structural organization of the network is then identified by simulating stochastic flows in it. In order to retrieve a substantial number of densely interconnected sub-networks of AD genes, we applied MCL to the AD gene network with the inflation parameter (which influences the granularity of the sub-networks) set at 3. We obtained 23 densely interconnected sub-networks, out of which 8 had 3 or more genes. ReactomeFIViz, a Cytoscape plugin, was used to extract the known pathway interactions between the genes in the spatial-enriched sub-networks extracted using MCL [59]. Cytoscape was used to visualize the networks [54].

### Hierarchical clustering of temporal expression profiles

To identify gene clusters with distinct temporal expression profiles from the AD-associated genes, we examined the developmental transcriptome RNA-sequencing data available in the BrainSpan Atlas [50]. It contained a total of 524 samples from 31 temporal points spanning ten developmental stages: early prenatal (8–12 pcw): 75 samples, early mid-prenatal (13–17 pcw): 97 samples, late-mid prenatal (19–24 pcw): 43 samples, late prenatal (25–37 pcw): 22 samples, early infancy (4 mos): 33 samples, late infancy (10 mos-1 yr): 26 samples, early childhood (2–4 yrs): 44 samples, late childhood (8–11 yrs): 41 samples, adolescence (13–19 yrs): 50 samples and adulthood (21–40 yrs): 93 samples. The sample sizes, when used with the BH method for FDR correction, are adequate to minimize false positives. This method effectively controls FDR, which represents the expected proportion of false positives among all positives that reject the null hypothesis. The BH method also produces a stronger correlation between raw and FDR-adjusted *p*-values compared to other multiple hypotheses correction methods, indicating its higher reliability [102].

Average RPKM values were calculated for multiple brain samples dissected during each of the ten developmental stages. Log_2_ transformation was performed on all the average RPKM values to reduce the influence of extreme values. Note that unexpressed genes showing RPKM = 0 were treated as n/a. The temporal profiles of 139 AD-associated genes across the temporal points partitioned into ten developmental stages were processed into a data matrix of genes versus temporal points. Hierarchical clustering was performed on log_2_RPKM values in this matrix using the Morpheus software [42]. Pairwise distances in the data matrix were calculated using Pearson correlation and closely linked clusters were identified using the average linkage method. We compiled lists of genes showing relatively higher expression in specific developmental stages compared to others (computations described in Rouillard et al. [40]), and checked their enrichment in each of the clusters derived from this analysis to characterize their temporal profiles. We used Pearson correlation (R) to examine the correlation of the enrichment ratios of the spatial clusters and OCD-/PD-associated genes in a pairwise manner.

### Supplementary information

Supplementary Information

Supplementary Data Files

## Data Availability

The AD-associated genes analyzed in this study have been made available as Supplementary Table 1.
