# Supplementary material for: Spatiotemporal expression patterns of anxiety disorder-associated genes
Source: Transl Psychiatry. 2023 Dec 13;13:385. doi: 10.1038/s41398-023-02693-y (PMC10719387; doi:10.1038/s41398-023-02693-y)
Supplement: Supplementary file 1 — Supplementary Information [file 41398_2023_2693_MOESM1_ESM.docx]

## Supplementary Information

Spatiotemporal expression patterns of anxiety disorder-associated genes

This file contains:

- Supplementary Notes 1 to 11
- Supplementary Discussion
- Supplementary Figures 1 to 30
- Supplementary Tables 1 to 3
- Supplementary References 1 to 24

**Supplementary Note 1**

**AD genetic architecture captured by our study**

We attempted to capture the full spectrum of AD genetic architecture (as outlined in ref^1^ and applicable for any complex disorder) to identify AD-specific regions in our study. Firstly, we included AD-associated variants at levels ‘suggestive’ of genome-wide significance (p-value < 1E-05), in addition to those reaching genome-wide significance (p-value < 1E-08), in our study. The former set of variants included those of moderate penetrance, active in specific environmental contexts (e.g. in tissues and cell types, and activated in response to stressful life events), or regulating disease mechanisms in short and specific time periods.^1^ On the contrary, the latter set captured highly penetrant variants, which were active in disease mechanisms irrespective of changing environmental contexts and perhaps regulated them over a long period of time.^1^ Secondly, we included genes examined in targeted sequencing studies, which were selected based on their roles in anxiolytic pharmacology and evidence from animal models of AD pathophysiology.^2^ This allowed us to tap into areas of AD genetic architecture that contained key driver genes coding for druggable proteins and harbouring highly penetrant variants.

**Supplementary Note 2**

**Selective expression of AD genes in brain tissue**

We showed that AD-associated genes were selectively expressed in the nervous system (compared to other organ systems) using a realistic dataset integrating AD genetic data across multiple studies (**Supplementary Fig. 2**). Enrichment for tissues unrelated to AD etiology was detected in analyses that included genetic data from a single AD GWA study,^3^ possibly due to the limited number of tested loci. Adrenal cortex was additionally detected as an enriched organ among AD-associated genes (**Supplementary Fig. 2b**), supporting the abnormal activity of the hypothalamic pituitary adrenal axis noted in GAD, SAD and OCD patients.^4^

**Supplementary Note 3**

**Regions influencing the separation of spatial cluster 1 and spatial cluster 2 in PCA**

The position of each observation (denoting a specific gene) in the plot shown in **Fig. 4a** are called component scores and are calculated as linear combinations of the original variables (expression of the gene in specific cerebral nuclei/midbrain/limbic samples) and the corresponding loading values. The importance of each sample is reflected by the magnitude of their corresponding loading values on the principal components (PC1 and PC2). Therefore, in order to pinpoint the specific samples that strongly contributed to the grouping patterns of the genes as seen in the score plot, we examined their loadings on PC1 and PC2 (**Supplementary Fig. 8a-c**).^5^ Samples from different cerebral nuclei loaded positively on both quadrants I (27.5% of cerebral nuclei samples) and IV (72.5% of cerebral nuclei samples), indicating their influence on both the limbic-associated spatial cluster 1 and the midbrain-biased spatial cluster 2 (**Supplementary Fig. 8a**). Limbic samples selectively influenced the grouping of genes in quadrant 1 that was enriched with spatial cluster 1 genes. Specifically, the loading values of 98.5% of the limbic samples were restricted to quadrant I in the PC loading plot, indicating their strong and exclusive influence on bifurcating spatial cluster 1 from spatial cluster 2 (**Supplementary Fig. 8b**). The loading values of 91.2% of the midbrain samples were restricted to quadrant IV enriched with spatial cluster 2 genes (**Supplementary Fig. 8c**). Overall, this supported the association of limbic samples with spatial cluster 1, midbrain samples with spatial cluster 2 and specific cerebral nuclei samples with both spatial clusters 1 and 2. The midbrain samples that were restricted to quadrant IV loaded positively on the 13 spatial cluster 2 genes present in quadrant IV and negatively on the 20 spatial cluster 2 genes in quadrant III (**Fig. 4a**). Corroborating this, we found that quadrant III spatial cluster 2 genes showed lower expression than quadrant IV spatial cluster 2 genes in midbrain areas (**Supplementary Fig. 29a**). ~54% and ~29% of the spatial cluster 1 genes were found in quadrants I and II; these genes did not show similar expression variations across the limbic samples (**Supplementary Fig. 29b**).

**Supplementary Note 4**

**Recapitulation of the regional specificities of spatial clusters by PCA**

The tier 3 regional specificities shown by both the spatial clusters (**Fig. 3a-c**) were recapitulated in the enrichments shown by quadrants I, III and IV (**Supplementary Fig. 9a-c**). Six out of the seven limbic regions enriched in spatial cluster 1 (**Fig. 3b**) were also enriched in quadrant I (**Supplementary Fig. 9b**). Seven out of the ten midbrain regions enriched in spatial cluster 2 (**Fig. 3c**) were also enriched in quadrant III (**Supplementary Fig. 9c**); one of these regions (inferior colliculus) showed an enrichment of higher statistical significance in quadrant IV than quadrant III, suggesting a region-level distinction between the subsets of spatial cluster 2 genes enriched in the specific quadrants. The enrichment of the claustrum and central nucleus (tier 3 cerebral nuclei) in spatial cluster 1 and spatial cluster 2 (**Fig. 3a**) was replicated in quadrants I and quadrants III (**Supplementary Fig. 9a**). In **Fig. 3a**, septal nuclei, putamen, nucleus accumbens, the head of caudate nucleus, body of caudate nucleus and bed nucleus of stria terminalis (BNST) were observed to be exclusively or more statistically significantly enriched in spatial cluster 2 than in spatial cluster 1. However, PCA showed that there were exclusively enriched in spatial cluster 2 quadrant IV (**Supplementary Fig. 9a**), further supporting the region-level distinction (within the midbrain) of this quadrant from spatial cluster 2 quadrant III. Although the tail of caudate nucleus was earlier found to be more enriched in spatial cluster 1 (**Fig. 3a**), in **Supplementary Fig. 9a**, genes highly expressed in this region seemed to localize to quadrant III (containing spatial cluster 2 genes). This flip in affinities seemed to have been prompted by the segregation of spatial cluster 2 genes into quadrants III and IV and the significant agglomeration of genes expressed in the tail of caudate nucleus in quadrant III. Additionally, the statistical significance of enrichment of this region in quadrants I and IV was fairly equal and lower compared to quadrant III, suggesting its influence in separating out spatial cluster 2 genes and spatial cluster 1 genes along PC1, which captures ~90% of the variance seen in the spatial expression patterns of the AD-associated genes.

**Supplementary Note 5**

**Recapitulation of the synaptic signaling pathway enrichments of spatial clusters by PCA**

Additionally, we had shown that the spatial clusters were differentially enriched for specific synaptic signalling pathways. The exclusive enrichment for the glutamate receptor signalling pathway (Glu) shown by spatial cluster 1 was recapitulated in quadrant I (**Supplementary Fig. 11**). Similarly, the exclusive enrichment for the dopamine receptor signalling pathway (DA) shown by spatial cluster 2 was recapitulated in quadrants II and III (**Supplementary Fig. 11**). The serotonin receptor signalling pathway (Ser) was found to be enriched in quadrant III, recapitulating its affiliation with spatial cluster 2 (**Supplementary Fig. 11**). Next, we sought to confirm whether the Glu/DA-Ser dichotomy was indeed the factor that influenced the separation of the limbic-associated/midbrain-biased spatial clusters. For this, we performed PCA with a matrix containing genes (rows) × brain samples (columns) and observed the separation of the limbic and midbrain samples across PC1 in the PC score plot (**Supplementary Fig. 12a,b**); PC1 and PC2 explained 16% and 12.4% of the expression variance respectively. The position of a specific sample in the PC score plot is computed as a linear combination of the expression values of the genes (in the specific sample) and their corresponding weights or loading values on the PCs that satisfy the criteria detailed by Holland.^5^ In order to pinpoint the genes that strongly influenced the grouping of the limbic samples, we calculated the Euclidean distance between the component scores of the limbic samples and the gene loading values, and extracted the top 20% genes (i.e. top-27 genes out of 139 AD-associated genes) that were relatively more ‘closer’ to the limbic samples (in terms of Euclidean distance) (**Supplementary Fig. 12c**). This procedure was repeated with the midbrain samples (**Supplementary Fig. 12c**). We found that the top 20% genes influencing the grouping of the limbic samples were enriched for the Glu receptor signalling pathway (p-value = 8.79E-03), with 26 out of these 27 genes belonging to spatial cluster 1. On the other hand, the top 20% genes influential in the grouping of midbrain samples were enriched for DAergic synaptic transmission (p-value = 2.62E-03) and Ser receptor signalling pathway (p-value = 2.84E-04), with all of these genes belonging to spatial cluster 2.

**Supplementary Note 6**

**Sub-clusters within spatial cluster 2**

We identified two sub-clusters within spatial cluster 2, namely, sub-clusters 2a and 2b. The enrichment for serotonin receptor signaling pathway (p-value = 5.27E-04) was restricted to sub-cluster 2a and dopamine receptor signaling pathway (p-value = 0.011) to sub-cluster 2b. The sub-clusters also showed different correlated expression patterns in the midbrain raphe nuclei (MRN), basal forebrain (BF) and basal ganglia (BG). The expression of spatial cluster 2a was strongly correlated in BF and BG as shown in **Supplementary Fig. 13a**, whereas the expression of spatial cluster 2b genes was strongly correlated in all the three structures, i.e. BF, BG and MRN as shown in **Supplementary Fig. 13d-f**.

**Supplementary Note 7**

**Interconnectivity of spatial cluster genes in the interactome**

We hypothesized that a higher number of interactions between the proteins encoded by the spatial cluster genes could suggest that they are biologically or functionally cohesive.^6^ Corroborating this, we found that the proteins encoded by the genes in spatial cluster 1 were connected through 65 edges, which was 6.5 times higher than the number of interactions expected to be found between a randomly selected set of proteins of the same size and degree distribution (p-value < 1E-16) (see **Methods**) (**Supplementary Fig. 16a**). Similarly, proteins encoded by the genes in spatial cluster 2 were connected through 149 edges, which was 4.8 times higher than the expected number of associations (p-value < 1E-16) (**Supplementary Fig. 16b**).

**Supplementary Note 8**

**Functional interactions in spatial cluster-associated sub-networks**

In the network enriched for spatial cluster 1 (**Fig. 6d**), we found 10 functional interactions that interconnected 6 proteins encoded by the genes with the help of 3 ‘linker’ proteins that were not included in the network (**Fig. 6e**). Four of these proteins were involved in pathway interactions mediated by 3 ionotropic Glu receptors (NMDA, AMPA and kainite receptors) (**Fig. 6f**). Co-complex interactions between the scaffolding protein DLG1 and GRIN2B, and the reciprocal activating interactions of GRIN2B and GRIA3, and GRIA3 and DLG1, were involved in NMDA receptor activation by Glu and postsynaptic events (**Fig. 6g**). Co-complex interactions between DLG1 and GRIK2 were involved in kainate receptor activation by Glu (**Fig. 6g**). Reciprocal activations between GRIA3 and DLG1 were involved in AMPA receptor trafficking, kainite receptor activation by Glu and synaptic plasticity (**Fig. 6g**). In the network enriched for spatial cluster 2 (**Fig. 6h**), we found 14 functional interactions that interconnected 6 proteins with the help of 7 linker proteins (**Fig. 6i**). Three of these proteins were involved in metabolic pathways of amine-derived hormones, including two that participate in 5-HT and melatonin biosynthesis (TPH1and TPH2) (**Fig. 6j, k**).

**Supplementary Note 9**

**Co-expression of transporters, receptors and synthetic enzymes in spatial cluster-enriched sub-networks**

On assessing the 12 genes present in both the sub-networks (**Fig. 6b, c**), we found that the expression of 5 out of the 7 receptor-encoding genes was localized to cerebral nuclei and limbic structures, with all 5 of them belonging to spatial cluster 1 (**Supplementary Fig. 18**). The three SLC transporter genes showed co-expression with specific receptors or synthetic enzymes in specific areas: SLC1A1 (Glu transporter) co-expressed with GRM7 (Glu receptor) and SLC6A2 (norepinephrine transporter) co-expressed with TAAR1 (trace amine associated receptor 1) respectively, and SLC6A4 (serotonin transporter) co-expressed with TPH1 and TPH2 (serotonin synthetic enzymes). Two out of three SLC transporters were found in spatial cluster 2. Both the serotonin synthetic enzymes – TPH1 and TPH2 – showed co-expression along with SLC6A4 in the midbrain, specifically in the raphe nuclei.

**Supplementary Note 10**

**Targets of anxiolytic drugs in spatial cluster-enriched sub-networks**

We examined the druggability of the gene sub-networks by intersecting the list of 983 drugs targeting ~26% (63 genes) of AD-associated genes (compiled using the drug-gene interaction database^7^) with the list of 70 drugs exhibiting anxiolytic properties (compiled from the Drug Bank database^8^). We identified 29 drugs interacting with 12 AD-associated genes (**Supplementary Fig. 19a**), out of which 3 genes (HTR1A, SLC6A2 and SLC6A4) were found in the spatial cluster-enriched sub-networks, indicating their clinical relevance. The 12 AD-associated genes were not significantly enriched in any brain region. Nevertheless, they bifurcated into two clusters based on their regional specificities in the cerebral nuclei, limbic and midbrain regions (**Supplementary Fig. 19b**). Collectively, they showed enrichment for monoamine transport (**Supplementary Fig. 19c**). However, only 9 drugs collectively targeted the 5 genes involved in monoamine transport (DRD2, HTR1A, HTR2A, SLC6A2 and SLC6A4), whereas 17 drugs targeted GABRA6 involved in GABAergic synaptic transmission (**Supplementary Fig. 19c**).

**Supplementary Note 11**

**Cell-type specificity of spatial clusters**

We examined the enrichment of the spatial clusters among marker genes that are specifically expressed in human neuronal and non-neuronal cell populations. Genes with log_2_(fold change) ≥ 1in a given cell type (compared to all the other cell types) were considered to be cell-specific marker genes; 79 astrocyte, 157 excitatory cell, 303 inhibitory cell, 44 microglial cell, 103 oligodendrocyte and 52 oligodendrocyte precursor cell marker genes were compiled in this manner. Excitatory cell and oligodendrocyte marker genes were enriched by ~2 folds in spatial cluster 1 and spatial cluster 2 respectively (**Supplementary Fig. 21**). Three excitatory cell marker genes (CADPS, CCK and HTR2A) present in spatial cluster 1 and three oligodendrocyte marker genes (MOG, PDE4B and UNC5C) present in spatial cluster 2 were responsible for these enrichments. Although inhibitory cell marker genes were enriched in both the spatial clusters, they showed higher statistical significance in spatial cluster 1 (**Supplementary Fig. 21**). CCK, GRIK2, NPY and TMEM132D belonging to spatial cluster 1 and GAD1, GAD2 and SGCZ belonging to spatial cluster 2 were inhibitory cell marker genes.

**Supplementary Discussion**

**Neural circuits underlying the spatial clusters**

Spatial cluster 1 seemed to underlie a neural system involving the claustrum, the head and tail of the caudate nucleus and the hippocampal formation (**Fig. 3d**). In line with this, medial tracts arising from the claustrum innervate the caudate nucleus and hippocampal regions.^9, 10^ Additionally, the hippocampus plays a critical role in the pathophysiology of OCD,^11^ a subtype that is overrepresented in spatial cluster 1 (**Supplementary Fig. 10a**). On the other hand, spatial cluster 2 genes seemed to be associated with a neural system involving the majority of the basal ganglia (body of caudate nucleus, putamen and nucleus accumbens), the basal forebrain (septal nuclei, bed nucleus of the stria terminalis or BNST and substantia innominata), central nucleus of the amygdala and ten midbrain regions, including the raphe nuclei (**Fig. 3e**). Neural circuits involving the regions enriched in spatial cluster 2 have been linked to social avoidance, a key trait in SAD (AD subtype overrepresented in spatial cluster 2; **Supplementary Fig. 10a**), for e.g. the 5-HT dorsal raphe nuclei pathway innervating the basal amygdala,^12^ which sends unidirectional projections to the central nucleus of the amygdala.^13^ Both the dorsal and median raphe nuclei innervate basal forebrain structures.^14, 15^ The former supply serotonergic innervation to the basal ganglia,^16^ and BNST neurons project directly to serotonergic neurons in the dorsal raphe nuclei.^17^

**Symptom profiles associated with the spatial clusters**

On examining the PC loadings of the two clusters we found that the separation of spatial cluster 1 and spatial cluster 2 was respectively influenced by OCD and PD genes identified in GWA studies (**Fig. 5b**). Therefore, the two gene clusters could be associated with different symptom profiles, i.e. obsessions and compulsions (spatial cluster 1) and spontaneous panic attacks (spatial cluster 2), a dichotomy associated with cued versus uncued triggers,^18^ and used in AD diagnosis.^19^ Restless legs syndrome was interestingly identified as a factor influencing spatial cluster 2 (**Fig. 5b**), perhaps due to its strong association with PD risk in patients.^20^ Major depressive disorder was identified as a factor influencing spatial cluster 1 (**Fig. 5b**), possibly to its strong genetic correlation with OCD.^21^

Interestingly, PCA distinguished OCD as a categorical disorder (diagnosed by a clinician using DSM or other clinical criteria) from obsessive-compulsive (OC) symptoms scored as continuous traits (e.g. in Padua inventory); they respectively influenced spatial cluster 1 and spatial cluster 2 (**Fig. 5b**). Note that the genes associated with OC symptoms are only a small subset of the genes associated with OCD as a categorical disorder (**Supplementary Fig. 30**). This limits the scope of drawing broad conclusions pertaining to OCD from this specific subset. Of the 25 genes associated with OC symptoms in the GWAS catalog, all 18 that overlap with our AD gene set are indeed associated with OCD considered as a categorical disorder. Out of the 12 genes with available expression profiles, 4 are in spatial cluster 1, and 8 are in spatial cluster 2. These differential overlaps resulted in varying levels of statistical significance within the spatial clusters, with spatial cluster 2 showing a higher p-value for genes associated with OC symptoms. This pattern may suggest a closer association of OC symptoms with PD, GAD, and SAD, which exhibit higher enrichment ratios in spatial cluster 2, than with OCD, which has a higher enrichment ratio in spatial cluster 1. However, it is important to note that the AD subtype characterizations of the spatial clusters lack statistical significance (**Supplementary Fig. 10a**), limiting the validity of conclusions drawn about OC symptoms based on these characterizations. A more plausible explanation could be that genes associated with OC symptoms are a small subset of OCD (categorical disorder) genes. The genes associated with OC symptoms could be split into spatial cluster 1 and spatial cluster 2, and those in spatial cluster 1 likely underlie traits specific to OCD compared to other subtypes, whereas genes in spatial cluster 2 underlie traits shared by OCD with other subtypes like PD. For example, certain traits measured in the Padua inventory, such as impulsivity, cannot be used to discriminate OCD patients from PD and SAD patients.^22^ Therefore, spatial cluster 2 could have been influenced by GWAS genes linked to such traits that are shared between OCD and PD.

**Targets of anxiolytic drugs among AD-associated genes**

Although more than 900 drugs collectively targeted ~25% of the AD-associated genes, only 12 of these are currently targeted by anxiolytic drugs (**Supplementary Fig. 19a**). This indicated that the druggable AD gene space remains largely unexplored. Additionally, these 12 genes were not enriched for the regions to which AD-associated genes collectively converged (**Supplementary Fig. 19b**), suggesting that AD-associated regional specificities remain unaccounted for by the current standard treatment regimen. AD-associated genes that are currently drugged are more likely to be involved in monoamine (primarily 5-HT) transport (**Supplementary Fig. 19c**). However, these genes were collectively targeted by a fewer number of drugs than those that target a single GABA receptor (**Supplementary Fig. 19c**). This is likely due to the fewer instances of treatment withdrawals and adverse events, better symptom alleviation and tolerance reported for benzodiazepines (acting on GABA receptors) compared with tricyclic antidepressants (which are mostly selective 5-HT reuptake inhibitors) in ADs.^23^ Nevertheless, it is important to devise new strategies that are informed by disease mechanisms and tap synergistically into several ‘areas’ of the AD drug gene space, given that only 60-85% of AD patients experience at least 50% improvement in their symptoms with the current treatment strategies, and most of them suffer from recurrent or persistent anxiety symptoms.^24^

**Limitations of the study**

Our study has several limitations. Firstly, we assumed that the gene expression profiles of the brain structures from the left and right hemispheres would be symmetric and did not quantify the corresponding expression variations. Secondly, in **Fig. 3a-c**, we simplified the complex spatial expression patterns observed in **Fig. 2a** to a list of enriched regions identified from gene populations. Although our approach helped identify AD-associated structures, it did not allow us to elucidate the contribution of fine-grained expression variations to AD etiology. Nevertheless, the different methods used for examining the expression patterns in **Fig. 2a** (hierarchical clustering analysis) and **Fig. 3a-c** (enrichment analysis) can explain the presumed discrepancies between the heat map and the enrichment plot. For example, although a few spatial cluster 2 genes are highly expressed in the hippocampus (**Fig. 2a**), the region does not appear in the context of spatial cluster 2 in the enrichment plot (**Fig. 3a**). This is because a higher number of genes showing relatively higher expression in the hippocampus (compared to other regions) were present in spatial cluster 1 and not spatial cluster 2. Thirdly, despite their random distribution in the four temporal clusters (**Fig. 7a**), housekeeping genes could have influenced their temporal patterns (**Fig. 7b**). Lastly, the PyschENCODE single-cell transcriptome dataset (**Supplementary Fig. 20**) was extracted from the human dorsolateral prefrontal cortex, limiting the relevance of the result.

**
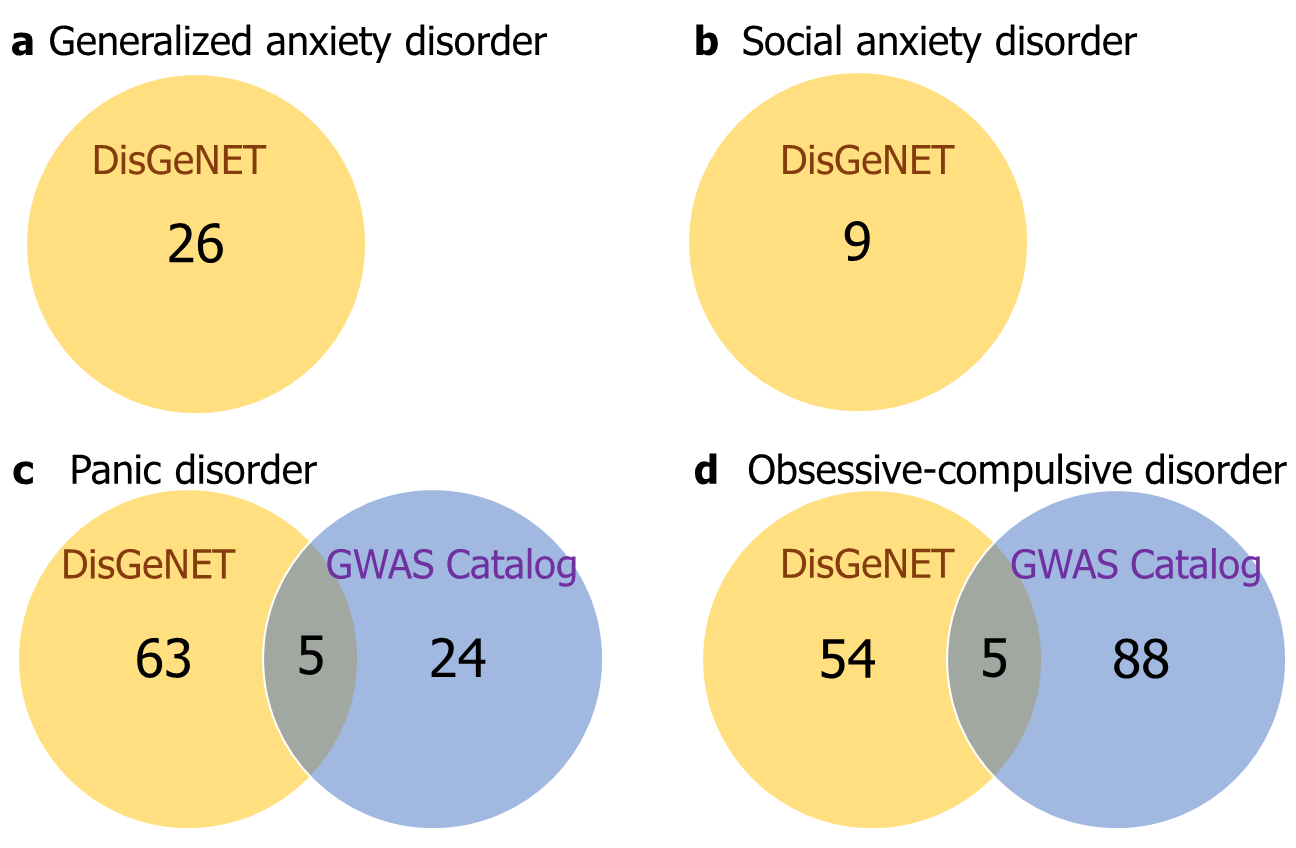
Supplementary Fig. 1: AD-associated genes compiled from the GWAS catalog and the DisGeNET database.** The number of genes associated with the four anxiety disorder subtypes – **a** generalized anxiety disorder (GAD), **b** social anxiety disorder (SAD), **c** panic disorder and **d** obsessive-compulsive disorder (OCD) – compiled from the DisGeNET database and the GWAS catalog. These genes were found within 50 kb upstream or downstream of the SNPs associated with the AD subtypes.

**
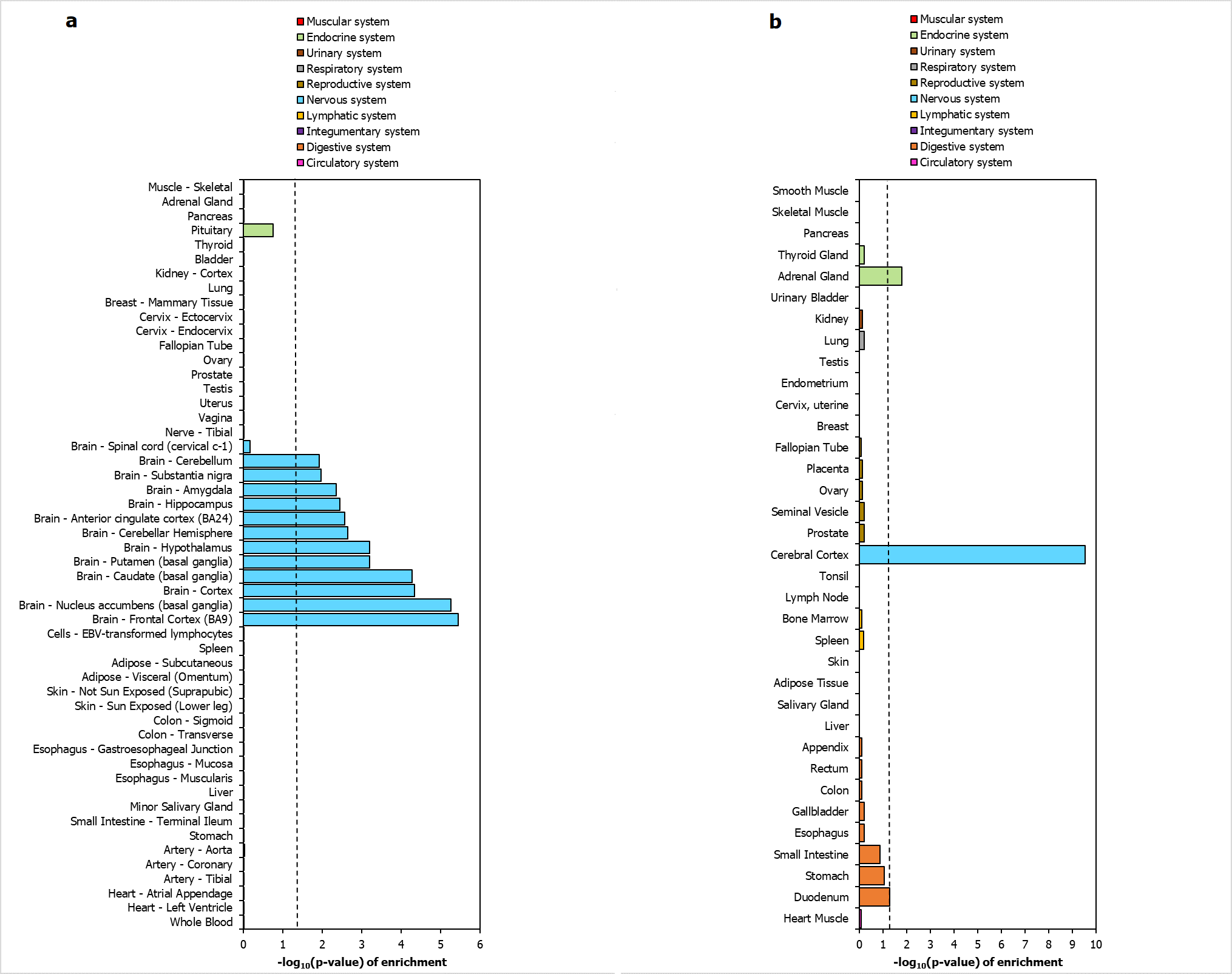
Supplementary Fig. 2: AD-associated genes were selectively expressed in the nervous system.** The figure shows tissue-specific enrichment results for 240 AD-associated genes according to data from **a** GTEx and **b** Human Protein Atlas. Genes (excluding housekeeping genes) that showed high or moderate expression (transcripts per million (TPM) ≥ 9) in 52 GTEx tissues and elevated expression level (TPM > 1 and 4-folds higher in the brain compared to any other tissue/all the other tissues or in a group of tissues including the brain compared to any other tissue) in 35 tissues from the Human Protein Atlas were considered. The dotted black line indicates the cut-off value for statistical significance, after correction for multiple hypotheses using the Benjamini-Hochberg method (p-value < 0.05, log_10_(p-value) > 1.30103).

**
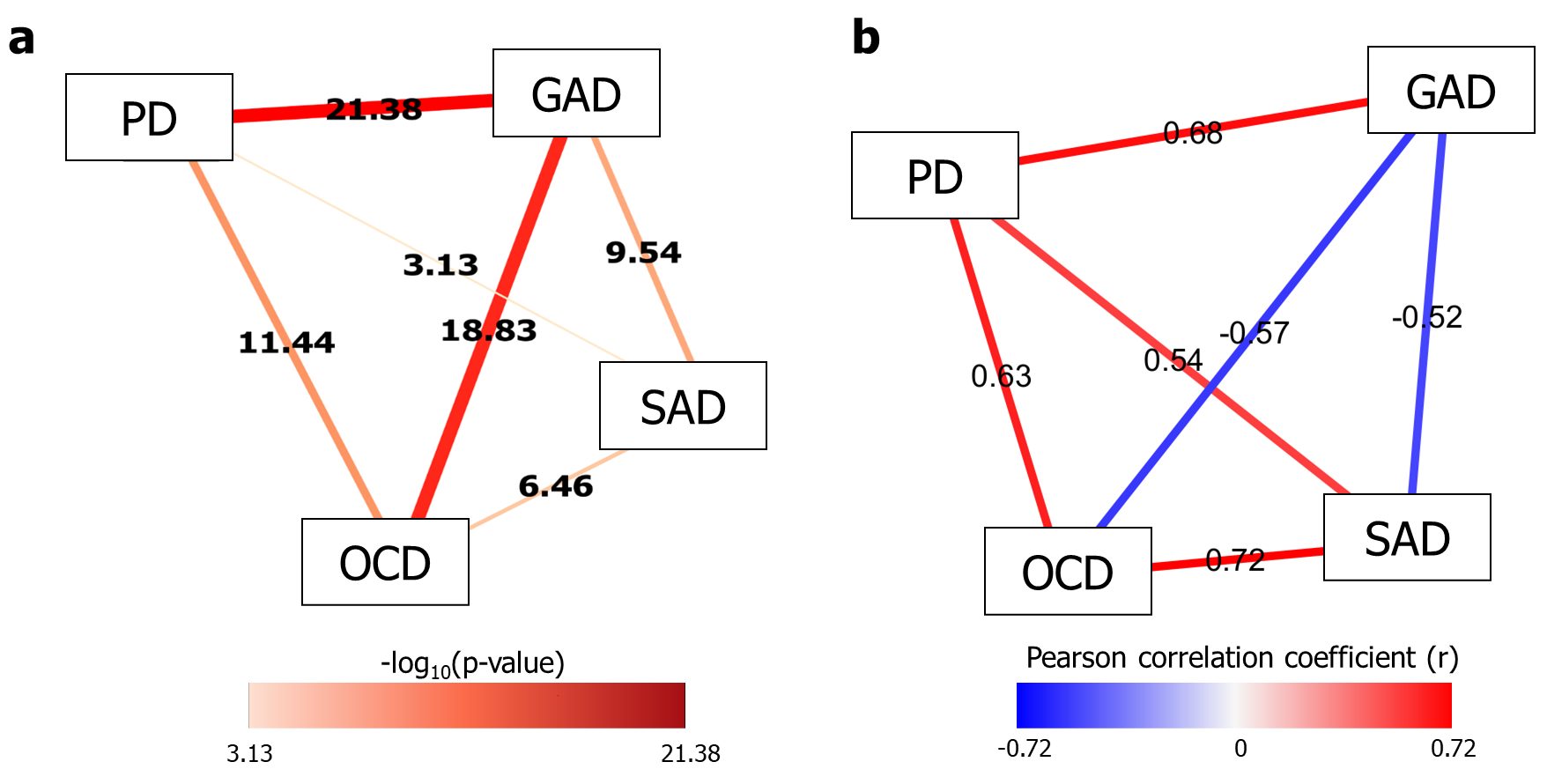
Supplementary Fig. 3: Relationships between AD subtypes. a** –log_10_(p-values) of overlap, which indicate the statistical significance of the overlaps between pairs of AD subtypes, have been depicted as a network. Each node represents an AD subtype, and each edge connecting two AD subtypes represents the pairwise relationship exhibited by these subtypes, in terms of shared genes. The width of the edge increases with increasing statistical significance of the overlap and vice versa. The edges have also been colored to indicate higher (darker red) and lower (lighter red) levels of statistical significance respectively. **b** Pearson correlation coefficients (PCCs), which indicate the strength of correlation between the expression profiles of AD subtype-specific genes in cerebral nuclei, limbic system and midbrain regions, have been depicted as a network. Each node represents an AD subtype, and each edge connecting two AD subtypes represents the PCC between the subtypes.

**
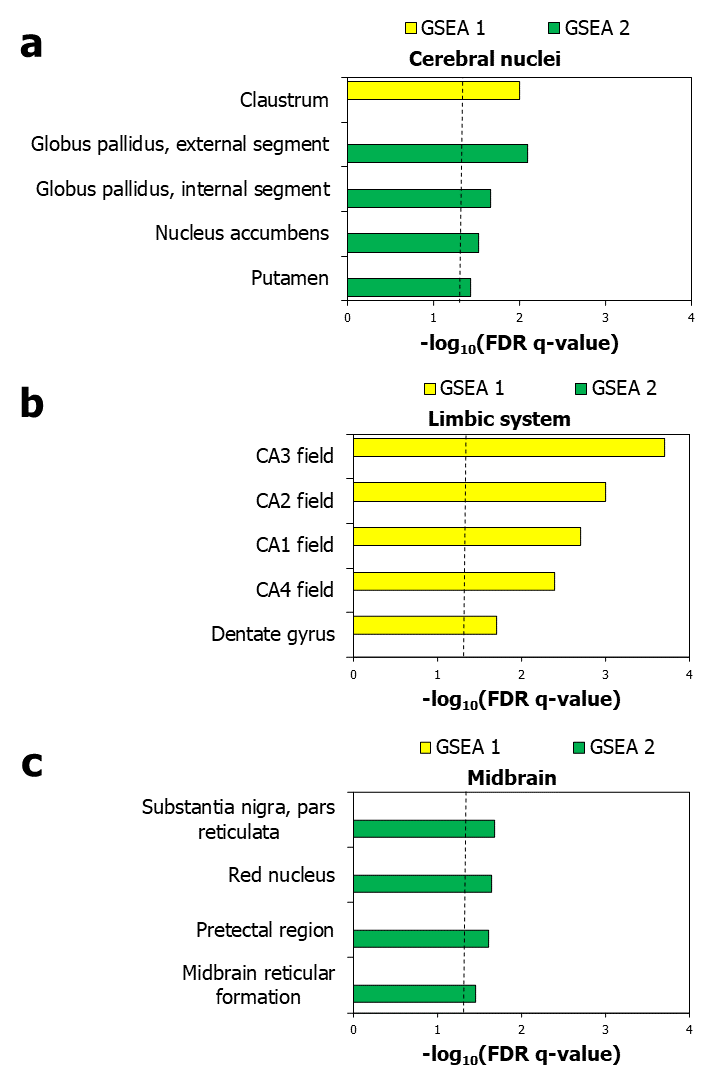
Supplementary** **Fig. 4: Differential regional enrichment patterns of the spatial clusters obtained from gene set enrichment analysis (GSEA).** The figure shows the enrichment of spatial cluster 1 and spatial cluster 2 for genes showing relatively higher expression in **a** cerebral nuclei samples, **b** limbic systems samples and **c** midbrain samples, compared to other regions. The dotted black line in **a** – **c** indicates the cut-off value for –log_10_(FDR-corrected q-value) (q-value < 0.05, log_10_(q-value) > 1.30103).

**
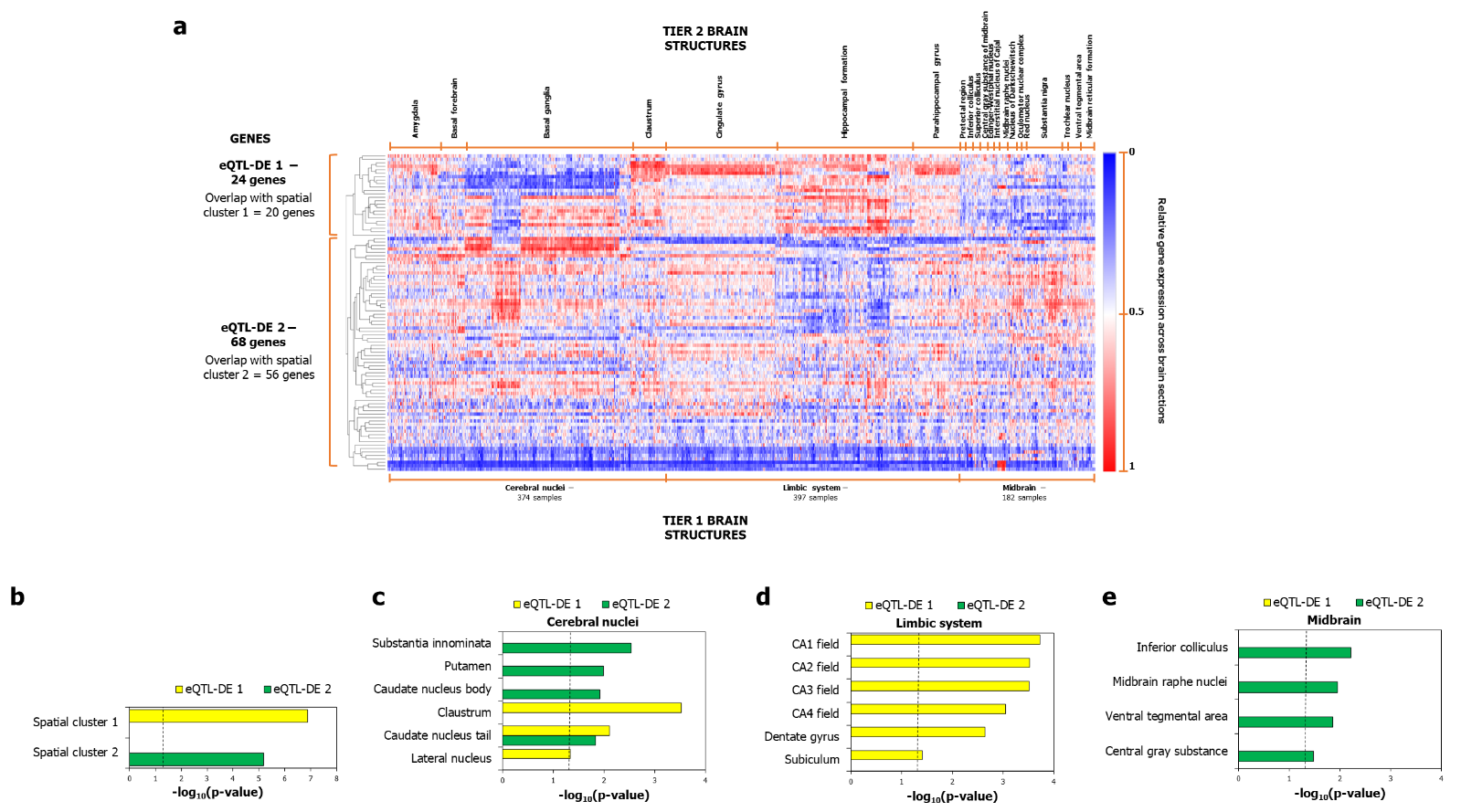
Supplementary Fig. 5: Recapitulation of the spatial clusters using AD genes with transcriptomic evidence. a** The figure shows the dichotomized expression of 92 AD-associated genes supported by eQTL or differential gene expression evidence or both, across 374 cerebral nuclei, 397 limbic system and 182 midbrain samples in the Human Adult Microarray Data (Allen Brain Atlas). It also shows the p-values of overlap of the detected clusters 1 and 2 with spatial clusters 1 and 2. Relative gene expression of each of the genes across the 953 brain sections was hierarchically clustered by computing pairwise distances between the data points (log_2_-transformed probe intensities) using Pearson correlation and identifying closely linked clusters using the average linkage method. The clustered heat map was generated using the Morpheus software. **b** shows the enrichment of the clusters derived in **a** for the two spatial clusters derived from the original AD set (which contained genes with and without transcriptomic evidence. **c**-**e** show the enrichment of cluster 1 and cluster 2 for genes showing relatively higher expression in **c** cerebral nuclei samples, **d** limbic systems samples and **e** midbrain samples, compared to other regions. The dotted black line in **b** – **e** indicates the cut-off value for –log_10_(p-value) after correction for multiple hypotheses using the Benjamini-Hochberg method (p-value < 0.05, log10(p-value) > 1.30103).

**
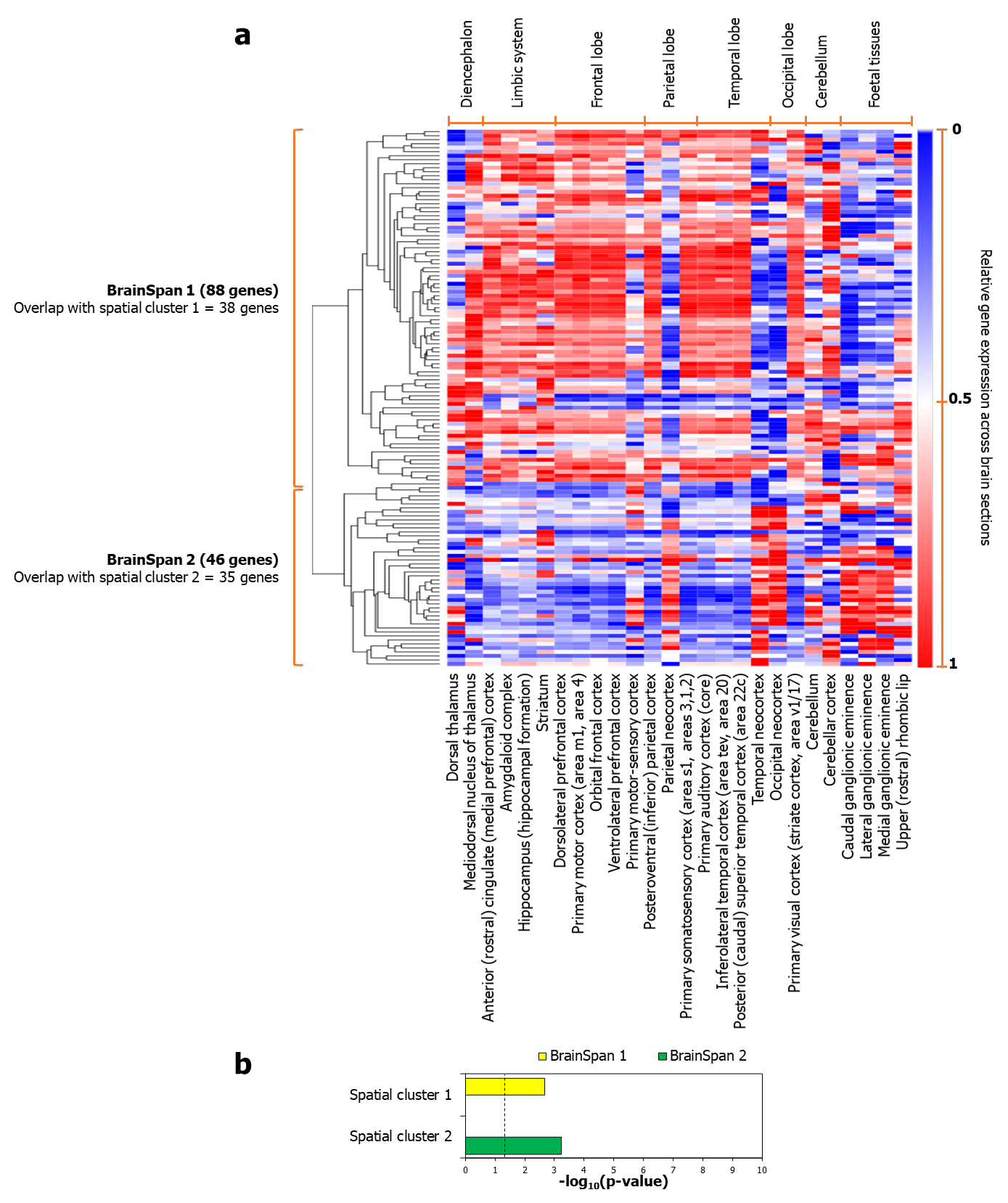
Supplementary Fig. 6: Recapitulation of the spatial clusters using RNA-sequencing data in BrainSpan Atlas.** The figure shows the dichotomized expression of 134 AD-associated genes across 524 samples in the BrainSpan Atlas, with the RPKM values averaged across 26 brain structures (bottom horizontal axis). It also shows the p-values of overlap of the detected clusters 1 and 2 with spatial clusters 1 and 2. Relative (average) gene expression of each of the genes across the 26 brain structures was hierarchically clustered by computing pairwise distances between the data points (log_2_-transformed RPKM values) using Pearson correlation and identifying closely linked clusters using the average linkage method. The clustered heat map was generated using the Morpheus software. **b** shows the enrichment of the clusters derived in **a** for the two spatial clusters derived from the original AD set (using the microarray dataset).

**
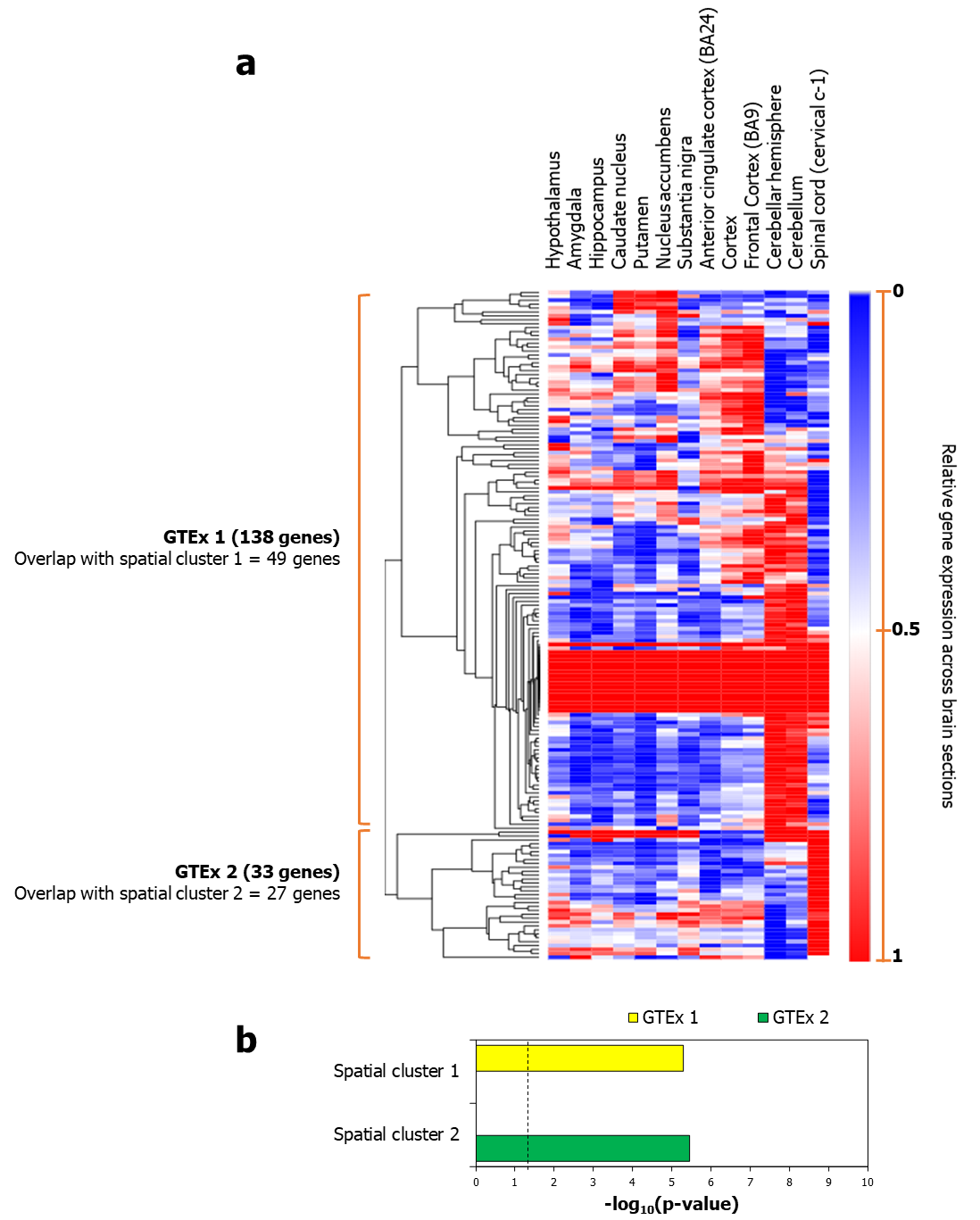
Supplementary Fig. 7: Recapitulation of the spatial clusters using RNA-sequencing data in GTEx.** The figure shows the dichotomized expression (available as median TPM values) of 171 AD-associated genes across 13 brain structures in GTEx. It also shows the p-values of overlap of the detected clusters 1 and 2 with spatial clusters 1 and 2. Relative gene expression of each of the genes across the 13 brain structures was hierarchically clustered by computing pairwise distances between the data points (log_2_-transformed TPM values) using Pearson correlation and identifying closely linked clusters using the average linkage method. The clustered heat map was generated using the Morpheus software. **b** shows the enrichment of the clusters derived in **a** for the two spatial clusters derived from the original AD set (using the microarray dataset).

**Supplementary Fig. 8: Recapitulation of the influence of specific brain regions on the spatial clusters by principal component analysis (PCA).** Component loadings of **a** 374 cerebral nuclei samples, **b** 397 limbic system samples and **c** 182 midbrain samples contributing to PC1 and PC2 shown in Fig. 4a were plotted along X and Y axes respectively. Component loadings are values depicting the correlation of the original variables in our data matrix — log_2_-transformed probe intensities — with each of the extracted principal components.


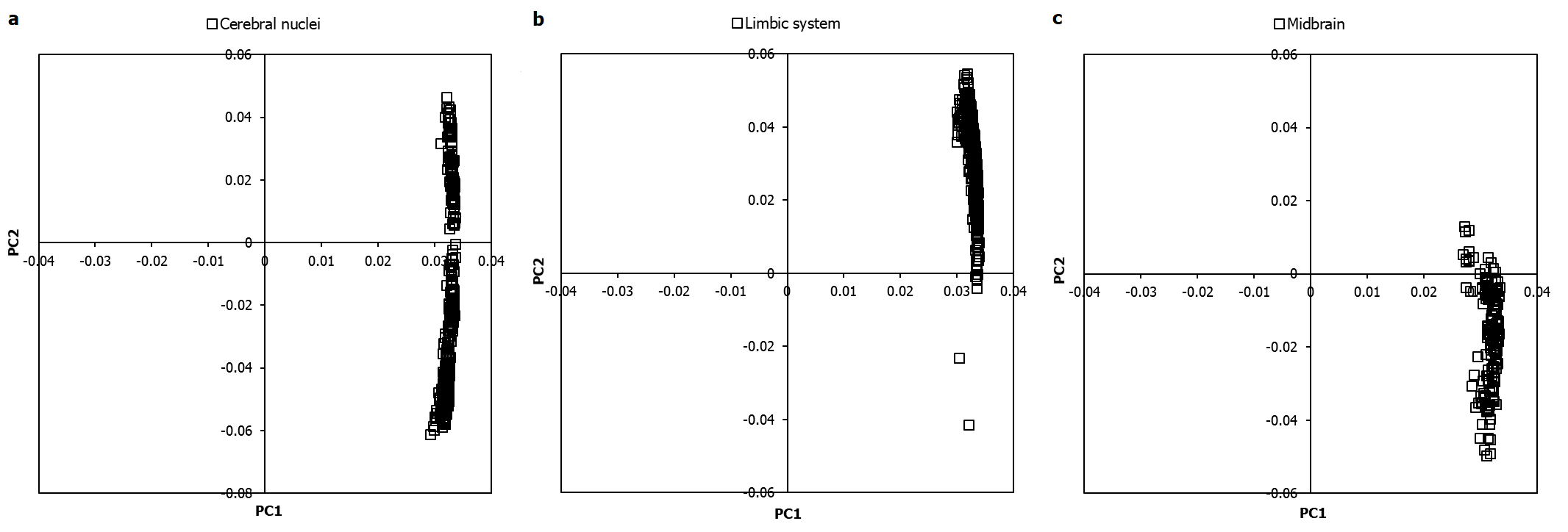


**Supplementary Fig. 9: Recapitulation of the regional specificities shown by spatial clusters using Principal Component Analysis (PCA).** The figure shows the enrichment of the genes in quadrants I-IV (as seen in Fig. 6a) for genes showing relatively higher expression in **a** cerebral nuclei samples, **b** limbic system samples and **c** midbrain samples, compared to other regions. The dotted black line in **a** – **c** indicates the cut-off value for –log_10_(p-value), after correction for multiple hypotheses using the Benjamini-Hochberg method (p-value < 0.05, log_10_(p-value) > 1.30103).


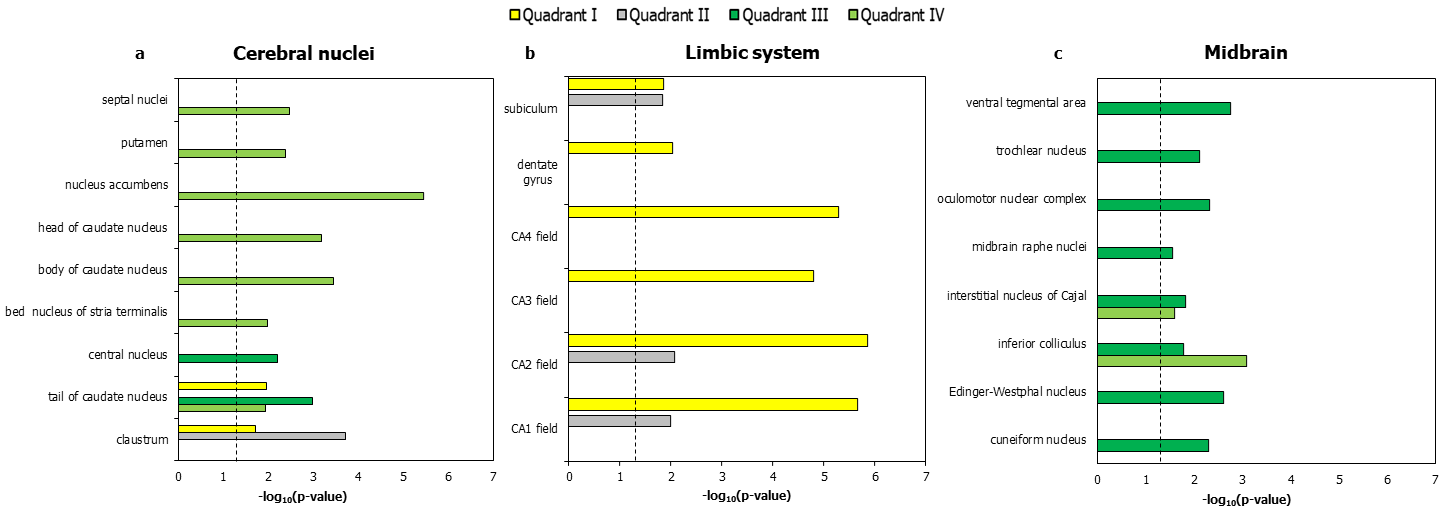


**
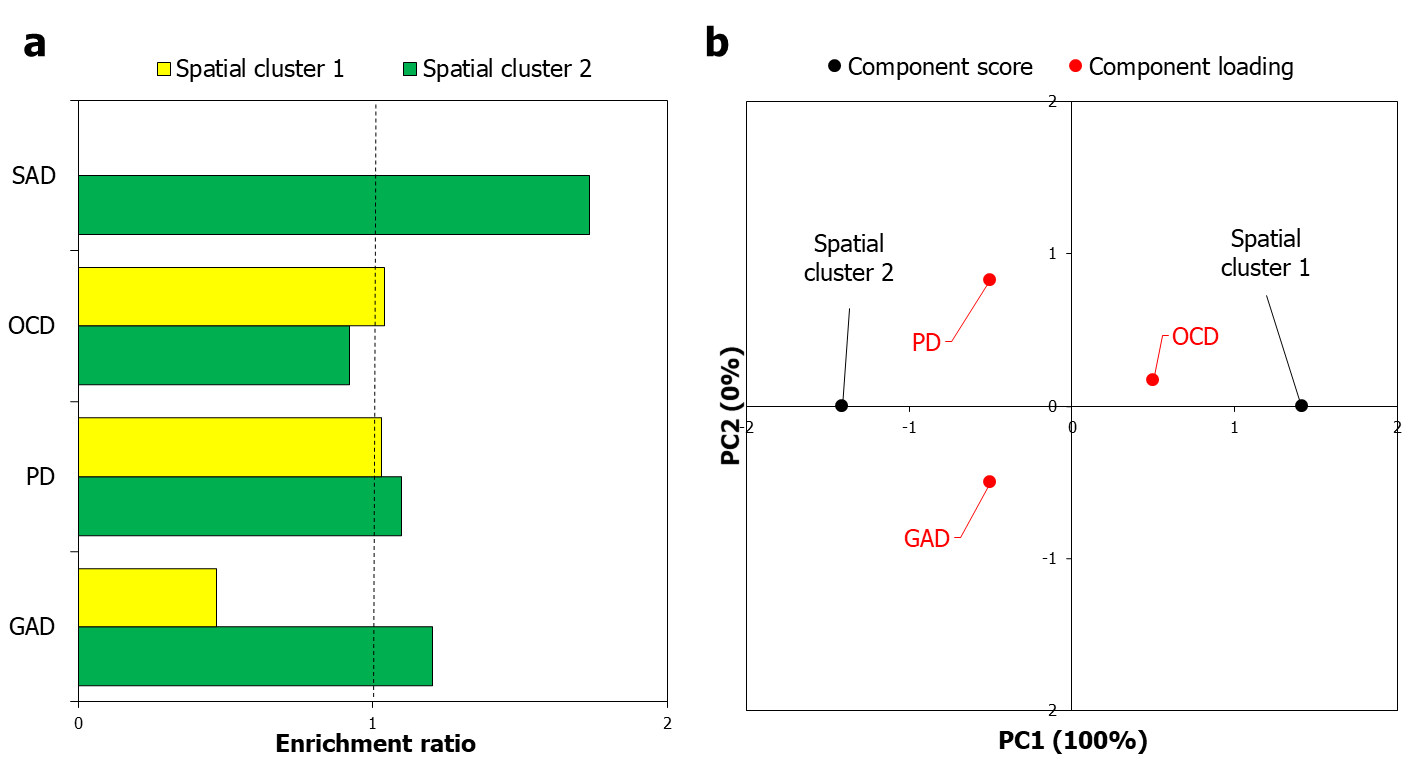
Supplementary Fig. 10: AD subtypes enriched in the spatial clusters.** **a** The enrichment of spatial cluster 1 and spatial cluster 2 for genes associated with four AD subtypes (in terms of enrichment ratio) has been shown. The dotted black line indicates the cut-off value for enrichment ratio, i.e. 1. The enrichment ratios were transformed to log_2_(enrichment ratios) values, which were then assembled into a data matrix containing the AD subtypes as rows and the spatial clusters as columns. **b** Component scores (black dots) of spatial cluster 1 and spatial cluster 2 – derived based on the enrichment ratios for AD subtypes – corresponding to PC1 and PC2 explaining 100% and 0% of the total variance were plotted along X and Y axes respectively. Component loadings (red dots) of 3 dimensions, i.e. AD subtypes, contributing to PC1 and PC2 were plotted along X and Y axes respectively.


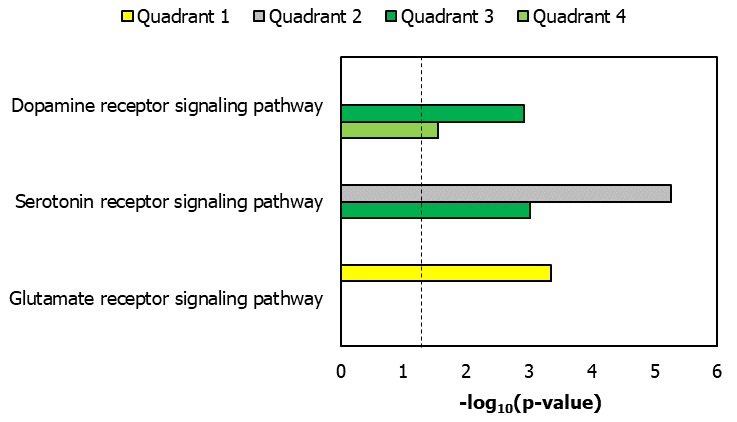
**Supplementary Fig. 11: Recapitulation of the signaling pathway enrichments shown by spatial clusters using Principal Component Analysis (PCA).** The figure shows the enrichment of spatial cluster 1 and spatial cluster 2 for genes involved in synaptic signalling pathways (in terms of –log_10_(p-values). The dotted black line indicates the cut-off value for –log_10_(p-value) after correction for multiple hypotheses using the Benjamini-Hochberg method, i.e. 1.30103.

**
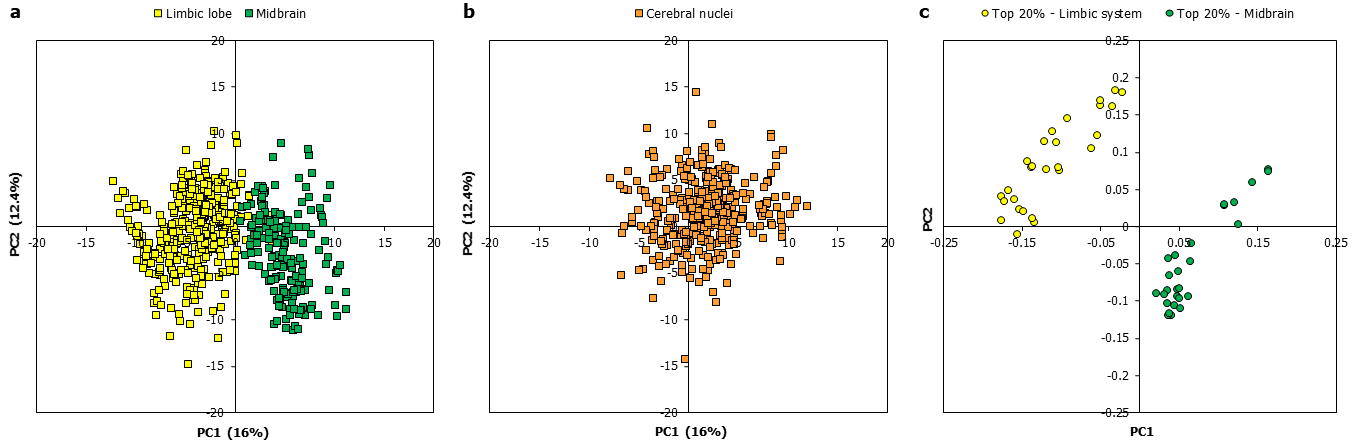
Supplementary Fig. 12: Top 20% genes that influenced the separation of the limbic-associated/midbrain-biased spatial clusters 1 and 2.** Principal component analysis (PCA) was performed with a matrix containing genes (rows) × brain samples (columns); the cells contained gene probe intensities in specific samples. Unit variance scaling was applied across this matrix. Single value decomposition with imputation was used to extract the principal components (PCs). Component scores corresponding to PC1 and PC2 explaining 16% and 12.4% of the total variance were plotted along X and Y axes respectively for **a** limbic and midbrain samples and **b** cerebral nuclei samples. Component loadings of the top 20% genes contributing to limbic and midbrain samples shown in **a** were plotted in **c**.

**
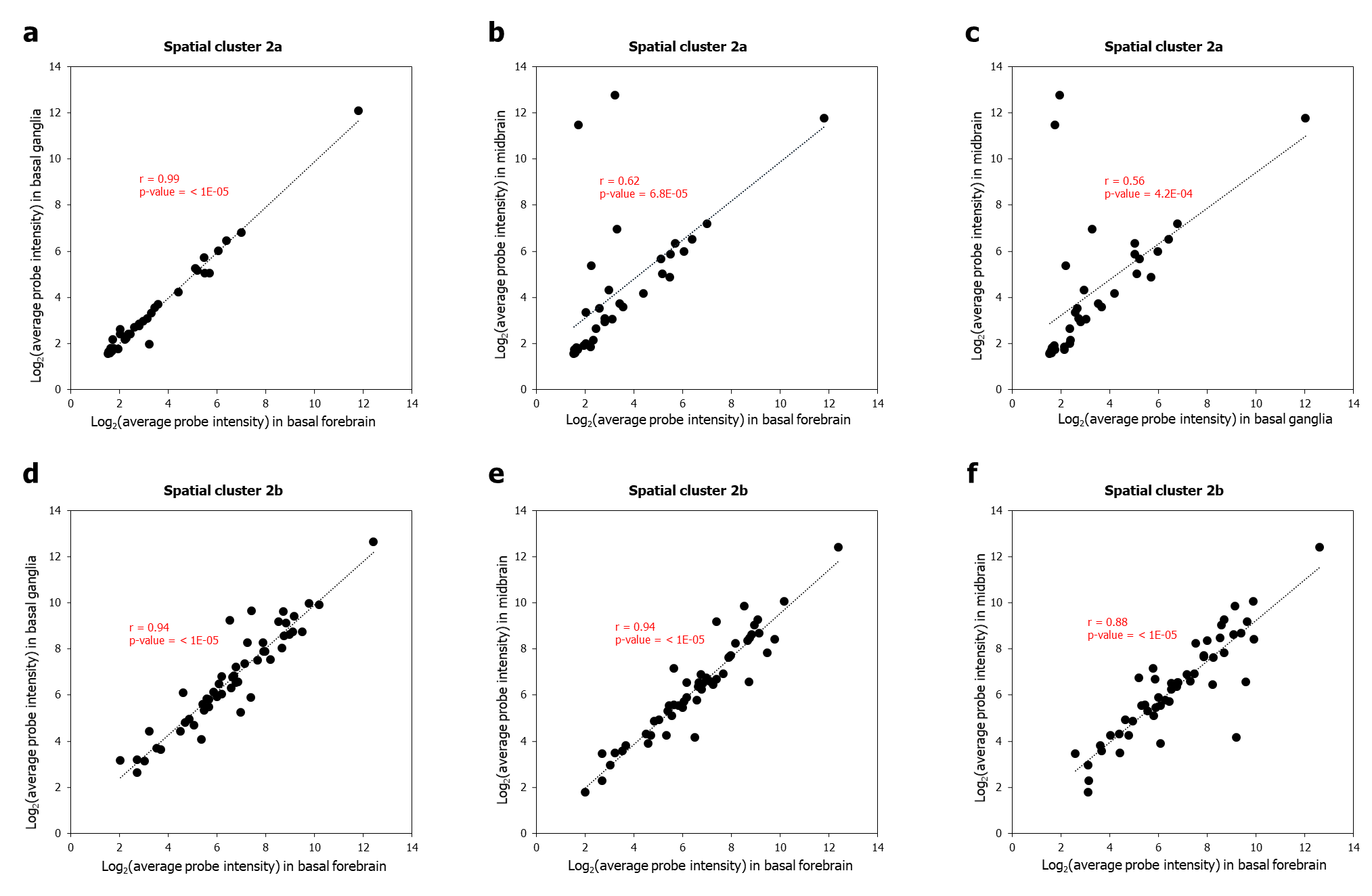
Supplementary Fig. 13: Correlated expression patterns of the sub-clusters 2a and 2b in midbrain, basal forebrain and basal ganglia.** The figures show the correlation of log_2_(average probe intensity) values of spatial cluster 2a genes in **a** basal forebrain (X-axis) versus basal ganglia (Y-axis), **b** basal forebrain versus midbrain and **c** basal ganglia versus midbrain, and spatial cluster 2b genes in **d** basal forebrain versus basal ganglia, **e** basal forebrain versus midbrain and **f** basal forebrain versus midbrain. Pearson correlation coefficient (r) was used to examine the relationship between the expression values.

**Supplementary Fig. 14: The co-expression network containing 92 genes that overlaps with spatial cluster 1. a** The figure shows 92 AD genes that form a co-expression network. Each node represents an AD gene, and each edge connecting two AD genes represents the Pearson correlation coefficient (PCC) between the genes. Edges have been colored from lighter to darker shades of red to indicate lower to higher PCC. **b** shows the enrichment of this 92-genes co-expression network in specific brain structures (in terms of –log_10_(p-values). **c** shows the enrichment of the network for genes involved in various Gene Ontology biological processes. In **b** and **c**, the brain structures and pathways enriched in spatial cluster 1 have been marked with asterisks. The dotted black lines in **b** and **c** indicate the cut-off value for –log_10_(p-value) after correction for multiple hypotheses using the Benjamini-Hochberg method (p-value < 0.05, log_10_(p-value) > 1.30103).


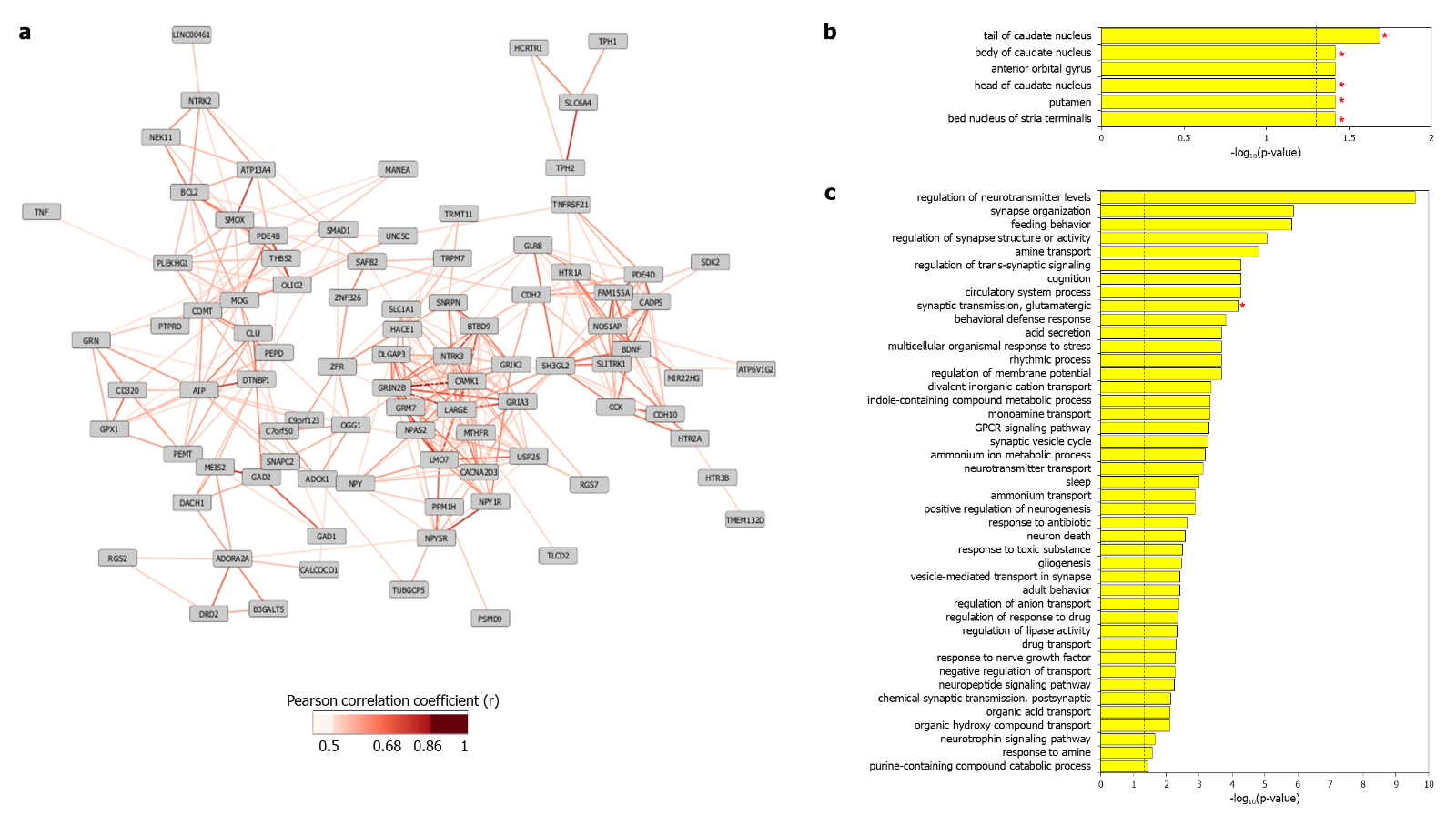


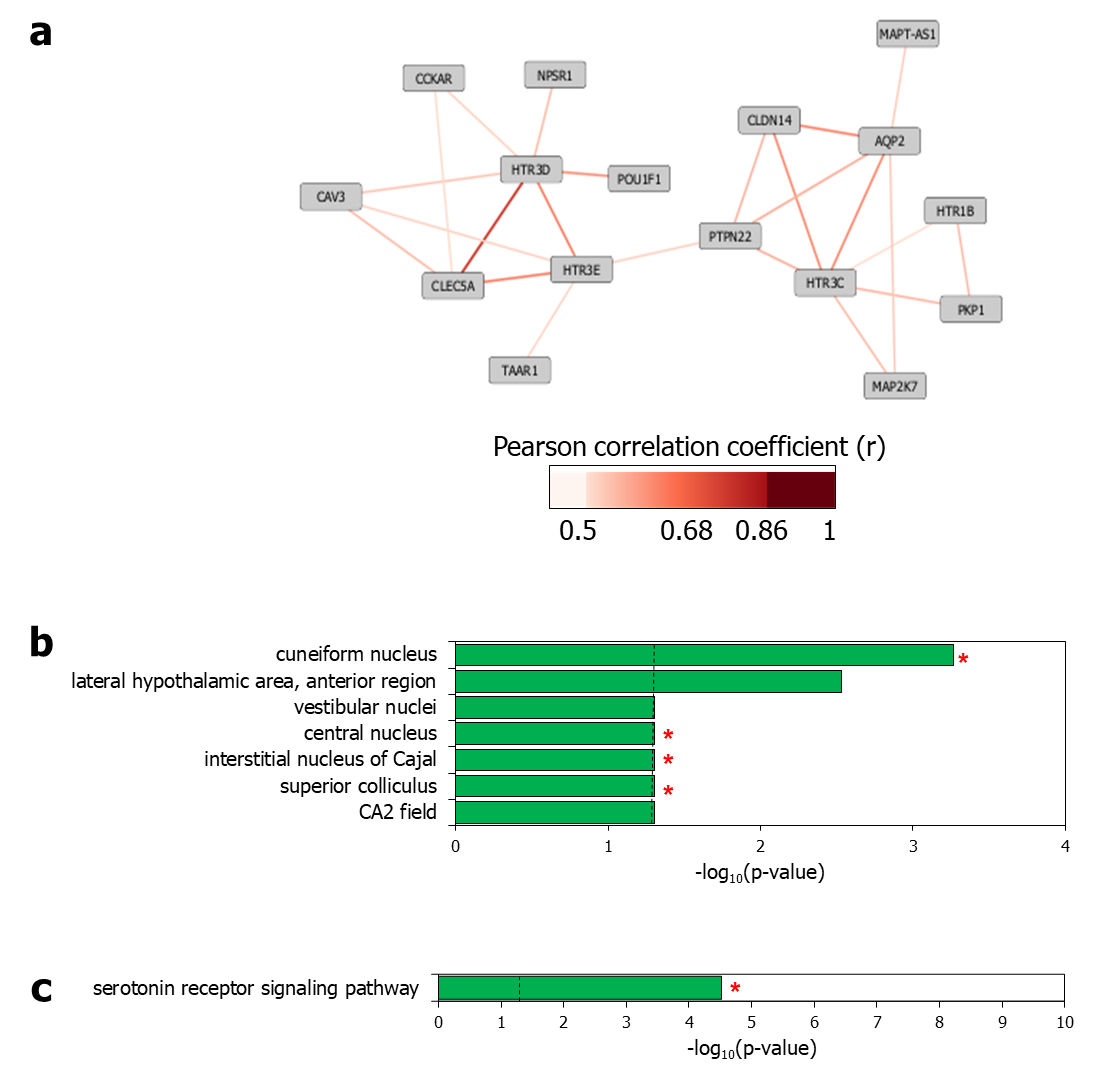


**Supplementary Fig. 15: The co-expression network containing 16 genes that overlaps with spatial cluster 2. a** The figure shows 16 AD genes that form a co-expression network. Each node represents an AD gene, and each edge connecting two AD genes represents the Pearson correlation coefficient (PCC) between the genes. Edges have been colored from lighter to darker shades of red to indicate lower to higher PCC. **b** shows the enrichment of this 16-genes co-expression network in specific brain structures (in terms of –log_10_(p-values). **c** shows the enrichment of the network for genes involved in various Gene Ontology biological processes. In **b** and **c**, the brain structures and pathways enriched in spatial cluster 2 have been marked with asterisks. The dotted black lines in **b** and **c** indicate the cut-off value for –log_10_(p-value) after correction for multiple hypotheses using the Benjamini-Hochberg method (p-value < 0.05, log_10_(p-value) > 1.30103).


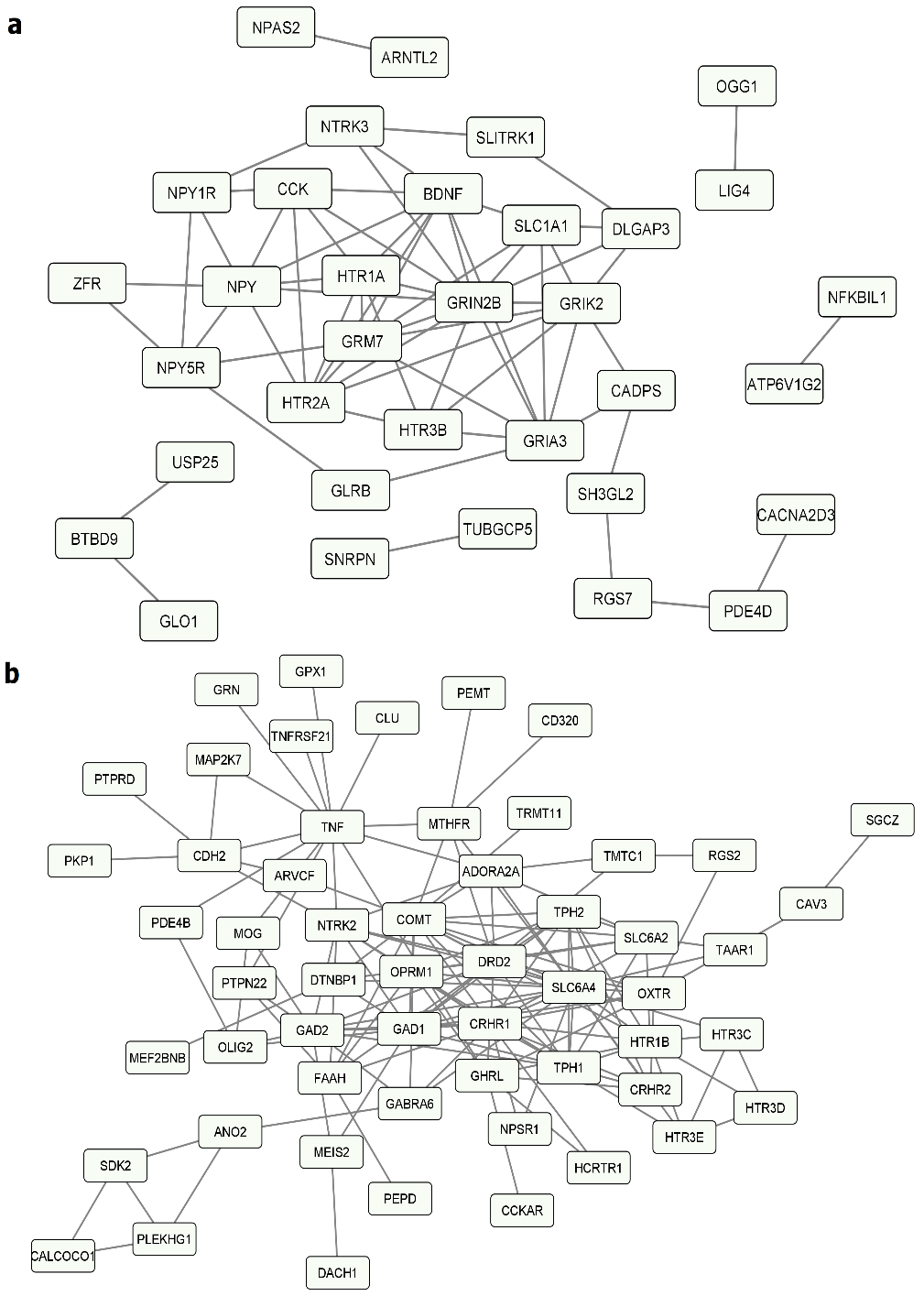
**Supplementary Fig. 16: Interconnectivity of the spatial cluster genes.** The network diagrams show the associations between the proteins encoded by the genes belonging to **a** spatial cluster 1 (number of edges = 65, odds ratio = 6.5, p-value < 1E-16) and **b** spatial cluster 2 (number of edges = 149, odds ratio = 4.8, p-value < 1E-16). Network nodes represent proteins and edges represent protein-protein associations ranging from joint contribution to shared functions to direct physical interactions retrieved from the STRING database.

**
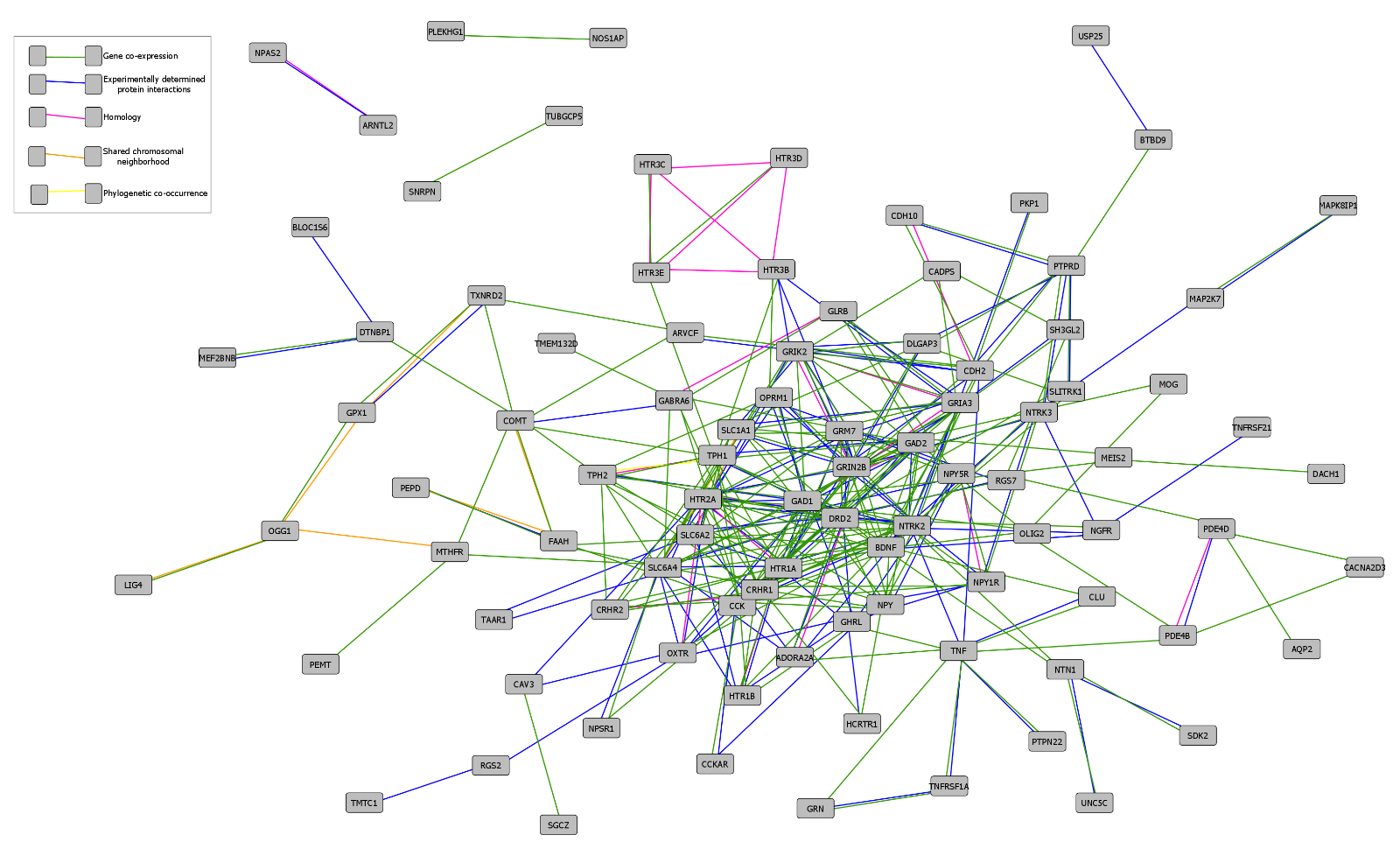
**

**Supplementary Fig. 17: Network of AD-associated genes.** The figure shows the gene network interconnecting 93 AD-associated genes through 380 functional associations. Network nodes represent proteins and edges represent protein-protein associations ranging from joint contribution to shared functions to direct physical interactions retrieved from the STRING database. As shown in the legend, the colors of the edges denote the various types of associations.

**
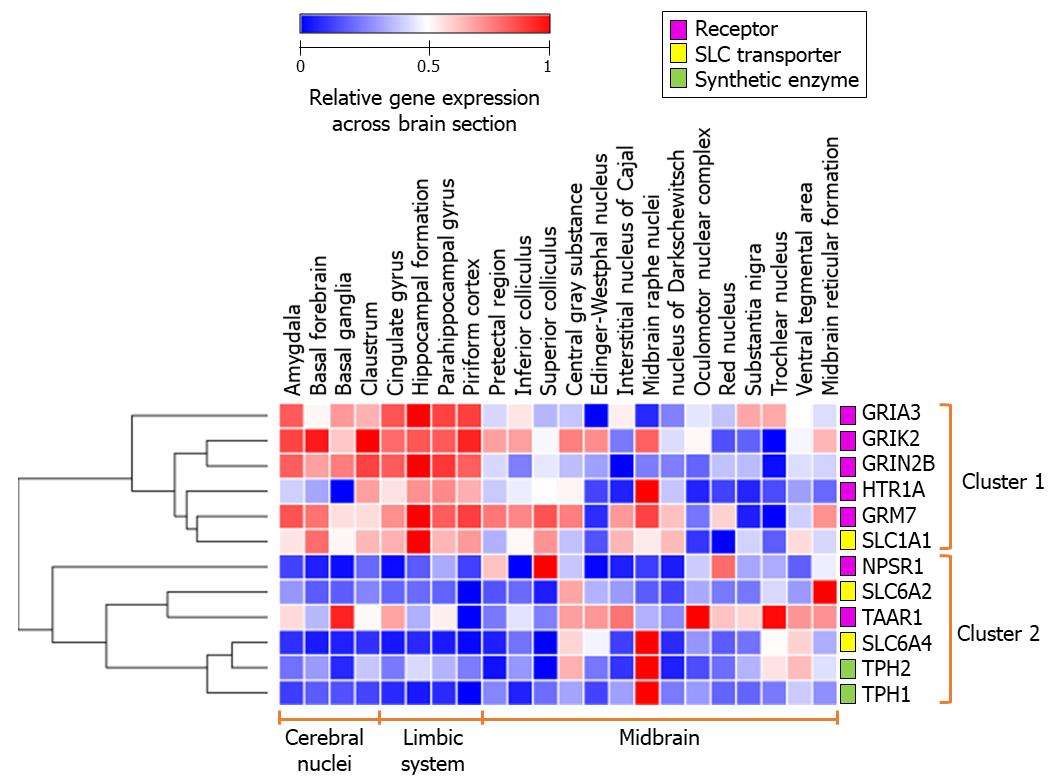
Supplementary Fig. 18: Spatial expression patterns of the functional network genes.** The heat map captures the spatial expression patterns of the genes in the functional networks shown in Fig. 5e and Fig. 5i across cerebral nuclei, limbic system and midbrain samples and their membership in the two spatial clusters.

**
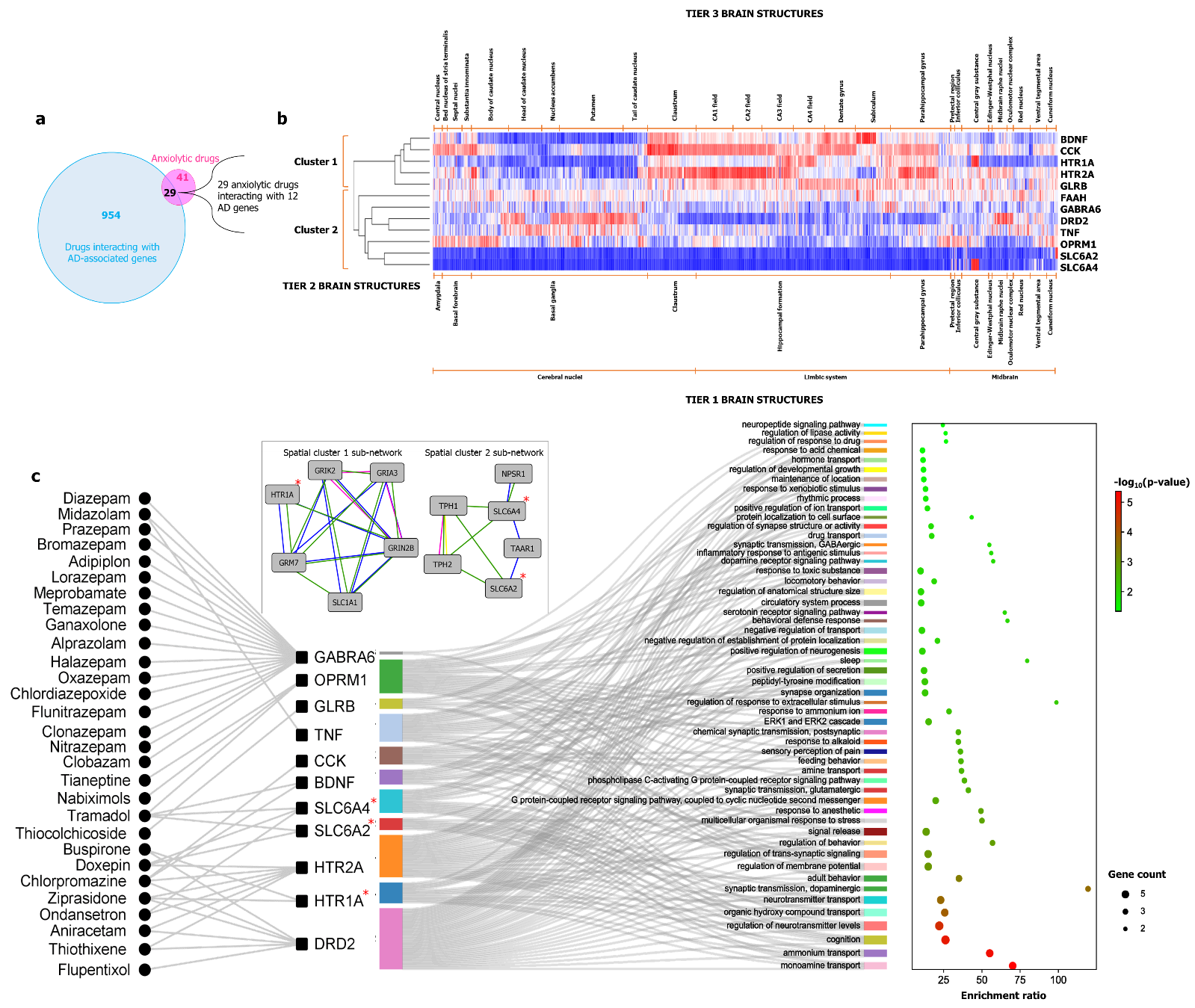
Supplementary Fig. 19: Anxiolytic drugs interacting with AD-associated genes.** **a** The identification of 29 anxiolytic drugs that interact with 12 AD-associated genes by intersecting the list of 70 anxiolytic drugs compiled from the DrugBank database with the list of 983 drugs interacting with 63 AD-associated genes. **b** Region-specific expression patterns of the 12 AD-associated genes – split into cluster 1 and cluster 2 – have been presented in the form of a heatmap. **c** The 29 anxiolytic drugs, the 12 AD-associated genes that they interact with and the GO Biological Processes that these genes are enriched in have been shown in the form of a Sankey plot. The square-shaped nodes depict the AD-associated genes and the circular nodes depict the anxiolytic drugs. These genes are further connected to the biological processes, and the width of the colored boxes next to each of the genes varies according to the number of processes in which they are involved. The X-axis of the dot plot shows the enrichment ratio of the GO biological processes. The colors of the dots represent their FDR-corrected –log_10_(p-values) of enrichment and the size of the dots represents the gene count.

**Supplementary Fig. 20: Assessment of the cell-type specificity of the spatial clusters using PsychENCODE data.** The heat map was derived from the hierarchical clustering of AD-associated genes based on the Pearson correlation between their cell-specificity scores in 29 transcriptomically distinct cell types of the human dorsolateral prefrontal cortex.


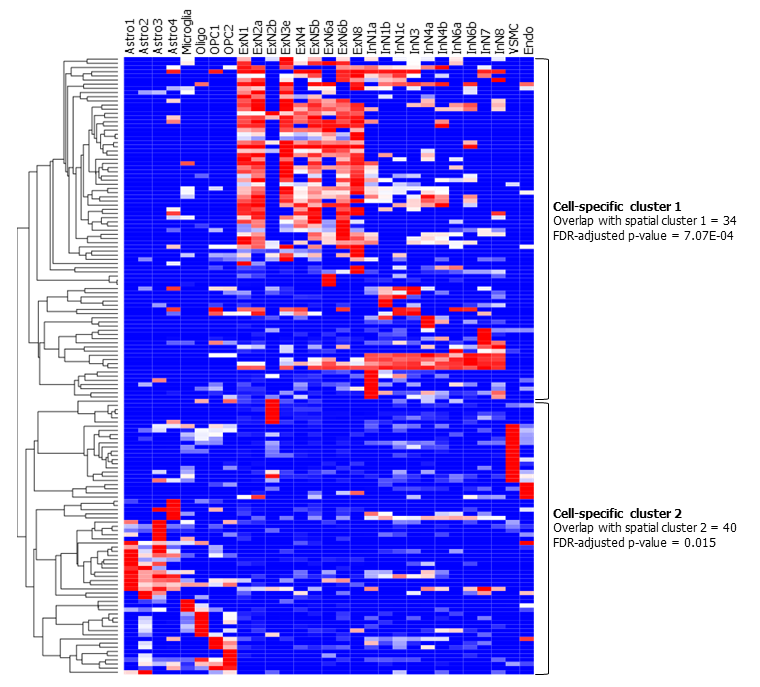


**
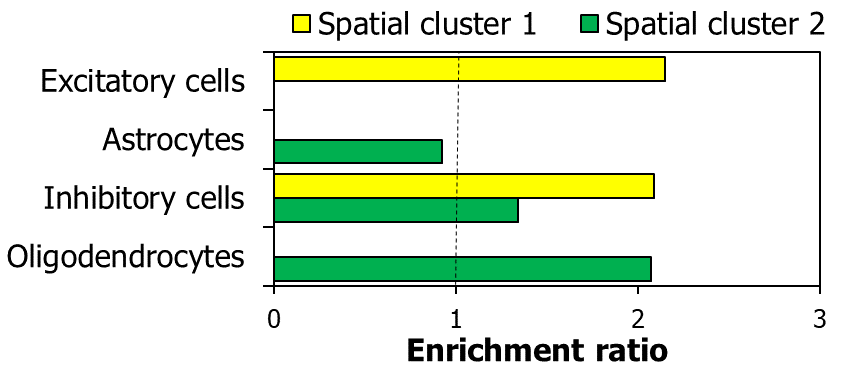
Supplementary Fig. 21: Assessment of the cell-type specificity of the spatial clusters using data from Lake et al.:** The enrichment of spatial cluster 1 and spatial cluster 2 for genes expressed in specific brain cell types (in terms of enrichment ratio) has been shown. The dotted black line indicates the cut-off value for enrichment ratio, i.e. 1.

**
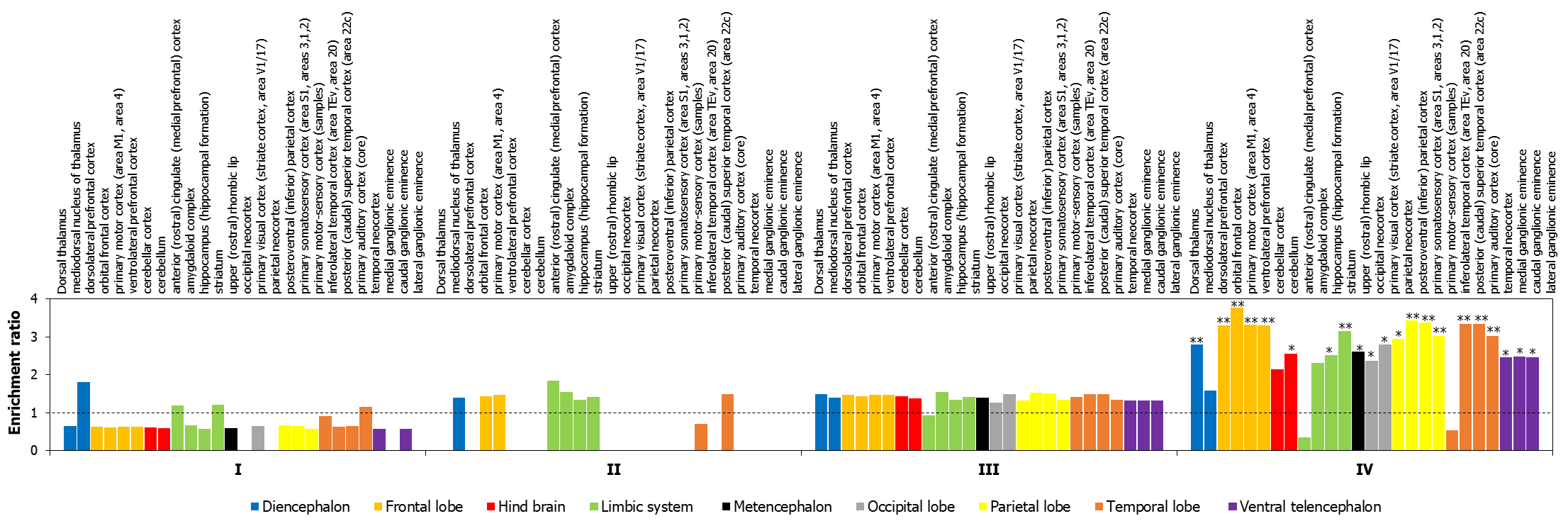
Supplementary Fig. 22: Enrichment of the temporal clusters for genes expressed in specific brain regions in the developmental transcriptome (BrainSpan).** The figure shows the enrichment ratios of the temporal clusters shown in Fig. 7a across 26 brain regions represented in the BrainSpan developmental transcriptome data available in Allen Brain Atlas. Genes expressed at logRPKM > 2 in a specific region, where RPKM is Reads Per Kilobase per Million mapped reads, excluding housekeeping genes (detected in all the tissues with TPM ≥ 1) were included in the analysis. The 26 brain regions (shown on the horizontal axis at the top) were segmented into 9 areas (shown in the legend). The bars – depicting the enrichment ratio of each of the 26 brain regions in the temporal clusters I-IV – have been colored according to their membership in each of the 9 areas. *, **, and *** indicate p-value < 0.5, < 0.01 and < 0.001 after multiple test adjustment using the Benjamini-Hochberg method. The dotted black line indicates the cut-off value for enrichment ratio, i.e. 1.

**
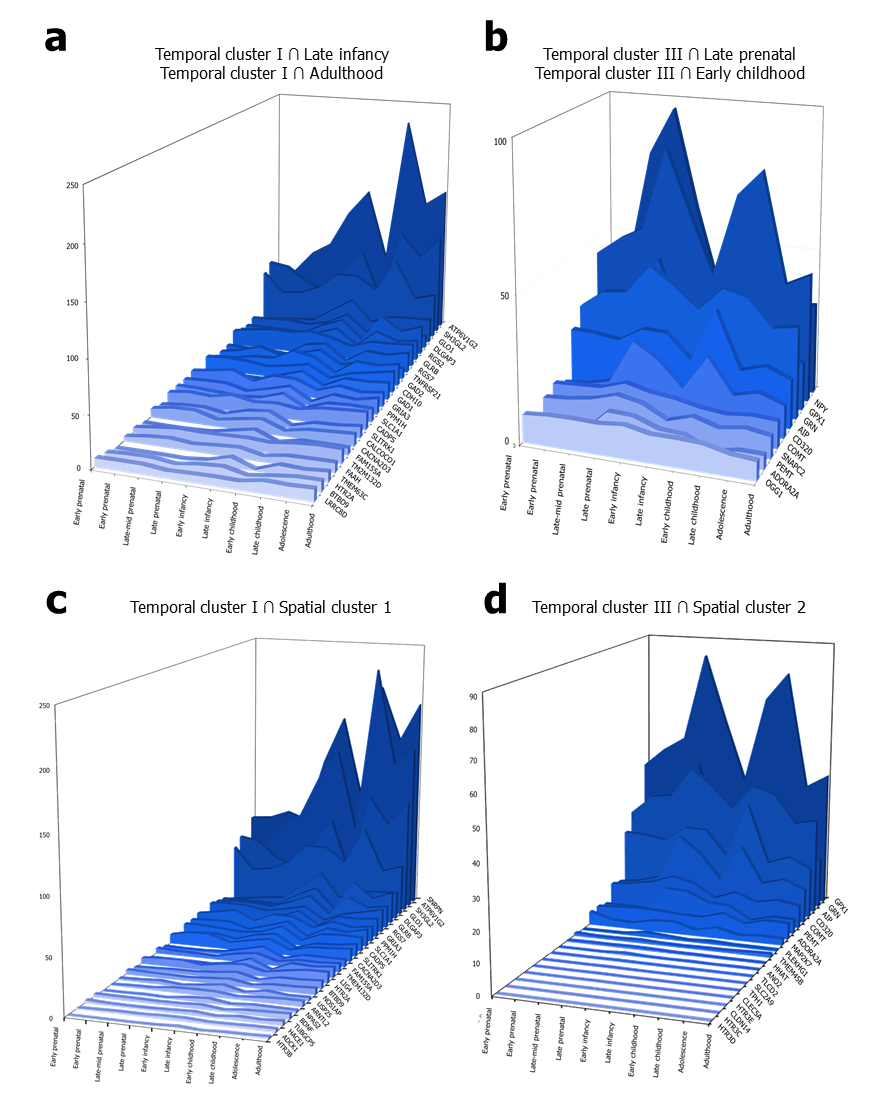
Supplementary Fig. 23: Genes in temporal clusters belonging to specific developmental stages and spatial clusters.** The average RPKM of the genes in temporal cluster I that show high expression in late infancy and adulthood stages has been shown in **a**. The same for temporal cluster III genes highly expressed during late prenatal and early childhood stages has been shown in **b**. In **c** and **d**, the average RPKM of the genes in temporal clusters I and III that are also found in spatial cluster 1 and spatial cluster 2 respectively has been shown.

**
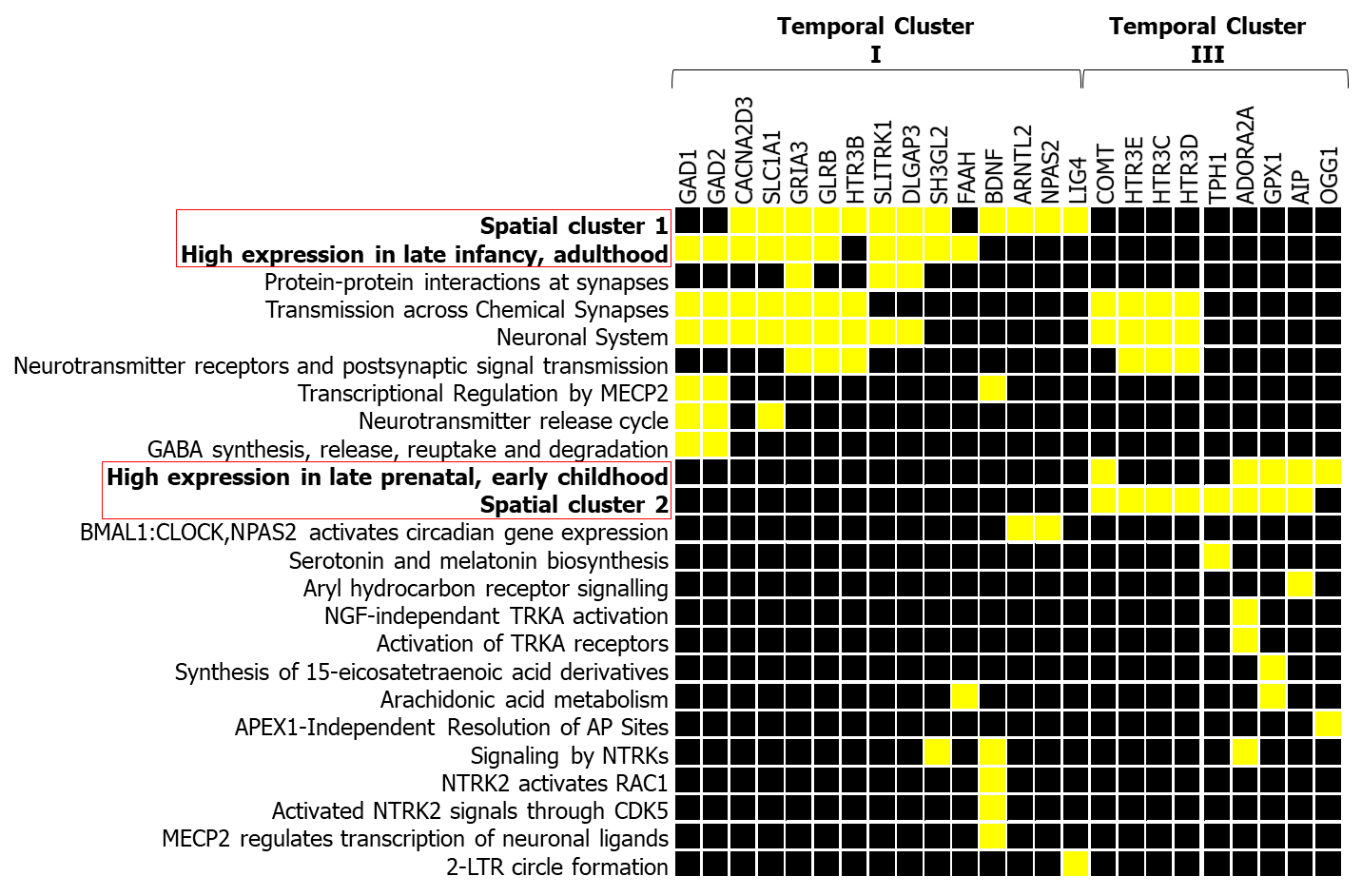
Supplementary Fig. 24: Pathways in which temporal clusters I and III are involved.** The figure shows a few genes from temporal clusters I and III, and their membership in specific Reactome pathways. Presence in a specific pathway is indicated by a yellow box, whereas absence is indicated by a black box. The membership of the genes in spatial clusters 1 and 2, and their high expression in specific developmental stages (late infancy-adulthood and late prenatal-early childhood) have been indicated in a similar manner.

**
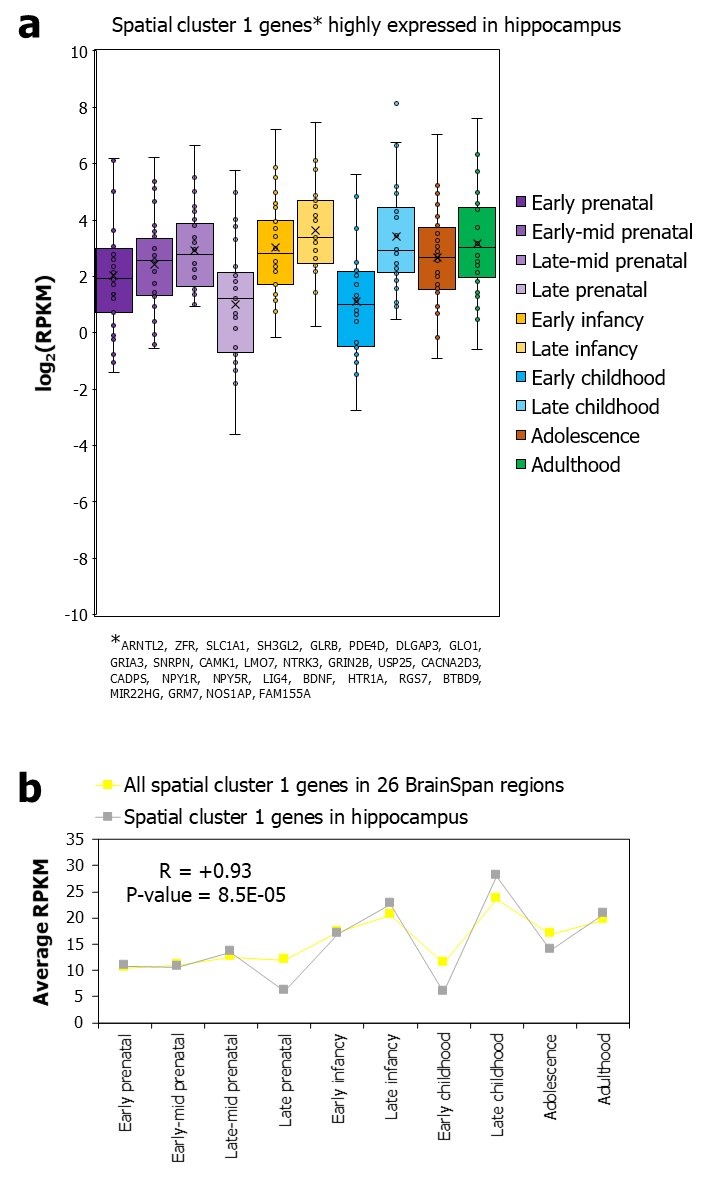
Supplementary Fig. 25 Temporal expression profiles of spatial cluster 1 genes in hippocampus. a** The box plots show the differences in the log_2_-transformed average expression (RPKM) values of 24 spatial cluster 1 genes in the hippocampal formation across ten developmental stages. In each box plot, the central line indicates the median, the cross mark indicates the mean, the bottom and top edges of the box indicate the interquartile range and the whiskers represent the maximum and minimum data points. **b** The figure shows the correlation of the average RPKM values of 24 spatial cluster 1 genes in the hippocampus and all the spatial cluster 1 genes in 26 BrainSpan structures across ten different developmental stages (horizontal axis). Pearson correlation coefficient (r) was used to compute the correlation between the average RPKM values at each stage.

**
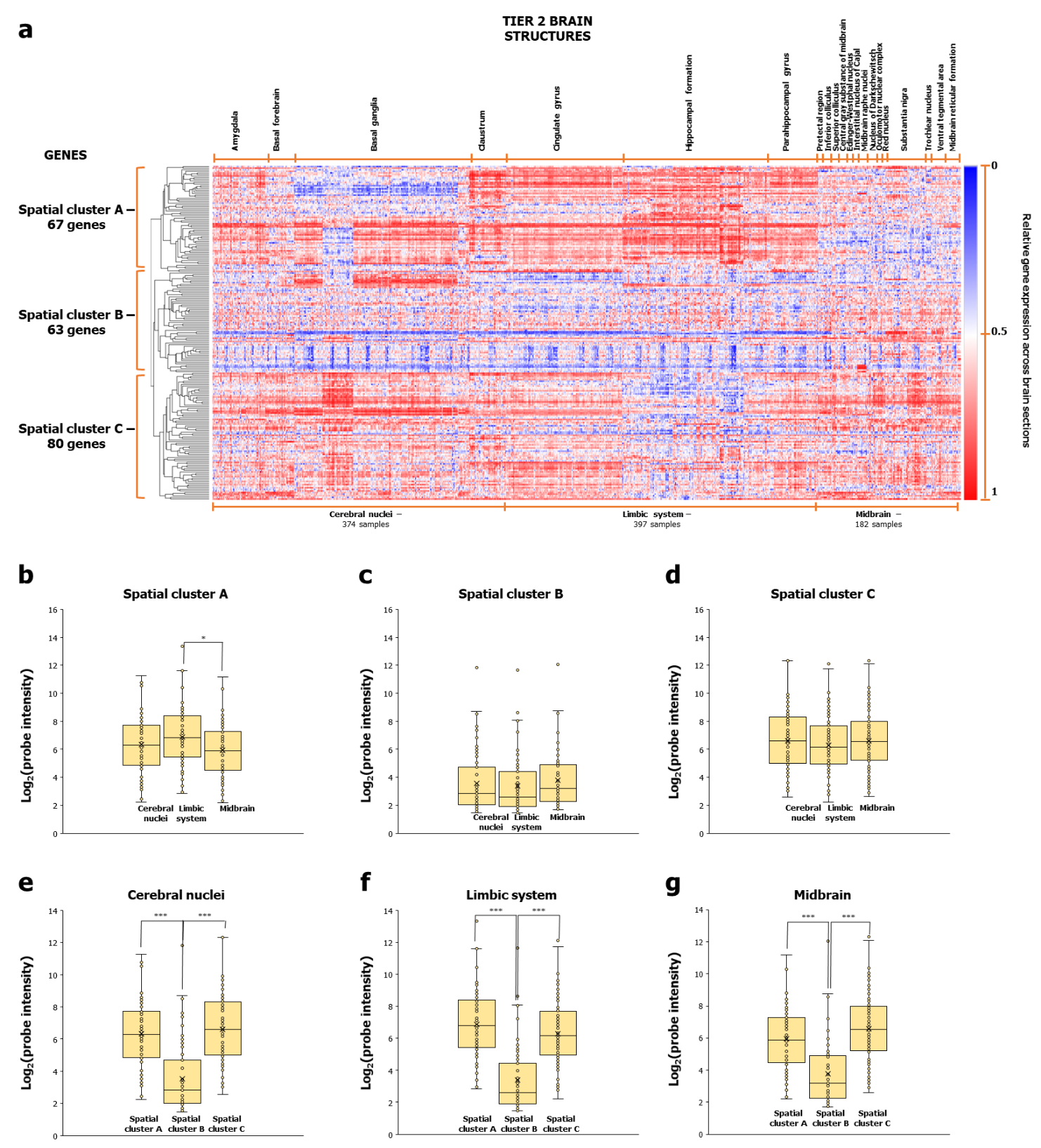
Supplementary Fig. 26: Trifurcation of AD-associated genes upon inclusion of PTSD genes. a** The figure shows the trichotomized expression of 210 AD (including PTSD)-associated genes across 374 cerebral nuclei, 397 limbic system and 182 midbrain samples in the Human Adult Microarray Data (Allen Brain Atlas). Relative gene expression of each of the genes across the 953 brain sections was hierarchically clustered by computing pairwise distances between the data points (log_2_-transformed probe intensities) using Pearson correlation and identifying closely linked clusters using the average linkage method. The clustered heat map was generated using the Morpheus software. The box plots show the differences in the log_2_-transformed average expression values of genes in **b** Spatial cluster A, **c** Spatial cluster B and **d** Spatial cluster C, across cerebral nuclei, limbic system and midbrain samples. In **e** – **g**, the box plots show the differences in **e** cerebral nuclei, **f** limbic system and **g** cerebral nuclei samples between spatial cluster A, spatial cluster B and spatial cluster C. The pairwise statistical significance of the difference between the means of the box plots derived from the Mann-Whitney U test has also been shown. *, ** and *** indicate p-value < 0.5, < 0.01 and < 0.001 respectively. In each box plot, the central line indicates the median, the bottom and top edges of the box indicate the interquartile range and the whiskers represent the maximum and minimum data points.

**
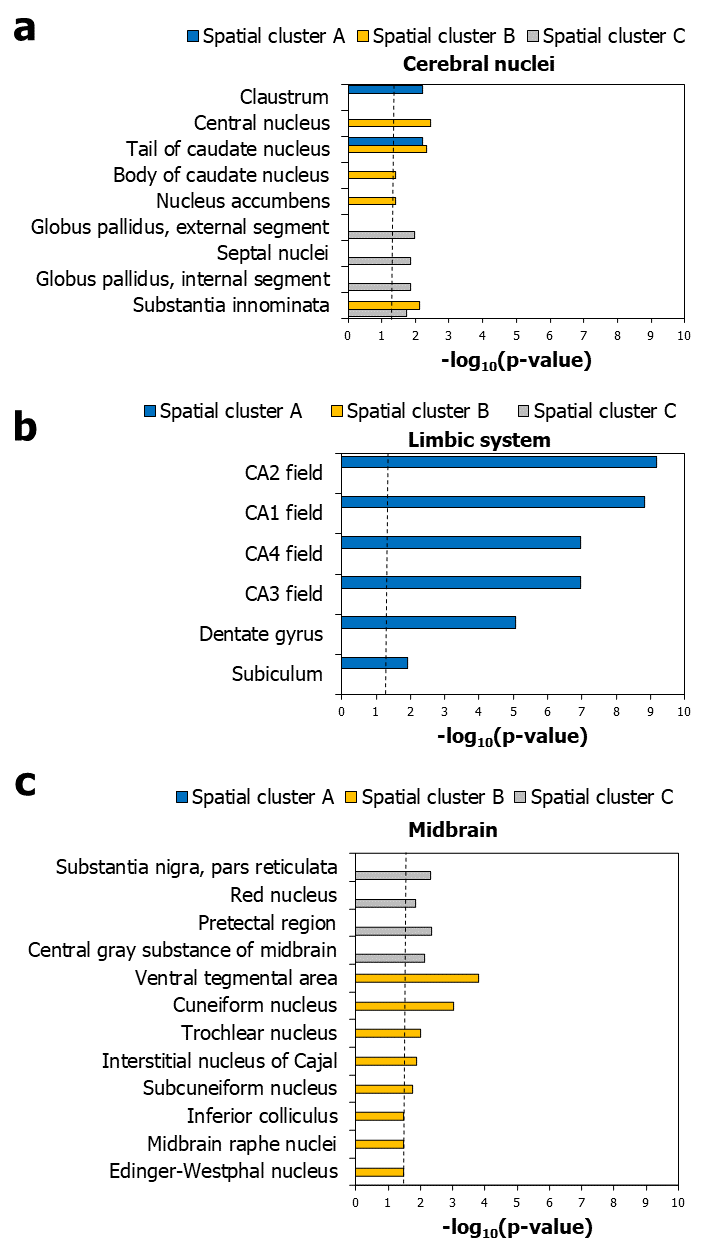
**

**Supplementary Fig. 27: Differential enrichment patterns of spatial clusters A-C in specific regions.** The figure shows the enrichment of spatial cluster A, spatial cluster B and spatial cluster C for genes showing relatively higher expression in **a** cerebral nuclei samples, **b** limbic systems samples and **c** midbrain samples, compared to other regions. The dotted black line in **a** – **c** indicates the cut-off value for –log_10_(p-value) after correction for multiple hypotheses using the Benjamini-Hochberg method (p-value < 0.05, log_10_(p-value) > 1.30103).

**
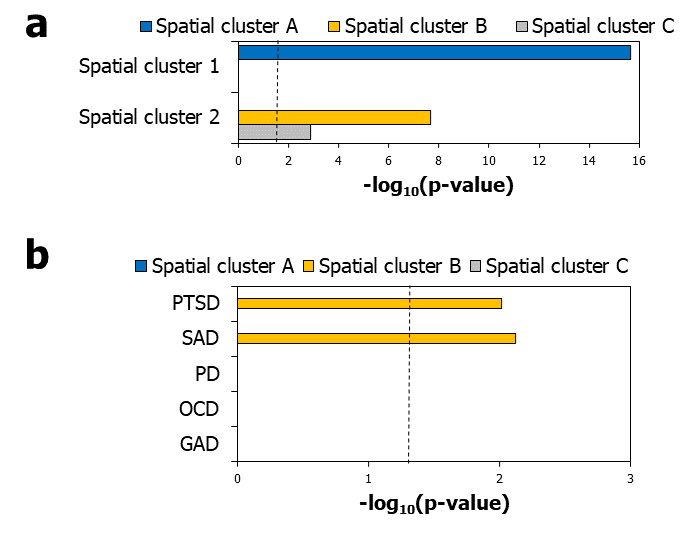
**

**Supplementary Fig. 28 The enrichment of spatial clusters 1, 2 and AD subtypes in spatial cluster A-C. a** The enrichment of spatial clusters A-C for genes in spatial cluster 1 and spatial cluster 2 (in terms of -log_10_(p-values)) has been shown. **b** The enrichment of spatial clusters A-C for genes associated with specific AD subtypes has been shown. The dotted black lines in **a** and **b** indicate the cut-off value for –log_10_(p-value) after correction for multiple hypotheses using the Benjamini-Hochberg method, i.e. 1.30103.

**
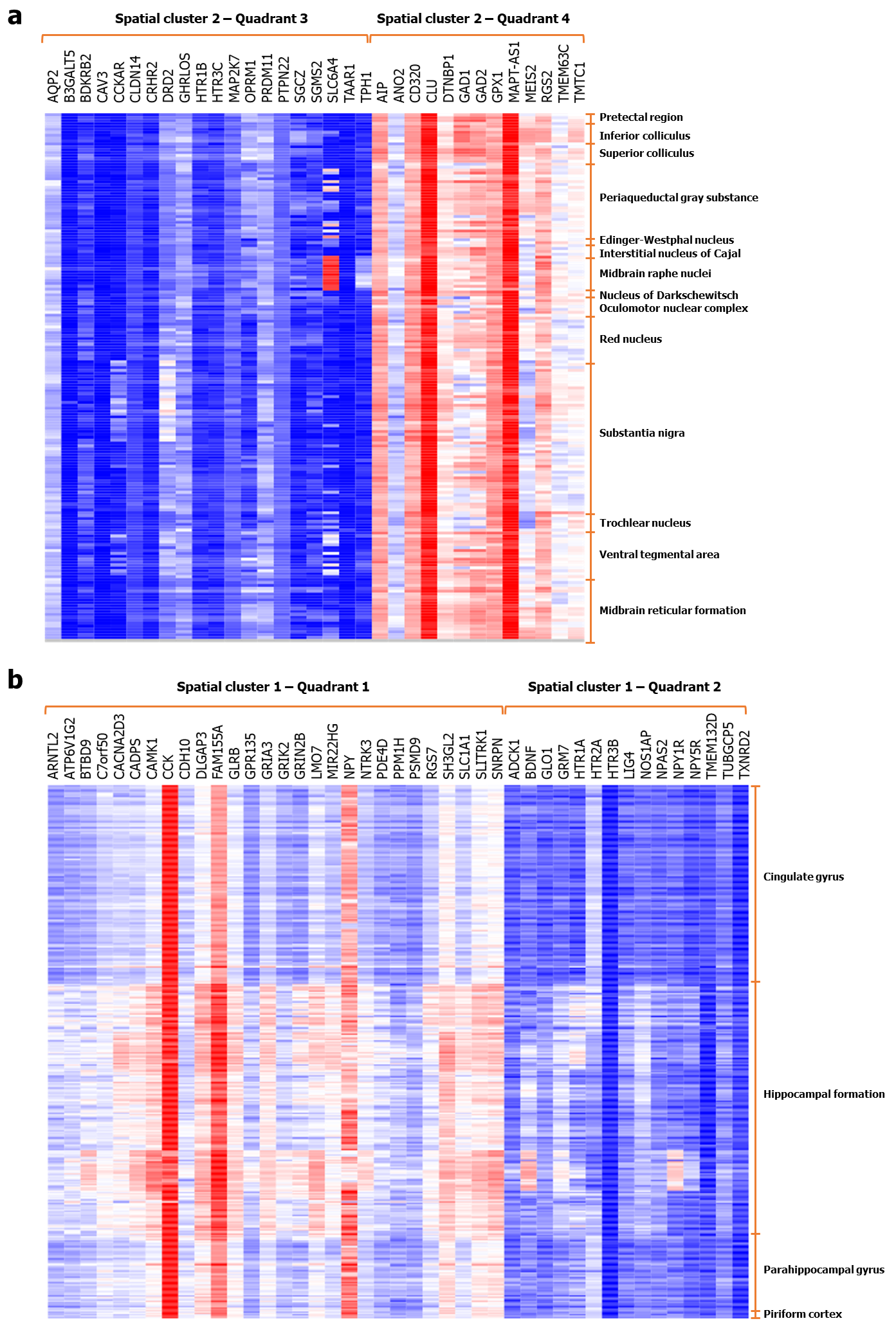
Supplementary Fig. 29: Subsets of spatial cluster 2 genes showed varying expression in midbrain areas. a** The figure shows the dichotomized expression of 33 spatial cluster 2 genes across samples from midbrain regions. Twenty spatial cluster 2 genes belonging to quadrant III showed lower expression than 13 spatial cluster 2 genes belonging to quadrant IV. **b** Spatial cluster 1 genes did not show similar expression variations across limbic samples. Relative gene expression of each of the genes across the brain sections was hierarchically clustered by computing pairwise distances between the data points (log_2_-transformed probe intensities) using Pearson correlation and identifying closely linked clusters using the average linkage method. The clustered heat map was generated using the Morpheus software.

**
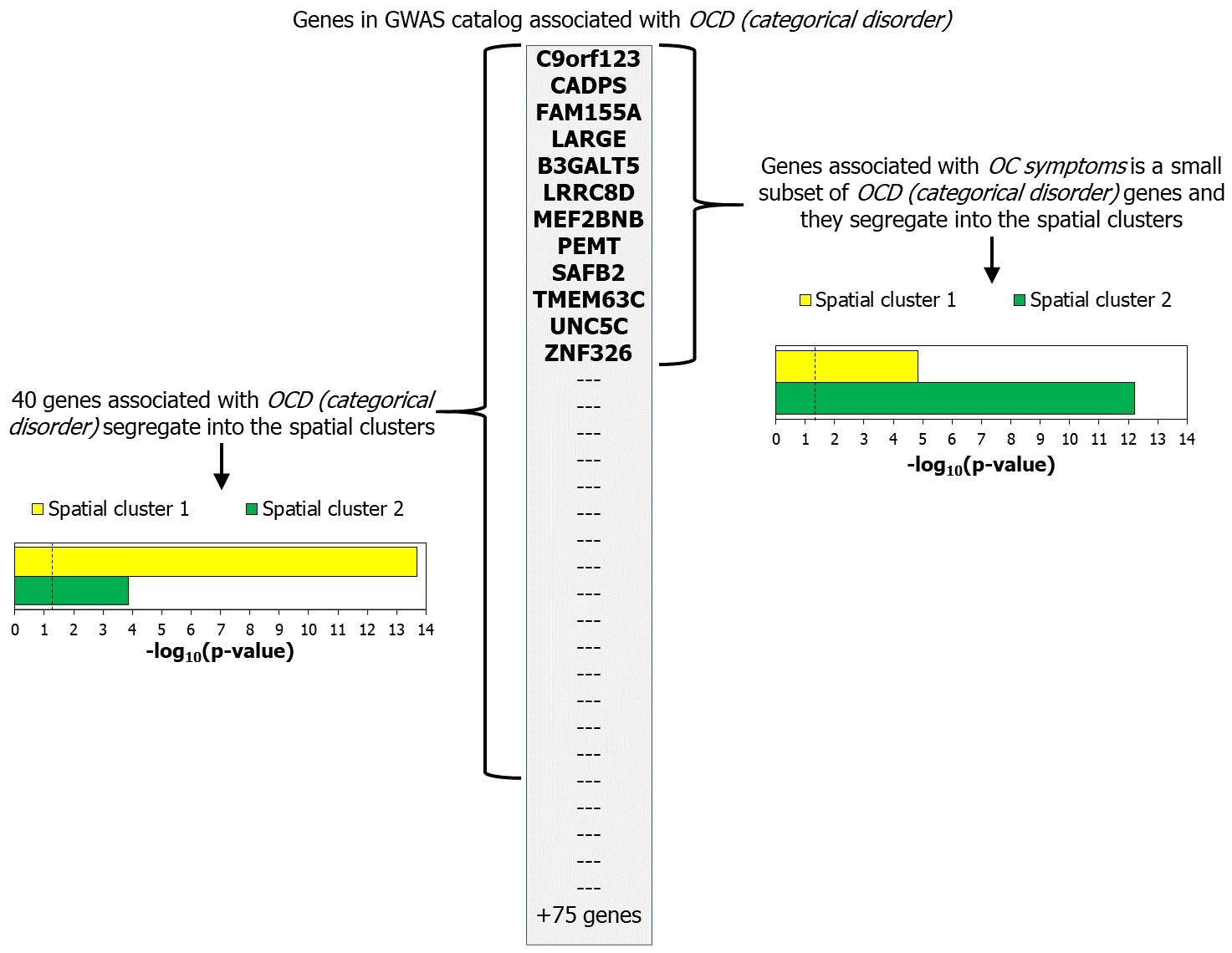
Supplementary Fig. 30: The enrichment of genes related to obsessive-compulsive symptoms in the spatial clusters.** This graphic illustrates the enrichment of genes linked to obsessive-compulsive (OC) symptoms in the two spatial clusters. It shows the small intersection of OCD (categorical disorder) genes with the genes associated with OC symptoms, and how the latter segregates preferentially into spatial cluster 2. On the other hand, OCD (categorical disorder) genes, exhibiting a larger intersection with the spatial clusters, segregate preferentially into spatial cluster 1.

**Supplementary Table 1: List of genes associated with the four anxiety disorder subtypes.** The table shows the genes associated with generalized anxiety disorder, social anxiety disorder, obsessive-compulsive disorder and panic disorder compiled from the DisGeNET database and the GWAS catalog. Note that all the genes in this list harbour or occur in the vicinity of genetic variants; i.e. sequence variation that is associated with the disease phenotype. However, note that there is no evidence to say that the variation causes the disease.

| **Anxiety disorder subtype** | **Number of genes** | **List of genes** |
| --- | --- | --- |
| Generalized anxiety disorder | 26 | PSMD9, HTR1B, BCL2, NPY, OPRM1, PTPN22, AIP, SLC6A4, NPSR1, DRD2, RGS2, COMT, CRHR1, BDNF, HTR2A, THBS2, LOC105377864, LOC107986777, AP4B1-AS1, LOC105371720, NPSR1-AS1, MIR4761, MAPT-AS1, BDNF-AS, LOC101929523, LINC02210-CRHR1 |
| Social anxiety disorder | 9 | ARNTL2, SLC6A4, RGS2, CAV3, SLC2A9, DRD2, ARNTL2-AS1, LOC105371720, OXTR |
| Panic disorder | 92 | SGCZ, PEPD, PDE4B, GPX1, OGG1, LOC105371665, ASIC1, TPH2, BDNF, TMEM132D, MANEA, NPY1R, TPH1, C7orf50, RGS7, NPY5R, LOC107985174, ANO2, LIG4, SLC6A4, SMAD1, PRDM11, GLRB, CRHR1, EPHX2, CCK, CCKAR, SLC6A2, FOCAD, GAD1, HCRTR1, HTR2A, ZFR, DTNBP1, GABRA6, FAAH, LINC00461, NPSR1, SDK2, LINC02210-CRHR1, RGS2, COMT, GHRL, CRHR2, GLO1, TMEM132E, MAP2K7, ADORA2A, HTR1A, MIR22HG, SNAPC2, TLCD2, PKP1, C17orf102, LOC101927284, CALCOCO1, BDKRB2, CAMK1, BDNF-AS, MAPT-AS1, HTR2A-AS1, MIR579, NPSR1-AS1, MIR4761, GHRLOS, ADORA2A-AS1, MIR22, SPECC1L-ADORA2A, TNFRSF21, PLEKHG1, TMEM16B, CLU, GRM7, USP25, SMOX, SUSD1, SNRPN, PTBP3, SNURF, FAM174A - RN7SKP62, RAN - Y_RNA, GULOP - CLU, RAD23BP3 - USP25, SOX21-AS1 , LINC02450 , KCNA3 - Y_RNA, LINC01500, SMOX - LINC01433, CPT1C - TSKS, PTMAP6 - RNU6-929P, SNHG14, DHFRP5 |
| Obsessive-compulsive disorder | 147 | HTR3D, OLIG2, LOC105370269, TAFA2, CDH2, DLGAP3, HTR3B, TRPM7, MTHFR, HTR3E, SLC1A1, CAV3, GRIK2, NOS1AP, SLITRK1, BDNF-AS, ARVCF, SLC6A4, GPR135, OPRM1, HTR1A, TNF, GRIN2B, PDE4D, LOC105377864, ATP6V1G2, COMT, ADCK1, NTRK2, NTRK3, MOG, BDNF, LINC02395, SPATA6L, NPSR1, LOC112267857, GRN, NECTIN1, TPH2, MEIS2, GRIA3, POU1F1, TPH1, HTR1B, HTR2A, HTR3C, CARMIL1, GAD2, BTBD9, LOC105374249, LOC107985505, OXTR, NFKBIL1, TXNRD2, LOC105371720, NPSR1-AS1, MIR4761, HTR3E-AS1, ATP6V1G2-DDX39B, PTPRD, CDH10, MEF2BNB, LOC729056, UNC5C, LOC100420, CADPS, LARGE, FAM155A, C9orf123, PEMT, SAFB2, LRRC8D, TMEM63C, MARK2P15, RPS16P2, LOC107985997, CACNA2D3, LOC100421611, LOC284930, CLEC5A, TAAR1, TMTC1, MIR3683, ATP13A4, LMO7, GRID2, CD320, TUBGCP5, RP11-324E6.6, FAIM2, DACH1, HHAT, NEK11, RP11-467J12.2, MUC22, SGMS2, TMEM45B, SH3GL2, NPAS2, LOC101928096, AL136524.1, CLDN14, AC126763.1, PPM1H, PDE4B, LOC646114, MSNL1, B3GALT5, OR7H2P, TPRD, ZNF326, HMGN2P8, AQP2, HACE1, MZT1, LOC100049717, RP11-467J12.3, BORCS8, JCADP1 - B3GALT5, LARGE1, DMAC1 , LRRC8D - RN7SKP272, MARK2P15 , LAPTM4A-DT - RPS16P2, SLC16A14 - SP110, TAAR2 - TAAR1, TMTC1 , NHLRC2 - ADRB1, LINC00702, TUSC3 - PPM1AP1, RPL21P17, S100A7 - RN7SL44P, SLCO1C1 - SLCO1B3, Y_RNA, HCAR2 - HCAR3, RPL31P35 - SEC61G, CHCHD2P3 - LSM14A, ZNF804B - FLNC, LINC01987, SNORA70 - LINC01526, R3HDM2P2, PHBP21 - CHD9, TRMT11, MIR3180-4, MEF2B, MGAM, BORCS8-MEF2B |

**Supplementary Table 2: List of genes associated with post-traumatic stress disorder.** The table shows the genes associated with post-traumatic stress disorder compiled from the DisGeNET database and the GWAS catalog. Note that all the genes in this list harbour or occur in the vicinity of genetic variants; i.e. sequence variation that is associated with the disease phenotype. However, note that there is no evidence to say that the variation causes the disease.

| **Anxiety disorder subtype** | **Number of genes** | **List of genes** |
| --- | --- | --- |
| Post-traumatic stress disorder | 141 | CYRIB, ADCY8, TLL1, RORA, PRTFDC1, ANKRD55, PTPRVP, LHX2, TBC1D2, BDNF-AS, FKBP5, ADCYAP1R1, ANKK1, ADRB2, SLC18A2, SKA2, CRHBP, DICER1, ALOX12, CNR1, NOS1, CRP, IL1B, ANK3, MBP, CHRNA5, LINC02210-CRHR1, FLT4, WWC1, PPM1F, IL6-AS1, STMN1, CACNA1C, MIR6795, ESR1, DRD3, LOC107986458, SLC6A3, TERT, NPSR1-AS1, SNCA, LOC101929309, C5orf67, NR3C1, ACE, SPRY4-AS1, SMG6, GHSR, SRD5A2, LAMC2, VWDE, LOC105375743, LOC107984425, PCSK9, KL, MIR4761, LOC112267956, LOC105371720, ALOX12-AS1, LOC105369501, IL6, LOC105376885, NOTCH3, SH3RF2, SRR, LOC107986777, LINC00964, DLG2, STEAP1B, AK092087, UNC13C, SDC2, SIPA1L2, KAT2B, DPP6, KIAA1456, MIR3166, MEX3B, COBL, OTOR, NDRG1, GABBR2, MTRNR2L5, AKT3, TECRL, TRPS1, SLC4A5, RPS29, FAM49B, ZMAT3, PRKCA, GRIN1, POGK, PTPRV, LGR6, UST, CSMD1, LOC101928174, LINC01499, LOC285629 - LOC105377693, LOC105375523, LOC105370213 - LOC107984565, LOC102723778 - LOC105374564, LOC101929902, MIR3166 - CTSC, LOC102724001, LOC105375278 - LOC107986738, OTOR - LOC105372543, LOC100419872 - ZWINT, AKT3 - LOC339529, DPP3P1 - MTCO3P27, LOC100420746 - CARSP2, LOC105378178, GRID1, CNN2P10 - POGK, PTPRVP - LGR6, LOC105376267 - LOC100505588, BDNF, COMT, TPH2, SLC6A4, OXTR, HTR1A, CRHR1, NTRK2, DRD2, CCK, TNF, TPH1, CAV3, CRHR2, HCRTR1, FAAH, GAD1, NOS1AP, RGS2, MAPT-AS1, NPY, GHRL, NPSR1, GHRLOS |

**Supplementary Table 3: Major areas enriched among PTSD-associated genes.** The number of tier 3 structures in each of the thirteen tier 1 structures that show a statistically significant enrichment of **(a)** PTSD-associated gene set and **(b)** a single AD-associated gene set (i.e., combining PTSD, OCD, PD, GAD and SAD associated gene sets) at p-value < 0.05, after correction for multiple hypotheses using the Benjamini-Hochberg method. The number of enriched tier 3 structures has been shown in each cell, in comparison with the total number of tier 3 structures mapped to the specific tier 1 structure.

| **Level 1 brain structure** | **Number of tier 3 brain structures showing significant association** | |
| --- | --- | --- |
|  | **a** | **b** |
| Cerebellum | 0/38 | 0/38 |
| Cerebral nuclei | **6**/21 | **4**/21 |
| Diencephalon | 2/35 | 0/35 |
| Frontal lobe | 0/28 | 0/28 |
| Insular cortex | 0/2 | 0/2 |
| Limbic system | **3**/16 | 0/16 |
| Medulla oblongata | 0/14 | 0/14 |
| Midbrain | **2**/17 | **2**/17 |
| Occipital lobe | 0/13 | 0/13 |
| Parietal lobe | 0/14 | 0/14 |
| Pons | 0/14 | 1/14 |
| Temporal lobe | 0/17 | 0/17 |
| White matter | 0/2 | 0/2 |

**Supplementary References**

1. Björkegren JL, Kovacic JC, Dudley JT, Schadt EE. Genome-wide significant loci: how important are they? Systems genetics to understand heritability of coronary artery disease and other common complex disorders. *Journal of the American College of Cardiology* 2015; **65**(8)**:** 830-845.

2. Mufford MS, van der Meer D, Andreassen OA, Ramesar R, Stein DJ, Dalvie S. A review of systems biology research of anxiety disorders. *Brazilian Journal of Psychiatry* 2020; **43:** 414-423.

3. Su X, Li W, Lv L, Li X, Yang J, Luo X-J *et al.* Transcriptome-Wide Association Study Provides Insights Into the Genetic Component of Gene Expression in Anxiety. *Frontiers in genetics* 2021**:** 1901.

4. Faravelli C, Sauro CL, Godini L, Lelli L, Benni L, Pietrini F *et al.* Childhood stressful events, HPA axis and anxiety disorders. *World journal of psychiatry* 2012; **2**(1)**:** 13.

5. Holland SM. Principal components analysis (PCA). *Department of Geology, University of Georgia, Athens, GA* 2008**:** 30602-32501.

6. Barabási A-L, Gulbahce N, Loscalzo J. Network medicine: a network-based approach to human disease. *Nature reviews genetics* 2011; **12**(1)**:** 56-68.

7. Griffith M, Griffith OL, Coffman AC, Weible JV, McMichael JF, Spies NC *et al.* DGIdb: mining the druggable genome. *Nature methods* 2013; **10**(12)**:** 1209-1210.

8. Wishart DS, Knox C, Guo AC, Cheng D, Shrivastava S, Tzur D *et al.* DrugBank: a knowledgebase for drugs, drug actions and drug targets. *Nucleic acids research* 2008; **36**(Database issue)**:** D901-D906.

9. Milardi D, Bramanti P, Milazzo C, Finocchio G, Arrigo A, Santoro G *et al.* Cortical and subcortical connections of the human claustrum revealed in vivo by constrained spherical deconvolution tractography. *Cerebral Cortex* 2015; **25**(2)**:** 406-414.

10. Amaral DG, Cowan WM. Subcortical afferents to the hippocampal formation in the monkey. *Journal of Comparative Neurology* 1980; **189**(4)**:** 573-591.

11. Menzies L, Chamberlain SR, Laird AR, Thelen SM, Sahakian BJ, Bullmore ET. Integrating evidence from neuroimaging and neuropsychological studies of obsessive-compulsive disorder: the orbitofronto-striatal model revisited. *Neuroscience & Biobehavioral Reviews* 2008; **32**(3)**:** 525-549.

12. Yu X-D, Zhu Y, Sun Q-X, Deng F, Wan J, Zheng D *et al.* Distinct serotonergic pathways to the amygdala underlie separate behavioral features of anxiety. *Nature Neuroscience* 2022; **25**(12)**:** 1651-1663.

13. Pitts MW, Todorovic C, Blank T, Takahashi LK. The central nucleus of the amygdala and corticotropin-releasing factor: insights into contextual fear memory. *Journal of Neuroscience* 2009; **29**(22)**:** 7379-7388.

14. Azmitia EC, Segal M. An autoradiographic analysis of the differential ascending projections of the dorsal and median raphe nuclei in the rat. *Journal of Comparative Neurology* 1978; **179**(3)**:** 641-667.

15. Fuxe K. Evidence for the existence of monoamine neurons in the central nervous system. IV The distribution of monoamine terminals in the central nervous system. *Acta physioL scand* 1965; **247:** 39-85.

16. Di Matteo V, Pierucci M, Esposito E, Crescimanno G, Benigno A, Di Giovanni G. Serotonin modulation of the basal ganglia circuitry: therapeutic implication for Parkinson's disease and other motor disorders. *Progress in brain research* 2008; **172:** 423-463.

17. Dorocic IP, Fürth D, Xuan Y, Johansson Y, Pozzi L, Silberberg G *et al.* A whole-brain atlas of inputs to serotonergic neurons of the dorsal and median raphe nuclei. *Neuron* 2014; **83**(3)**:** 663-678.

18. Rapee RM, Sanderson WC, McCauley PA, Di Nardo PA. Differences in reported symptom profile between panic disorder and other DSM-III-R anxiety disorders. *Behaviour Research and Therapy* 1992; **30**(1)**:** 45-52.

19. Baldwin DS, Anderson IM, Nutt DJ, Allgulander C, Bandelow B, den Boer JA *et al.* Evidence-based pharmacological treatment of anxiety disorders, post-traumatic stress disorder and obsessive-compulsive disorder: a revision of the 2005 guidelines from the British Association for Psychopharmacology. *Journal of Psychopharmacology* 2014; **28**(5)**:** 403-439.

20. Winkelmann J, Prager M, Lieb R, Pfister H, Spiegel B, Wittchen H-U *et al.* Anxietas tibiarum. *Journal of neurology* 2005; **252**(1)**:** 67-71.

21. Bolhuis K, McAdams T, Monzani B, Gregory AM, Mataix-Cols D, Stringaris A *et al.* Aetiological overlap between obsessive–compulsive and depressive symptoms: a longitudinal twin study in adolescents and adults. *Psychological Medicine* 2014; **44**(7)**:** 1439-1449.

22. Van Oppen P, Hoekstra RJ, Emmelkamp PM. The structure of obsessive-compulsive symptoms. *Behaviour research and therapy* 1995; **33**(1)**:** 15-23.

23. Offidani E, Guidi J, Tomba E, Fava GA. Efficacy and tolerability of benzodiazepines versus antidepressants in anxiety disorders: a systematic review and meta-analysis. *Psychotherapy and psychosomatics* 2013; **82**(6)**:** 355-362.

24. Garakani A, Murrough JW, Freire RC, Thom RP, Larkin K, Buono FD *et al.* Pharmacotherapy of anxiety disorders: current and emerging treatment options. *Frontiers in psychiatry* 2020**:** 1412.
