# Supplementary material for: Spatiotemporal expression patterns of anxiety disorder-associated genes
Source: Transl Psychiatry. 2023 Dec 13;13:385. doi: 10.1038/s41398-023-02693-y (PMC10719387; doi:10.1038/s41398-023-02693-y)
Supplement: Supplementary file 2 — Supplementary Data Files [file 41398_2023_2693_MOESM2_ESM.pdf]

Supplementary Data Files

Spatiotemporal expression patterns of anxiety disorder-associated genes

This file contains:

- Supplementary Data Files 1 and 2

Supplementary Data File 1: Summary of AD-associated SNP associations with proximal genes in the FIVEx eQTL database. The table includes information on the statistical significance -$\log_{10}P$ of SNP-gene association (the likelihood of impact of variant on gene expression), effect size (magnitude of the impact of the SNP on gene expression), standard error (or SE, i.e. uncertainty in the effect size estimate), posterior inclusion probability (or 'PIP', the likelihood of a strong causal relationship between the SNP and the gene, considering the joint influence of all SNPs in linkage disequilibrium contributing to the observed expression change). Note that while only five SNP-gene associations exhibit a PIP value less than 0.05, signifying potential causal relationships, the remaining SNPs nevertheless significantly influence gene expression, with a p-value below 0.05.

**SNP ID**	**Gene**	**eQTL study**	**Tissue**	**$-\log_{10}P$**	**Effect size**	**SE**	**PIP**
rs1059004	OLIG2	BrainSeq	brain	1.19	-0.063	0.034	-
rs1148374	CDH2	GTEx	brain_anterior_cingulate_cortex	2.32	-0.084	0.029	-
rs1148374	CDH2	GTEx	brain_caudate	1.91	-0.068	0.027	-
rs1148374	CDH2	GTEx	brain_cortex	1.38	-0.059	0.029	-
rs11583978	DLGAP3	GTEx	brain_cerebellar_hemisphere	1.8	-0.099	0.04	-
rs12682807	SLC1A1	GTEx	brain_spinal_cord	1.72	-0.47	0.19	-
rs12682807	SPATA6L	GTEx	brain_hypothalamus	1.53	-0.28	0.13	-
rs13316193	CAV3	GTEx	brain_putamen	1.58	-0.29	0.13	-
rs13316193	OXTR	ROSMAP	brain_naive	2	-0.13	0.052	-
rs13316193	OXTR	GTEx	brain_cortex	1.5	-0.2	0.093	-
rs13316193	OXTR	BrainSeq	brain	1.47	-0.1	0.048	-
rs1519480	BDNF-AS	GTEx	brain_anterior_cingulate_cortex	2.52	0.11	0.035	-
rs165599	COMT	GTEx	brain_cerebellar_hemisphere	2.31	0.091	0.031	-
rs165599	COMT	BrainSeq	brain	1.74	0.029	0.012	-
rs165599	COMT	GTEx	brain_hypothalamus	1.35	0.073	0.036	-
rs1755715	GPR135	BrainSeq	brain	11.53	0.13	0.018	-
rs1755715	GPR135	ROSMAP	brain_naive	8.44	0.092	0.015	0.0025
rs1755715	GPR135	GTEx	brain_cerebellum	4.54	0.12	0.028	-
rs1755715	GPR135	GTEx	brain_putamen	3.63	0.16	0.042	-
rs1755715	GPR135	GTEx	brain_anterior_cingulate_cortex	2.77	0.16	0.049	-
rs1755715	GPR135	GTEx	brain_cortex	2.62	0.1	0.032	-
rs1755715	GPR135	GTEx	brain_caudate	2.61	0.13	0.041	-
rs1755715	GPR135	GTEx	brain_cerebellar_hemisphere	2.41	0.068	0.023	-
rs1755715	GPR135	GTEx	brain_frontal_cortex	2.03	0.11	0.041	-
rs1755715	GPR135	GTEx	brain_hypothalamus	1.95	0.13	0.048	-
rs1799971	OPRM1	GTEx	brain_cerebellum	3.56	0.28	0.076	-
rs1799971	OPRM1	GTEx	brain_cerebellar_hemisphere	2.77	0.33	0.1	-
rs1799971	OPRM1	GTEx	brain_amygdala	1.68	0.55	0.23	-
rs1799972	OPRM1	GTEx	brain_caudate	1.52	-1.06	0.48	-
rs1799972	OPRM1	GTEx	brain_amygdala	1.44	1.37	0.64	-
rs2071592	ATP6V1G2	ROSMAP	brain_naive	4.51	-0.049	0.012	-
rs2071592	ATP6V1G2	BrainSeq	brain	3.62	-0.045	0.012	-
rs2071592	ATP6V1G2	GTEx	brain_frontal_cortex	1.41	-0.082	0.039	-
rs2071592	NFKBIL1	GTEx	brain_substantia_nigra	1.68	-0.08	0.033	-
rs2071592	NFKBIL1	BrainSeq	brain	1.64	-0.025	0.011	-
rs2071592	NFKBIL1	GTEx	brain_putamen	1.36	-0.063	0.031	-
rs2228622	SLC1A1	GTEx	brain_putamen	1.79	0.15	0.06	-

rs2228622	SLC1A1	GTEx	brain_cerebellum	1.45	0.074	0.035	-
rs2254298	CAV3	GTEx	brain_nucleus_accumbens	1.83	-0.41	0.17	-
rs2268490	CAV3	GTEx	brain_nucleus_accumbens	1.35	-0.3	0.14	-
rs2268490	OXTR	GTEx	brain_cerebellum	1.72	-0.13	0.056	-
rs2268493	CAV3	GTEx	brain_putamen	2.26	-0.38	0.13	-
rs2268493	OXTR	BrainSeq	brain	1.31	-0.11	0.055	-
rs2364841	ADCK1	BrainSeq	brain	1.32	-0.057	0.029	-
rs237887	CAV3	GTEx	brain_putamen	1.39	-0.26	0.12	-
rs237887	OXTR	ROSMAP	brain_naive	6.15	-0.27	0.053	-
rs237887	OXTR	BrainSeq	brain	3.55	-0.18	0.048	-
rs237887	OXTR	GTEx	brain_caudate	1.3	-0.36	0.18	-
rs2857766	MOG	BrainSeq	brain	1.44	0.1	0.049	-
rs2883187	BDNF	GTEx	brain_cerebellum	1.41	0.11	0.052	-
rs2883187	BDNF	GTEx	brain_substantia_nigra	1.33	0.32	0.16	-
rs301430	SLC1A1	GTEx	brain_spinal_cord	3.22	-0.38	0.1	-
rs301430	SLC1A1	GTEx	brain_nucleus_accumbens	1.71	-0.13	0.053	-
rs301430	SLC1A1	GTEx	brain_hippocampus	1.3	-0.13	0.067	-
rs301435	SLC1A1	ROSMAP	brain_naive	1.45	-0.031	0.015	-
rs301435	SPATA6L	ROSMAP	brain_naive	1.68	-0.049	0.021	-
rs301979	SLC1A1	GTEx	brain_substantia_nigra	2.98	-0.33	0.095	-
rs301979	SPATA6L	BrainSeq	brain	1.43	0.041	0.019	-
rs3087879	SPATA6L	GTEx	brain_nucleus_accumbens	1.43	-0.14	0.068	-
rs3758987	HTR3B	BrainSeq	brain	2.36	0.09	0.031	-
rs3758987	HTR3B	ROSMAP	brain_naive	1.38	0.054	0.027	-
rs3780412	SLC1A1	GTEx	brain_putamen	1.79	0.15	0.06	-
rs3780412	SLC1A1	GTEx	brain_substantia_nigra	1.5	-0.22	0.1	-
rs3780413	SLC1A1	GTEx	brain_substantia_nigra	2.45	-0.31	0.1	-
rs3785817	GRN	ROSMAP	brain_naive	2.07	-0.041	0.016	-
rs4565946	TPH2	BrainSeq	brain	2.88	0.13	0.039	-
rs4652867	DLGAP3	GTEx	brain_cerebellar_hemisphere	1.39	0.097	0.047	-
rs4686301	CAV3	GTEx	brain_putamen	1.44	-0.28	0.13	-
rs4988462	POU1F1	GTEx	brain_hypothalamus	1.82	-0.37	0.15	-
rs4988462	POU1F1	GTEx	brain_hippocampus	1.33	-0.31	0.15	-
rs6265	BDNF-AS	GTEx	brain_frontal_cortex	3.28	-0.15	0.043	-
rs6265	BDNF-AS	GTEx	brain_spinal_cord	1.94	-0.12	0.046	-
rs6265	BDNF-AS	ROSMAP	brain_naive	1.84	-0.041	0.017	-
rs6265	BDNF-AS	GTEx	brain_putamen	1.81	-0.11	0.045	-
rs6265	BDNF	GTEx	brain_cerebellar_hemisphere	1.66	-0.19	0.08	-
rs6296	HTR1B	GTEx	brain_anterior_cingulate_cortex	1.43	-0.42	0.2	-
rs6296	HTR1B	GTEx	brain_cortex	1.38	-0.28	0.14	-
rs6517137	OLIG2	GTEx	brain_cerebellar_hemisphere	3	-0.34	0.1	-
rs6662980	DLGAP3	GTEx	brain_cerebellar_hemisphere	2.02	-0.1	0.039	-
rs7541937	DLGAP3	GTEx	brain_cerebellar_hemisphere	1.42	-0.08	0.038	-
rs7858819	SLC1A1	GTEx	brain_spinal_cord	2.71	-0.35	0.11	-
rs7858819	SLC1A1	GTEx	brain_nucleus_accumbens	1.67	-0.14	0.058	-
rs7858819	SLC1A1	GTEx	brain_hippocampus	1.53	-0.16	0.071	-
rs10415555	PEPD	GTEx	brain_cerebellum	1.85	0.082	0.033	-
rs10415555	PEPD	BrainSeq	brain	1.68	0.037	0.016	-
rs10415555	PEPD	GTEx	brain_cerebellar_hemisphere	1.61	0.074	0.032	-
rs1052133	OGG1	GTEx	brain_cerebellum	1.79	0.046	0.019	-
rs1052133	CAMK1	GTEx	brain_cerebellum	1.34	0.062	0.031	-

rs10875995	ASIC1	ROSMAP	brain_naive	3.29	-0.054	0.015	-
rs10875995	ASIC1	GTEx	brain_spinal_cord	2.38	-0.21	0.07	-
rs10879357	TPH2	BrainSeq	brain	1.66	0.078	0.034	-
rs11030104	BDNF-AS	GTEx	brain_putamen	2.44	-0.13	0.045	-
rs11030104	BDNF-AS	GTEx	brain_frontal_cortex	2.34	-0.13	0.043	-
rs11030104	BDNF-AS	ROSMAP	brain_naive	1.9	-0.041	0.016	-
rs11030104	BDNF	GTEx	brain_cerebellar_hemisphere	1.9	-0.2	0.079	-
rs11030104	BDNF	ROSMAP	brain_naive	1.44	-0.11	0.052	-
rs11030104	BDNF-AS	GTEx	brain_spinal_cord	1.38	-0.099	0.047	-
rs11724320	NPY1R	GTEx	brain_amygdala	1.34	-0.2	0.1	-
rs11724320	NPY5R	GTEx	brain_caudate	1.72	0.11	0.047	-
rs11763020	C7orf50	BrainSeq	brain	8.28	0.14	0.023	-
rs11763020	C7orf50	GTEx	brain_frontal_cortex	7.65	0.21	0.035	0.0045
rs11763020	C7orf50	GTEx	brain_hippocampus	6.09	0.29	0.053	-
rs11763020	C7orf50	ROSMAP	brain_naive	5.96	0.096	0.02	-
rs11763020	C7orf50	GTEx	brain_cortex	5.55	0.2	0.041	-
rs11763020	C7orf50	GTEx	brain_caudate	4.53	0.19	0.043	-
rs11763020	C7orf50	GTEx	brain_substantia_nigra	4.26	0.33	0.075	-
rs11763020	C7orf50	GTEx	brain_nucleus_accumbens	3.4	0.2	0.054	-
rs11763020	C7orf50	GTEx	brain_hypothalamus	3.09	0.17	0.05	-
rs11763020	C7orf50	GTEx	brain_putamen	2.74	0.17	0.052	-
rs11763020	C7orf50	GTEx	brain_anterior_cingulate_cortex	2.28	0.19	0.065	-
rs11763020	C7orf50	GTEx	brain_amygdala	1.95	0.16	0.061	-
rs11946004	NPY5R	GTEx	brain_anterior_cingulate_cortex	1.94	-0.23	0.09	-
rs1386483	TPH2	BrainSeq	brain	2.11	0.093	0.035	-
rs173365	CRHR1	ROSMAP	brain_naive	7.05	-0.15	0.028	-
rs173365	CRHR1	GTEx	brain_amygdala	2.14	0.45	0.16	-
rs173365	MAPT-AS1	ROSMAP	brain_naive	2.77	0.075	0.024	-
rs173365	MAPT-AS1	GTEx	brain_amygdala	1.99	0.15	0.058	-
rs173365	MAPT-AS1	GTEx	brain_frontal_cortex	1.68	-0.1	0.043	-
rs17689918	CRHR1	ROSMAP	brain_naive	15.8	0.28	0.033	0.00022
rs17689918	CRHR1	GTEx	brain_amygdala	3	-0.64	0.18	-
rs17689918	CRHR1	GTEx	brain_nucleus_accumbens	1.36	-0.21	0.1	-
rs17689918	MAPT-AS1	ROSMAP	brain_naive	16.43	-0.24	0.027	0.00045
rs17689918	MAPT-AS1	GTEx	brain_nucleus_accumbens	2.82	-0.23	0.071	-
rs17689918	MAPT-AS1	GTEx	brain_amygdala	2.59	-0.21	0.066	-
rs17689918	MAPT-AS1	GTEx	brain_frontal_cortex	2.08	0.14	0.051	-
rs17689918	MAPT-AS1	GTEx	brain_spinal_cord	1.93	-0.34	0.13	-
rs17689918	MAPT-AS1	GTEx	brain_cortex	1.81	0.12	0.048	-
rs1799923	CCK	GTEx	brain_spinal_cord	1.58	0.58	0.25	-
rs1799923	CCK	GTEx	brain_nucleus_accumbens	1.34	-0.33	0.16	-
rs1800532	TPH1	BrainSeq	brain	1.86	-0.091	0.037	-
rs1800855	CCKAR	BrainSeq	brain	6.55	-0.21	0.04	-
rs1800857	CCKAR	BrainSeq	brain	3.76	-0.19	0.051	-
rs2039391	FOCAD	BrainSeq	brain	1.61	-0.043	0.019	-
rs242924	CRHR1	ROSMAP	brain_naive	5.3	-0.13	0.028	-
rs242924	CRHR1	GTEx	brain_amygdala	1.52	0.36	0.16	-
rs242924	MAPT-AS1	GTEx	brain_amygdala	1.84	0.14	0.056	-
rs242924	MAPT-AS1	BrainSeq	brain	1.33	-0.036	0.018	-
rs2770292	HTR2A	GTEx	brain_anterior_cingulate_cortex	1.86	-0.21	0.083	-

rs2770292	HTR2A	GTEx	brain_caudate	1.86	0.33	0.13	-
rs2910931	ZFR	GTEx	brain_substantia_nigra	3.18	-0.091	0.025	-
rs3213207	DTNBP1	ROSMAP	brain_naive	6.33	0.096	0.019	-
rs3213207	DTNBP1	GTEx	brain_caudate	3.53	0.33	0.087	-
rs3213207	DTNBP1	BrainSeq	brain	2.2	0.078	0.028	-
rs3213207	DTNBP1	GTEx	brain_nucleus_accumbens	1.93	0.21	0.082	-
rs3213207	DTNBP1	GTEx	brain_cortex	1.56	0.11	0.049	-
rs3213207	DTNBP1	GTEx	brain_hypothalamus	1.42	0.16	0.076	-
rs3213207	DTNBP1	GTEx	brain_putamen	1.33	0.15	0.073	-
rs3213207	DTNBP1	GTEx	brain_amygdala	1.32	0.26	0.13	-
rs3219151	GABRA6	GTEx	brain_cerebellar_hemisphere	2.2	0.21	0.076	-
rs3219151	GABRA6	GTEx	brain_cerebellum	1.6	0.16	0.07	-
rs324420	FAAH	BrainSeq	brain	11.07	-0.14	0.02	-
rs324420	FAAH	ROSMAP	brain_naive	9.67	-0.1	0.016	-
rs324420	FAAH	GTEx	brain_nucleus_accumbens	3.29	-0.16	0.045	-
rs324420	FAAH	GTEx	brain_cerebellum	3.2	-0.13	0.037	-
rs324420	FAAH	GTEx	brain_substantia_nigra	2.14	-0.16	0.057	-
rs324420	FAAH	GTEx	brain_caudate	1.92	-0.11	0.045	-
rs324420	FAAH	GTEx	brain_hypothalamus	1.72	-0.16	0.065	-
rs324420	FAAH	GTEx	brain_cortex	1.58	-0.092	0.041	-
rs324420	FAAH	GTEx	brain_cerebellar_hemisphere	1.34	-0.085	0.042	-
rs324981	NPSR1	GTEx	brain_hypothalamus	1.34	-0.43	0.21	-
rs3742278	HTR2A	ROSMAP	brain_naive	2.58	-0.063	0.021	-
rs3749034	GAD1	GTEx	brain_amygdala	1.92	-0.39	0.15	-
rs3749034	GAD1	GTEx	brain_spinal_cord	1.58	-0.34	0.15	-
rs3813034	SLC6A4	GTEx	brain_cerebellum	1.89	0.12	0.047	-
rs4565946	TPH2	BrainSeq	brain	2.88	0.13	0.039	-
rs4606	RGS2	ROSMAP	brain_naive	1.66	-0.065	0.028	-
rs4606	RGS2	GTEx	brain_hippocampus	1.37	-0.25	0.12	-
rs4606	RGS2	GTEx	brain_anterior_cingulate_cortex	1.36	-0.23	0.11	-
rs4606	RGS2	GTEx	brain_spinal_cord	1.35	-0.3	0.15	-
rs4684677	GHRLOS	GTEx	brain_nucleus_accumbens	1.82	0.51	0.21	-
rs4684677	GHRLOS	GTEx	brain_substantia_nigra	1.57	0.8	0.35	-
rs4722999	CRHR2	ROSMAP	brain_naive	13	-0.23	0.03	-
rs4722999	CRHR2	GTEx	brain_cortex	5.17	-0.35	0.073	-
rs4722999	CRHR2	GTEx	brain_frontal_cortex	2.96	-0.31	0.091	-
rs4746	GLO1	GTEx	brain_anterior_cingulate_cortex	1.63	-0.064	0.028	-
rs4746	GLO1	ROSMAP	brain_naive	1.45	-0.019	0.0088	-
rs4760820	TPH2	BrainSeq	brain	2.92	0.16	0.049	-
rs4795942	TMEM132E	ROSMAP	brain_naive	4.41	-0.11	0.027	-
rs4795942	TMEM132E	GTEx	brain_spinal_cord	1.83	-0.43	0.17	-
rs4795942	TMEM132E	GTEx	brain_cortex	1.51	-0.19	0.088	-
rs5751876	ADORA2A	GTEx	brain_frontal_cortex	2.72	0.31	0.096	-
rs5751876	ADORA2A-AS1	GTEx	brain_putamen	1.3	0.16	0.083	-
rs6265	BDNF-AS	GTEx	brain_frontal_cortex	3.28	-0.15	0.043	-
rs6265	BDNF-AS	GTEx	brain_spinal_cord	1.94	-0.12	0.046	-
rs6265	BDNF-AS	ROSMAP	brain_naive	1.84	-0.041	0.017	-
rs6265	BDNF-AS	GTEx	brain_putamen	1.81	-0.11	0.045	-
rs6265	BDNF	GTEx	brain_cerebellar_hemisphere	1.66	-0.19	0.08	-
rs6295	HTR1A	GTEx	brain_spinal_cord	2.59	-0.56	0.18	-
rs6295	HTR1A	BrainSeq	brain	1.59	0.091	0.041	-

rs6502892	MIR22HG	ROSMAP	brain_naive	2.93	0.13	0.039	-
rs6502892	MIR22HG	GTEx	brain_nucleus_accumbens	1.68	0.15	0.064	-
rs6502892	MIR22HG	BrainSeq	brain	1.58	0.074	0.033	-
rs6502892	MIR22HG	GTEx	brain_caudate	1.37	0.15	0.072	-
rs7103411	BDNF-AS	GTEx	brain_frontal_cortex	3.09	0.14	0.041	-
rs7103411	BDNF-AS	GTEx	brain_putamen	2.46	0.13	0.044	-
rs7103411	BDNF-AS	GTEx	brain_spinal_cord	1.65	0.11	0.046	-
rs7103411	BDNF	GTEx	brain_cerebellar_hemisphere	1.62	0.18	0.078	-
rs7103411	BDNF-AS	ROSMAP	brain_naive	1.59	0.036	0.016	-
rs754635	CCK	GTEx	brain_spinal_cord	1.58	0.58	0.25	-
rs754635	CCK	GTEx	brain_nucleus_accumbens	1.34	-0.33	0.16	-
rs860554	PKP1	GTEx	brain_cerebellum	1.46	0.25	0.11	-
rs941184	CALCOCO1	GTEx	brain_anterior_cingulate_cortex	1.35	-0.28	0.13	-
rs945032	BDKRB2	ROSMAP	brain_naive	2.61	-0.17	0.057	-
rs6265	BDNF-AS	GTEx	brain_frontal_cortex	3.28	-0.15	0.043	-
rs6265	BDNF-AS	GTEx	brain_spinal_cord	1.94	-0.12	0.046	-
rs6265	BDNF-AS	ROSMAP	brain_naive	1.84	-0.041	0.017	-
rs6265	BDNF-AS	GTEx	brain_putamen	1.81	-0.11	0.045	-
rs6265	BDNF	GTEx	brain_cerebellar_hemisphere	1.66	-0.19	0.08	-
rs324981	NPSR1	GTEx	brain_hypothalamus	1.34	-0.43	0.21	-
rs4606	RGS2	ROSMAP	brain_naive	1.66	-0.065	0.028	-
rs4606	RGS2	GTEx	brain_hippocampus	1.37	-0.25	0.12	-
rs4606	RGS2	GTEx	brain_anterior_cingulate_cortex	1.36	-0.23	0.11	-
rs4606	RGS2	GTEx	brain_spinal_cord	1.35	-0.3	0.15	-
rs11568817	HTR1B	BrainSeq	brain	2.66	-0.13	0.043	-
rs78602344	THBS2	ROSMAP	brain_naive	3.04	0.27	0.082	-
rs14259	PSMD9	ROSMAP	brain_naive	10.95	-0.06	0.0087	0.014
rs14259	PSMD9	BrainSeq	brain	4.1	-0.071	0.018	-
rs14259	PSMD9	GTEx	brain_amygdala	2.32	-0.14	0.046	-
rs14259	PSMD9	GTEx	brain_putamen	1.95	-0.094	0.036	-
rs14259	PSMD9	GTEx	brain_hippocampus	1.89	-0.11	0.044	-
rs14259	PSMD9	GTEx	brain_cerebellar_hemisphere	1.65	-0.05	0.021	-
rs14259	PSMD9	GTEx	brain_hypothalamus	1.51	-0.09	0.041	-
rs14259	PSMD9	GTEx	brain_anterior_cingulate_cortex	1.46	-0.1	0.049	-
rs2514259	AIP	BrainSeq	brain	1.9	-0.038	0.015	-
rs2514259	AIP	GTEx	brain_caudate	1.74	-0.18	0.076	-
rs3825172	PSMD9	ROSMAP	brain_naive	11.01	-0.06	0.0087	0.015
rs3825172	PSMD9	BrainSeq	brain	3.75	-0.069	0.018	-
rs3825172	PSMD9	GTEx	brain_hippocampus	2.32	-0.13	0.043	-
rs3825172	PSMD9	GTEx	brain_amygdala	2.32	-0.14	0.046	-
rs3825172	PSMD9	GTEx	brain_putamen	2.04	-0.098	0.037	-
rs3825172	PSMD9	GTEx	brain_cerebellar_hemisphere	1.64	-0.051	0.022	-
rs3825172	PSMD9	GTEx	brain_hypothalamus	1.64	-0.095	0.041	-
rs3825172	PSMD9	GTEx	brain_anterior_cingulate_cortex	1.39	-0.1	0.049	-
rs74421874	PSMD9	ROSMAP	brain_naive	11.01	-0.06	0.0087	0.015
rs74421874	PSMD9	BrainSeq	brain	3.75	-0.069	0.018	-
rs74421874	PSMD9	GTEx	brain_hippocampus	2.32	-0.13	0.043	-
rs74421874	PSMD9	GTEx	brain_amygdala	2.32	-0.14	0.046	-
rs74421874	PSMD9	GTEx	brain_putamen	2.04	-0.098	0.037	-
rs74421874	PSMD9	GTEx	brain_cerebellar_hemisphere	1.64	-0.051	0.022	-
rs74421874	PSMD9	GTEx	brain_hypothalamus	1.64	-0.095	0.041	-

rs74421874	PSMD9	GTEx	brain_anterior_cingulate_cortex	1.39	-0.1	0.049	-
rs1799971	OPRM1	GTEx	brain_cerebellum	3.56	0.28	0.076	-
rs1799971	OPRM1	GTEx	brain_cerebellar_hemisphere	2.77	0.33	0.1	-
rs1799971	OPRM1	GTEx	brain_amygdala	1.68	0.55	0.23	-
rs4792888	CRHR1	GTEx	brain_hippocampus	1.52	0.61	0.28	-
rs4792888	MAPT-AS1	ROSMAP	brain_naive	4.87	0.18	0.042	-
rs4792888	MAPT-AS1	GTEx	brain_substantia_nigra	1.85	0.34	0.13	-
rs4792888	MAPT-AS1	GTEx	brain_nucleus_accumbens	1.33	0.19	0.095	-
rs7997012	HTR2A	ROSMAP	brain_naive	2.1	0.038	0.014	-
rs7997012	HTR2A	GTEx	brain_caudate	2.03	-0.29	0.11	-
rs7997012	HTR2A	GTEx	brain_anterior_cingulate_cortex	1.5	0.16	0.073	-
rs12454712	BCL2	GTEx	brain_cerebellar_hemisphere	1.63	0.081	0.035	-
rs4245146	DRD2	GTEx	brain_cortex	1.38	0.12	0.057	-
rs2476601	PTPN22	GTEx	brain_hippocampus	1.64	-0.51	0.22	-
rs2476601	PTPN22	GTEx	brain_nucleus_accumbens	1.45	-0.32	0.15	-
rs2476601	PTPN22	GTEx	brain_cerebellar_hemisphere	1.3	-0.16	0.083	-
rs2476601	AP4B1-AS1	GTEx	brain_cerebellum	3.06	0.2	0.058	-
rs2476601	AP4B1-AS1	GTEx	brain_substantia_nigra	1.56	0.56	0.25	-
rs2306073	ARNTL2	BrainSeq	brain	1.45	0.046	0.022	-
rs4606	RGS2	ROSMAP	brain_naive	1.66	-0.065	0.028	-
rs4606	RGS2	GTEx	brain_hippocampus	1.37	-0.25	0.12	-
rs4606	RGS2	GTEx	brain_anterior_cingulate_cortex	1.36	-0.23	0.11	-
rs4606	RGS2	GTEx	brain_spinal_cord	1.35	-0.3	0.15	-
rs4964057	ARNTL2	GTEx	brain_substantia_nigra	1.38	-0.2	0.097	-
rs53576	CAV3	GTEx	brain_caudate	2.13	-0.3	0.11	-
rs53576	CAV3	GTEx	brain_cortex	1.5	-0.28	0.13	-
rs53576	OXTR	ROSMAP	brain_naive	21.36	-0.55	0.054	-
rs53576	OXTR	GTEx	brain_caudate	5.64	-0.83	0.17	-
rs53576	OXTR	GTEx	brain_frontal_cortex	4.74	-0.47	0.1	-
rs53576	OXTR	BrainSeq	brain	4.36	-0.21	0.051	-
rs53576	OXTR	GTEx	brain_anterior_cingulate_cortex	3.32	-0.39	0.11	-
rs53576	OXTR	GTEx	brain_cortex	3.04	-0.32	0.094	-
rs53576	OXTR	GTEx	brain_putamen	2.54	-0.45	0.15	-
rs53576	OXTR	GTEx	brain_nucleus_accumbens	2.38	-0.37	0.13	-
rs53576	OXTR	GTEx	brain_substantia_nigra	2.18	-0.56	0.2	-
rs53576	OXTR	GTEx	brain_hypothalamus	2.17	-0.41	0.15	-
rs53576	OXTR	GTEx	brain_hippocampus	1.45	-0.28	0.13	-
rs6855911	SLC2A9	GTEx	brain_cortex	3.11	0.27	0.077	-
rs6855911	SLC2A9	ROSMAP	brain_naive	1.65	0.057	0.025	-
rs7131056	DRD2	ROSMAP	brain_naive	3.21	-0.094	0.027	-
rs7958822	ARNTL2	GTEx	brain_cerebellum	2.17	0.075	0.027	-
rs7958822	ARNTL2	GTEx	brain_amygdala	1.67	-0.18	0.074	-
rs4818048	B3GALT5-AS1	GTEx	brain_nucleus_accumbens	2.21	-0.26	0.095	-
rs4818048	B3GALT5-AS1	GTEx	brain_cerebellar_hemisphere	1.31	-0.39	0.2	-
rs17384439	UNC5C	GTEx	brain_nucleus_accumbens	1.64	0.24	0.1	-
rs581043	CADPS	GTEx	brain_frontal_cortex	1.31	-0.077	0.038	-
rs999719	LARGE1	ROSMAP	brain_naive	1.94	0.03	0.012	-
rs999719	LARGE1	GTEx	brain_hippocampus	1.34	0.12	0.06	-
rs11658311	PEMT	GTEx	brain_nucleus_accumbens	2.65	0.15	0.048	-
rs75494728	LRRC8D	GTEx	brain_putamen	1.39	0.096	0.046	-

rs75494728	ZNF326	GTEx	brain_substantia_nigra	1.52	0.2	0.089	-
rs67366981	TMEM63C	ROSMAP	brain_naive	10.08	0.15	0.023	-
rs67366981	TMEM63C	GTEx	brain_cerebellum	8.54	0.31	0.049	-
rs67366981	TMEM63C	GTEx	brain_cerebellar_hemisphere	3.91	0.28	0.07	-
rs67366981	TMEM63C	GTEx	brain_cortex	2.68	0.2	0.063	-
rs67366981	TMEM63C	BrainSeq	brain	2.47	0.057	0.019	-
rs67366981	TMEM63C	GTEx	brain_amygdala	1.51	0.16	0.072	-
rs297941	FAIM2	GTEx	brain_cortex	1.69	-0.072	0.031	-
rs860554	PKP1	GTEx	brain_cerebellum	1.46	0.25	0.11	-
rs9372078	PLEKHG1	GTEx	brain_spinal_cord	1.38	-0.14	0.069	-
rs17466684	CLU	ROSMAP	brain_naive	2.57	-0.044	0.015	-
rs17466684	CLU	GTEx	brain_cortex	1.56	-0.13	0.057	-
rs941184	CALCOCO1	GTEx	brain_anterior_cingulate_cortex	1.35	-0.28	0.13	-
rs3749380	GRM7	GTEx	brain_cortex	1.71	-0.082	0.034	-
rs2823455	USP25	GTEx	brain_amygdala	2.03	-0.15	0.056	-
rs2823455	USP25	GTEx	brain_caudate	1.77	-0.077	0.032	-
rs2823455	USP25	ROSMAP	brain_naive	1.66	-0.038	0.016	-
rs2823455	USP25	GTEx	brain_frontal_cortex	1.63	-0.07	0.03	-

Supplementary Data File 2: Summary of genes proximal to AD-associated variants showing differential expression in OCD patients. The table provides data on the statistical significance of differential expression for specific genes, represented as p-values, and the corresponding fold changes, which indicate the level of differential gene expression in individuals with OCD compared to those without the condition (GSE6019025).

Gene	P-value	Fold change
SAFB2	0.0015	1.92
NPAS2	0.0002	1.78
NTRK2	0.0213	1.78
RGS2	0.0027	1.7
CACNA2D3	0.0119	1.66
CADPS	0.0343	1.63
FAIM2	0.0009	1.62
SMOX	0.0026	1.62
NTRK3	0.0038	1.6
HTR2A	0.0058	1.59
TNFRSF21	0.0011	1.59
BCL2	0.0028	1.58
FAAH	0.003	1.58
BTBD9	0.0061	1.57
ASIC1	0.004	1.56
CAMK1	0.0017	1.56
PTBP3	0.0132	1.56
ZFR	0.0247	1.55
GPX1	0.005	1.51
ATP6V1G2	0.0308	1.5
CDH10	0.0279	1.5
OLIG2	0.0104	1.5
FAM155A	0.0106	1.49
MTHFR	0.0001	1.48
C7orf50	0.0067	1.47
PTPRD	0.017	1.47
CDH2	0.0049	1.46
GRN	0.0039	1.45
DTNBP1	0.0063	1.44
OXTR	0.0002	1.42
PLEKHG1	0.0028	1.42
CLDN14	0.0054	1.41
EPHX2	0.0181	1.41
CALCOCO1	0.0029	1.39
MAP2K7	0.0028	1.38
DACH1	0.0332	1.37
ADORA2A	0.0038	1.36
LRRC8D	0.0231	1.36
AIP	0.0073	1.35
MEIS2	0.023	1.35
ADCK1	0.0186	1.34
TRMT11	0.0462	1.34
TXNRD2	0.018	1.3

SNAPC2	0.0206	1.29
PRDM11	0.0217	1.28
MIR22HG	0.0487	1.27
MZT1	0.0125	1.27
SDK2	0.0072	1.27
CD320	0.009	1.25
CRHR1	0.0007	1.25
HACE1	0.0195	1.23
NEK11	0.0026	1.23
SNURF	0.02	1.23
